# Supplementary material for: Meta-Analysis and Gene Set Enrichment Relative to ER Status Reveal Elevated Activity of MYC and E2F in the “Basal” Breast Cancer Subgroup
Source: PLoS One. 2009 Mar 9;4(3):e4710. doi: 10.1371/journal.pone.0004710 (PMC2650420; doi:10.1371/journal.pone.0004710)
Supplement: Dataset S1 — GSEA results from first screen. The file contains a zipped archive of the HTML results of the second GSEA screen. Reports for enriched or depleted datasets may be accessed from the “index.html” link within the my_analysis.GseaPreranked.1219975950625.rpt.zip ZIP archive. Enrichment plots are available for each of the 50 most enriched and depleted gene sets. (13.32 MB ZIP) [file pone.0004710.s001.zip › my_analysis.GseaPreranked.1219975950625.rpt.zip!//gsea_report_for_na_pos_1219975950625.html]

Report for na\_pos 1219975950625 [GSEA]

| GS  follow link to MSigDB | GS DETAILS | SIZE | ES | NES | NOM p-val | FDR q-val | FWER p-val | RANK AT MAX | LEADING EDGE || 1 | BRCA\_ER\_NEG | Details ... | 867 | 0.72 | 3.67 | 0.000 | 0.000 | 0.000 | 2670 | tags=72%, list=20%, signal=84% |
| 2 | BRCA\_PROGNOSIS\_NEG | Details ... | 94 | 0.73 | 2.97 | 0.000 | 0.000 | 0.000 | 1891 | tags=66%, list=14%, signal=76% |
| 3 | SERUM\_FIBROBLAST\_CELLCYCLE | Details ... | 110 | 0.71 | 2.94 | 0.000 | 0.000 | 0.000 | 2188 | tags=69%, list=16%, signal=82% |
| 4 | LEE\_TCELLS3\_UP | Details ... | 93 | 0.69 | 2.82 | 0.000 | 0.000 | 0.000 | 1859 | tags=61%, list=14%, signal=71% |
| 5 | IDX\_TSA\_UP\_CLUSTER3 | Details ... | 83 | 0.69 | 2.77 | 0.000 | 0.000 | 0.000 | 1382 | tags=57%, list=10%, signal=63% |
| 6 | VANTVEER\_BREAST\_OUTCOME\_GOOD\_VS\_POOR\_DN | Details ... | 63 | 0.73 | 2.75 | 0.000 | 0.000 | 0.000 | 2047 | tags=70%, list=15%, signal=82% |
| 7 | P21\_P53\_ANY\_DN | Details ... | 47 | 0.76 | 2.73 | 0.000 | 0.000 | 0.000 | 1853 | tags=68%, list=14%, signal=79% |
| 8 | BRCA\_BRCA1\_POS | Details ... | 95 | 0.67 | 2.69 | 0.000 | 0.000 | 0.000 | 3207 | tags=68%, list=24%, signal=89% |
| 9 | CANCER\_UNDIFFERENTIATED\_META\_UP | Details ... | 67 | 0.70 | 2.69 | 0.000 | 0.000 | 0.000 | 2127 | tags=69%, list=16%, signal=81% |
| 10 | ZHAN\_MM\_CD138\_PR\_VS\_REST | Details ... | 42 | 0.74 | 2.61 | 0.000 | 0.000 | 0.000 | 1724 | tags=71%, list=13%, signal=82% |
| 11 | CELL\_CYCLE | Details ... | 74 | 0.64 | 2.52 | 0.000 | 0.000 | 0.000 | 3185 | tags=66%, list=24%, signal=87% |
| 12 | CMV\_IE86\_UP | Details ... | 49 | 0.69 | 2.52 | 0.000 | 0.000 | 0.000 | 1438 | tags=59%, list=11%, signal=66% |
| 13 | HOFFMANN\_BIVSBII\_BI\_TABLE2 | Details ... | 184 | 0.56 | 2.50 | 0.000 | 0.000 | 0.000 | 1827 | tags=42%, list=14%, signal=48% |
| 14 | CELL\_CYCLE\_KEGG | Details ... | 80 | 0.63 | 2.50 | 0.000 | 0.000 | 0.000 | 3185 | tags=63%, list=24%, signal=82% |
| 15 | DOX\_RESIST\_GASTRIC\_UP | Details ... | 43 | 0.70 | 2.49 | 0.000 | 0.000 | 0.000 | 2047 | tags=72%, list=15%, signal=85% |
| 16 | TARTE\_PLASMA\_BLASTIC | Details ... | 305 | 0.52 | 2.48 | 0.000 | 0.000 | 0.000 | 3555 | tags=55%, list=27%, signal=73% |
| 17 | CROONQUIST\_IL6\_STARVE\_UP | Details ... | 33 | 0.74 | 2.47 | 0.000 | 0.000 | 0.000 | 2047 | tags=73%, list=15%, signal=86% |
| 18 | LI\_FETAL\_VS\_WT\_KIDNEY\_DN | Details ... | 159 | 0.56 | 2.46 | 0.000 | 0.000 | 0.000 | 1870 | tags=48%, list=14%, signal=56% |
| 19 | ADIP\_DIFF\_CLUSTER5 | Details ... | 35 | 0.73 | 2.46 | 0.000 | 0.000 | 0.000 | 2010 | tags=69%, list=15%, signal=81% |
| 20 | VERNELL\_PRB\_CLSTR1 | Details ... | 53 | 0.66 | 2.45 | 0.000 | 0.000 | 0.000 | 1532 | tags=51%, list=12%, signal=57% |
| 21 | LE\_MYELIN\_UP | Details ... | 82 | 0.61 | 2.45 | 0.000 | 0.000 | 0.000 | 1243 | tags=44%, list=9%, signal=48% |
| 22 | P21\_P53\_MIDDLE\_DN | Details ... | 23 | 0.78 | 2.41 | 0.000 | 0.000 | 0.000 | 1853 | tags=74%, list=14%, signal=86% |
| 23 | HCC\_SURVIVAL\_GOOD\_VS\_POOR\_DN | Details ... | 121 | 0.57 | 2.40 | 0.000 | 0.000 | 0.000 | 2669 | tags=50%, list=20%, signal=62% |
| 24 | GOLDRATH\_CELLCYCLE | Details ... | 27 | 0.75 | 2.39 | 0.000 | 0.000 | 0.000 | 988 | tags=67%, list=7%, signal=72% |
| 25 | OLDAGE\_DN | Details ... | 47 | 0.67 | 2.39 | 0.000 | 0.000 | 0.000 | 2105 | tags=64%, list=16%, signal=76% |
| 26 | SHEPARD\_CRASH\_AND\_BURN\_MUT\_VS\_WT\_DN | Details ... | 137 | 0.56 | 2.39 | 0.000 | 0.000 | 0.000 | 2037 | tags=40%, list=15%, signal=47% |
| 27 | SERUM\_FIBROBLAST\_CORE\_UP | Details ... | 174 | 0.53 | 2.36 | 0.000 | 0.000 | 0.000 | 2760 | tags=45%, list=21%, signal=56% |
| 28 | YU\_CMYC\_UP | Details ... | 27 | 0.73 | 2.32 | 0.000 | 0.000 | 0.000 | 2026 | tags=70%, list=15%, signal=83% |
| 29 | CROONQUIST\_IL6\_RAS\_DN | Details ... | 24 | 0.74 | 2.32 | 0.000 | 0.000 | 0.000 | 1827 | tags=75%, list=14%, signal=87% |
| 30 | MANALO\_HYPOXIA\_DN | Details ... | 78 | 0.58 | 2.31 | 0.000 | 0.000 | 0.000 | 2151 | tags=51%, list=16%, signal=61% |
| 31 | SHEPARD\_GENES\_COMMON\_BW\_CB\_MO | Details ... | 60 | 0.61 | 2.30 | 0.000 | 0.000 | 0.000 | 1820 | tags=48%, list=14%, signal=56% |
| 32 | SGCGSSAAA\_V$E2F1DP2\_01 | Details ... | 114 | 0.54 | 2.27 | 0.000 | 0.000 | 0.000 | 2348 | tags=43%, list=18%, signal=52% |
| 33 | REN\_E2F1\_TARGETS | Details ... | 36 | 0.66 | 2.26 | 0.000 | 0.000 | 0.000 | 1346 | tags=47%, list=10%, signal=52% |
| 34 | BREASTCA\_TWO\_CLASSES | Details ... | 131 | 0.53 | 2.25 | 0.000 | 0.000 | 0.000 | 1868 | tags=47%, list=14%, signal=54% |
| 35 | GENOTOXINS\_ALL\_4HRS\_REG | Details ... | 22 | 0.74 | 2.23 | 0.000 | 0.000 | 0.000 | 2026 | tags=64%, list=15%, signal=75% |
| 36 | ZHAN\_MULTIPLE\_MYELOMA\_SUBCLASSES\_DIFF | Details ... | 30 | 0.68 | 2.22 | 0.000 | 0.000 | 0.000 | 1939 | tags=57%, list=15%, signal=66% |
| 37 | GAY\_YY1\_DN | Details ... | 220 | 0.48 | 2.21 | 0.000 | 0.000 | 0.000 | 2135 | tags=35%, list=16%, signal=42% |
| 38 | V$E2F1\_Q6 | Details ... | 155 | 0.50 | 2.20 | 0.000 | 0.000 | 0.000 | 2413 | tags=41%, list=18%, signal=49% |
| 39 | V$E2F1\_Q6\_01 | Details ... | 167 | 0.49 | 2.18 | 0.000 | 0.000 | 0.000 | 2649 | tags=42%, list=20%, signal=52% |
| 40 | V$E2F1DP2\_01 | Details ... | 157 | 0.49 | 2.16 | 0.000 | 0.000 | 0.000 | 2413 | tags=41%, list=18%, signal=49% |
| 41 | GREENBAUM\_E2A\_UP | Details ... | 32 | 0.65 | 2.16 | 0.000 | 0.000 | 0.000 | 2026 | tags=66%, list=15%, signal=77% |
| 42 | RUIZ\_TENASCIN\_TARGETS | Details ... | 79 | 0.54 | 2.15 | 0.000 | 0.000 | 0.001 | 2188 | tags=43%, list=16%, signal=51% |
| 43 | V$E2F\_02 | Details ... | 158 | 0.48 | 2.14 | 0.000 | 0.000 | 0.003 | 2413 | tags=41%, list=18%, signal=49% |
| 44 | V$E2F1DP1\_01 | Details ... | 157 | 0.49 | 2.14 | 0.000 | 0.000 | 0.003 | 2413 | tags=41%, list=18%, signal=49% |
| 45 | V$E2F4DP1\_01 | Details ... | 161 | 0.48 | 2.13 | 0.000 | 0.000 | 0.003 | 2473 | tags=40%, list=19%, signal=49% |
| 46 | CHANG\_SERUM\_RESPONSE\_UP | Details ... | 143 | 0.49 | 2.13 | 0.000 | 0.000 | 0.003 | 2760 | tags=45%, list=21%, signal=56% |
| 47 | DNA\_REPLICATION\_REACTOME | Details ... | 44 | 0.60 | 2.13 | 0.000 | 0.000 | 0.003 | 1346 | tags=45%, list=10%, signal=50% |
| 48 | V$E2F1\_Q3 | Details ... | 156 | 0.48 | 2.12 | 0.000 | 0.000 | 0.004 | 2585 | tags=39%, list=19%, signal=48% |
| 49 | V$E2F4DP2\_01 | Details ... | 157 | 0.49 | 2.12 | 0.000 | 0.000 | 0.005 | 2413 | tags=41%, list=18%, signal=49% |
| 50 | PENG\_LEUCINE\_DN | Details ... | 139 | 0.49 | 2.11 | 0.000 | 0.000 | 0.005 | 2792 | tags=45%, list=21%, signal=56% |
| 51 | CANCER\_NEOPLASTIC\_META\_UP |  | 60 | 0.54 | 2.10 | 0.000 | 0.000 | 0.013 | 3105 | tags=57%, list=23%, signal=74% |
| 52 | PENG\_GLUTAMINE\_DN |  | 248 | 0.46 | 2.10 | 0.000 | 0.000 | 0.013 | 2812 | tags=40%, list=21%, signal=50% |
| 53 | HINATA\_NFKB\_UP |  | 105 | 0.50 | 2.08 | 0.000 | 0.000 | 0.016 | 1940 | tags=40%, list=15%, signal=46% |
| 54 | SCHUMACHER\_MYC\_UP |  | 50 | 0.57 | 2.07 | 0.000 | 0.000 | 0.020 | 1699 | tags=48%, list=13%, signal=55% |
| 55 | HG\_PROGERIA\_DN |  | 25 | 0.67 | 2.06 | 0.000 | 0.000 | 0.021 | 1202 | tags=56%, list=9%, signal=61% |
| 56 | PENG\_RAPAMYCIN\_DN |  | 188 | 0.46 | 2.06 | 0.000 | 0.000 | 0.025 | 2964 | tags=42%, list=22%, signal=53% |
| 57 | BLEO\_MOUSE\_LYMPH\_HIGH\_24HRS\_DN |  | 34 | 0.60 | 2.04 | 0.000 | 0.001 | 0.036 | 2764 | tags=59%, list=21%, signal=74% |
| 58 | BRENTANI\_CELL\_CYCLE |  | 80 | 0.52 | 2.04 | 0.000 | 0.001 | 0.038 | 2231 | tags=44%, list=17%, signal=52% |
| 59 | P21\_MIDDLE\_DN |  | 15 | 0.73 | 2.02 | 0.000 | 0.001 | 0.050 | 2012 | tags=67%, list=15%, signal=78% |
| 60 | MRNA\_SPLICING |  | 47 | 0.55 | 2.01 | 0.000 | 0.001 | 0.059 | 2898 | tags=53%, list=22%, signal=68% |
| 61 | IRITANI\_ADPROX\_UP |  | 26 | 0.64 | 2.01 | 0.000 | 0.001 | 0.061 | 3883 | tags=73%, list=29%, signal=103% |
| 62 | AGUIRRE\_PANCREAS\_CHR9 |  | 24 | 0.65 | 2.00 | 0.000 | 0.001 | 0.069 | 3096 | tags=67%, list=23%, signal=87% |
| 63 | SANA\_TNFA\_ENDOTHELIAL\_UP |  | 75 | 0.51 | 2.00 | 0.000 | 0.001 | 0.069 | 3145 | tags=51%, list=24%, signal=66% |
| 64 | LEE\_TCELLS2\_UP |  | 939 | 0.39 | 2.00 | 0.000 | 0.001 | 0.069 | 2730 | tags=36%, list=20%, signal=43% |
| 65 | STEMCELL\_EMBRYONIC\_UP |  | 1165 | 0.39 | 2.00 | 0.000 | 0.001 | 0.076 | 2788 | tags=35%, list=21%, signal=40% |
| 66 | JECHLINGER\_EMT\_UP |  | 53 | 0.54 | 1.99 | 0.000 | 0.001 | 0.081 | 1454 | tags=32%, list=11%, signal=36% |
| 67 | ZUCCHI\_EPITHELIAL\_DN |  | 41 | 0.56 | 1.98 | 0.000 | 0.002 | 0.089 | 1523 | tags=39%, list=11%, signal=44% |
| 68 | SHEPARD\_BMYB\_MORPHOLINO\_DN |  | 152 | 0.45 | 1.98 | 0.000 | 0.002 | 0.093 | 1820 | tags=32%, list=14%, signal=37% |
| 69 | BREAST\_DUCTAL\_CARCINOMA\_GENES |  | 18 | 0.68 | 1.98 | 0.000 | 0.002 | 0.093 | 2047 | tags=61%, list=15%, signal=72% |
| 70 | BREASTCA\_THREE\_CLASSES |  | 41 | 0.57 | 1.97 | 0.000 | 0.002 | 0.104 | 1644 | tags=46%, list=12%, signal=53% |
| 71 | BASSO\_REGULATORY\_HUBS |  | 138 | 0.46 | 1.97 | 0.000 | 0.002 | 0.105 | 1669 | tags=36%, list=13%, signal=41% |
| 72 | P21\_ANY\_DN |  | 32 | 0.60 | 1.97 | 0.000 | 0.002 | 0.116 | 2012 | tags=47%, list=15%, signal=55% |
| 73 | V$E2F\_01 |  | 54 | 0.52 | 1.97 | 0.000 | 0.002 | 0.128 | 1820 | tags=39%, list=14%, signal=45% |
| 74 | MMS\_HUMAN\_LYMPH\_LOW\_4HRS\_DN |  | 16 | 0.72 | 1.97 | 0.000 | 0.002 | 0.128 | 1511 | tags=44%, list=11%, signal=49% |
| 75 | ELONGINA\_KO\_DN |  | 154 | 0.45 | 1.97 | 0.000 | 0.002 | 0.128 | 2577 | tags=42%, list=19%, signal=51% |
| 76 | CARIES\_PULP\_HIGH\_UP |  | 89 | 0.48 | 1.96 | 0.000 | 0.002 | 0.134 | 4451 | tags=63%, list=33%, signal=94% |
| 77 | CHR1P34 |  | 107 | 0.47 | 1.96 | 0.000 | 0.002 | 0.134 | 2684 | tags=43%, list=20%, signal=53% |
| 78 | PARK\_RARALPHA\_MOD |  | 60 | 0.51 | 1.95 | 0.000 | 0.003 | 0.156 | 3139 | tags=48%, list=24%, signal=63% |
| 79 | IGFR\_IR\_UP |  | 16 | 0.70 | 1.95 | 0.000 | 0.003 | 0.166 | 2792 | tags=69%, list=21%, signal=87% |
| 80 | TNFA\_NFKB\_DEP\_UP |  | 18 | 0.68 | 1.94 | 0.002 | 0.003 | 0.192 | 3129 | tags=78%, list=23%, signal=102% |
| 81 | LINDSTEDT\_DEND\_8H\_VS\_48H\_UP |  | 64 | 0.51 | 1.93 | 0.000 | 0.003 | 0.203 | 3145 | tags=56%, list=24%, signal=73% |
| 82 | SHIPP\_FL\_VS\_DLBCL\_DN |  | 34 | 0.58 | 1.93 | 0.000 | 0.003 | 0.210 | 2398 | tags=47%, list=18%, signal=57% |
| 83 | V$E2F\_Q6 |  | 158 | 0.43 | 1.92 | 0.000 | 0.004 | 0.242 | 2674 | tags=38%, list=20%, signal=47% |
| 84 | MYC\_TARGETS |  | 39 | 0.56 | 1.92 | 0.002 | 0.004 | 0.250 | 2647 | tags=46%, list=20%, signal=57% |
| 85 | HALMOS\_CEBP\_UP |  | 50 | 0.53 | 1.92 | 0.001 | 0.004 | 0.266 | 2126 | tags=44%, list=16%, signal=52% |
| 86 | V$E2F\_Q4 |  | 160 | 0.43 | 1.91 | 0.000 | 0.004 | 0.286 | 2674 | tags=38%, list=20%, signal=46% |
| 87 | ADIP\_DIFF\_CLUSTER4 |  | 32 | 0.57 | 1.91 | 0.000 | 0.005 | 0.297 | 1558 | tags=47%, list=12%, signal=53% |
| 88 | CHR10P13 |  | 17 | 0.66 | 1.90 | 0.000 | 0.005 | 0.308 | 2118 | tags=65%, list=16%, signal=77% |
| 89 | ERM\_KO\_TESTES\_DN |  | 17 | 0.66 | 1.90 | 0.003 | 0.005 | 0.310 | 2688 | tags=59%, list=20%, signal=74% |
| 90 | CHR11Q21 |  | 21 | 0.63 | 1.90 | 0.002 | 0.005 | 0.344 | 2251 | tags=48%, list=17%, signal=57% |
| 91 | V$E2F\_03 |  | 172 | 0.42 | 1.89 | 0.000 | 0.005 | 0.355 | 2844 | tags=37%, list=21%, signal=46% |
| 92 | V$E2F\_Q4\_01 |  | 164 | 0.43 | 1.89 | 0.000 | 0.006 | 0.369 | 3190 | tags=43%, list=24%, signal=55% |
| 93 | CHR2P21 |  | 28 | 0.59 | 1.88 | 0.002 | 0.006 | 0.423 | 2580 | tags=50%, list=19%, signal=62% |
| 94 | CHR6Q21 |  | 36 | 0.56 | 1.88 | 0.000 | 0.006 | 0.425 | 2807 | tags=58%, list=21%, signal=74% |
| 95 | INOS\_ALL\_UP |  | 52 | 0.51 | 1.87 | 0.000 | 0.007 | 0.436 | 2942 | tags=44%, list=22%, signal=57% |
| 96 | HUMAN\_TISSUE\_TESTIS |  | 59 | 0.50 | 1.87 | 0.000 | 0.007 | 0.436 | 1882 | tags=36%, list=14%, signal=41% |
| 97 | BAF57\_BT549\_DN |  | 313 | 0.39 | 1.86 | 0.000 | 0.008 | 0.495 | 3217 | tags=40%, list=24%, signal=51% |
| 98 | CHR7Q32 |  | 30 | 0.56 | 1.86 | 0.000 | 0.008 | 0.498 | 3963 | tags=60%, list=30%, signal=85% |
| 99 | MENSSEN\_MYC\_UP |  | 31 | 0.57 | 1.86 | 0.000 | 0.008 | 0.498 | 2585 | tags=45%, list=19%, signal=56% |
| 100 | V$E2F1\_Q4\_01 |  | 157 | 0.42 | 1.85 | 0.000 | 0.008 | 0.526 | 2649 | tags=38%, list=20%, signal=46% |
| 101 | V$E2F\_Q3 |  | 154 | 0.42 | 1.85 | 0.000 | 0.009 | 0.544 | 2674 | tags=36%, list=20%, signal=45% |
| 102 | NEMETH\_TNF\_DN |  | 29 | 0.58 | 1.85 | 0.000 | 0.009 | 0.560 | 1428 | tags=34%, list=11%, signal=39% |
| 103 | V$E2F\_Q3\_01 |  | 157 | 0.42 | 1.84 | 0.000 | 0.009 | 0.580 | 3185 | tags=43%, list=24%, signal=55% |
| 104 | HESS\_HOXAANMEIS1\_UP |  | 55 | 0.49 | 1.84 | 0.000 | 0.010 | 0.599 | 1964 | tags=36%, list=15%, signal=42% |
| 105 | CIS\_XPC\_UP |  | 143 | 0.43 | 1.84 | 0.000 | 0.010 | 0.599 | 2719 | tags=41%, list=20%, signal=51% |
| 106 | SANSOM\_APC\_LOSS5\_UP |  | 70 | 0.48 | 1.84 | 0.000 | 0.010 | 0.600 | 1822 | tags=36%, list=14%, signal=41% |
| 107 | BRCA1\_OVEREXP\_DN |  | 109 | 0.44 | 1.84 | 0.000 | 0.010 | 0.601 | 3082 | tags=44%, list=23%, signal=57% |
| 108 | AGUIRRE\_PANCREAS\_CHR6 |  | 32 | 0.55 | 1.83 | 0.002 | 0.010 | 0.624 | 3551 | tags=63%, list=27%, signal=85% |
| 109 | CHR2P16 |  | 21 | 0.62 | 1.83 | 0.002 | 0.010 | 0.635 | 3116 | tags=67%, list=23%, signal=87% |
| 110 | CHR9Q21 |  | 31 | 0.56 | 1.83 | 0.000 | 0.010 | 0.637 | 2114 | tags=42%, list=16%, signal=50% |
| 111 | CHR2P13 |  | 53 | 0.49 | 1.83 | 0.000 | 0.011 | 0.656 | 3095 | tags=42%, list=23%, signal=54% |
| 112 | G2PATHWAY |  | 23 | 0.61 | 1.83 | 0.000 | 0.011 | 0.662 | 2517 | tags=48%, list=19%, signal=59% |
| 113 | CHR21Q22 |  | 120 | 0.43 | 1.82 | 0.000 | 0.011 | 0.680 | 3175 | tags=41%, list=24%, signal=53% |
| 114 | COLLER\_MYC\_UP |  | 17 | 0.64 | 1.82 | 0.002 | 0.011 | 0.693 | 2812 | tags=59%, list=21%, signal=74% |
| 115 | G1\_TO\_S\_CELL\_CYCLE\_REACTOME |  | 64 | 0.47 | 1.81 | 0.000 | 0.012 | 0.718 | 3148 | tags=52%, list=24%, signal=67% |
| 116 | HESS\_HOXAANMEIS1\_DN |  | 55 | 0.49 | 1.81 | 0.000 | 0.012 | 0.730 | 1964 | tags=36%, list=15%, signal=42% |
| 117 | ALCALAY\_AML\_NPMC\_DN |  | 187 | 0.40 | 1.81 | 0.000 | 0.013 | 0.763 | 2585 | tags=38%, list=19%, signal=46% |
| 118 | CHR12P13 |  | 128 | 0.42 | 1.80 | 0.000 | 0.013 | 0.775 | 2699 | tags=34%, list=20%, signal=42% |
| 119 | ATRBRCAPATHWAY |  | 20 | 0.61 | 1.80 | 0.000 | 0.013 | 0.776 | 2282 | tags=55%, list=17%, signal=66% |
| 120 | CHIARETTI\_T\_ALL |  | 254 | 0.39 | 1.80 | 0.000 | 0.014 | 0.779 | 2838 | tags=35%, list=21%, signal=43% |
| 121 | V$E2F\_Q6\_01 |  | 165 | 0.41 | 1.80 | 0.000 | 0.014 | 0.794 | 3185 | tags=40%, list=24%, signal=52% |
| 122 | HDACI\_COLON\_BUT12HRS\_DN |  | 66 | 0.46 | 1.79 | 0.001 | 0.015 | 0.814 | 2753 | tags=47%, list=21%, signal=59% |
| 123 | V$MYC\_Q2 |  | 127 | 0.41 | 1.79 | 0.000 | 0.016 | 0.835 | 2628 | tags=34%, list=20%, signal=42% |
| 124 | V$E2F1DP1RB\_01 |  | 157 | 0.40 | 1.78 | 0.000 | 0.016 | 0.843 | 3563 | tags=45%, list=27%, signal=60% |
| 125 | CARIES\_PULP\_UP |  | 203 | 0.39 | 1.78 | 0.000 | 0.016 | 0.849 | 3884 | tags=49%, list=29%, signal=68% |
| 126 | FERRANDO\_MLL\_T\_ALL\_DN |  | 83 | 0.44 | 1.77 | 0.000 | 0.018 | 0.872 | 3125 | tags=46%, list=23%, signal=59% |
| 127 | MCAATNNNNNGCG\_UNKNOWN |  | 65 | 0.46 | 1.77 | 0.000 | 0.018 | 0.881 | 3771 | tags=46%, list=28%, signal=64% |
| 128 | DAC\_PANC50\_UP |  | 42 | 0.50 | 1.77 | 0.003 | 0.019 | 0.892 | 3285 | tags=55%, list=25%, signal=72% |
| 129 | CATTGTYY\_V$SOX9\_B1 |  | 257 | 0.38 | 1.76 | 0.000 | 0.019 | 0.902 | 3729 | tags=40%, list=28%, signal=55% |
| 130 | TAKEDA\_NUP8\_HOXA9\_8D\_DN |  | 180 | 0.39 | 1.76 | 0.000 | 0.020 | 0.908 | 3494 | tags=44%, list=26%, signal=59% |
| 131 | CHR11Q22 |  | 33 | 0.52 | 1.76 | 0.002 | 0.020 | 0.912 | 2787 | tags=58%, list=21%, signal=73% |
| 132 | AS3\_FIBRO\_C4 |  | 18 | 0.61 | 1.76 | 0.009 | 0.020 | 0.914 | 1318 | tags=33%, list=10%, signal=37% |
| 133 | STRESS\_TPA\_SPECIFIC\_UP |  | 40 | 0.50 | 1.75 | 0.001 | 0.021 | 0.925 | 2398 | tags=50%, list=18%, signal=61% |
| 134 | ADIP\_DIFF\_CLUSTER3 |  | 32 | 0.53 | 1.75 | 0.003 | 0.021 | 0.930 | 4197 | tags=66%, list=32%, signal=96% |
| 135 | STRESS\_GENOTOXIC\_SPECIFIC\_DN |  | 39 | 0.51 | 1.75 | 0.003 | 0.021 | 0.931 | 3105 | tags=51%, list=23%, signal=67% |
| 136 | CYTOKINEPATHWAY |  | 20 | 0.59 | 1.75 | 0.005 | 0.021 | 0.932 | 2309 | tags=35%, list=17%, signal=42% |
| 137 | PEART\_HISTONE\_DN |  | 71 | 0.46 | 1.75 | 0.001 | 0.021 | 0.938 | 3975 | tags=54%, list=30%, signal=76% |
| 138 | IRITANI\_ADPROX\_LYMPH |  | 125 | 0.41 | 1.75 | 0.000 | 0.021 | 0.938 | 3072 | tags=45%, list=23%, signal=58% |
| 139 | NGUYEN\_KERATO\_DN |  | 80 | 0.44 | 1.75 | 0.001 | 0.021 | 0.940 | 2467 | tags=34%, list=19%, signal=41% |
| 140 | SIG\_REGULATION\_OF\_THE\_ACTIN\_CYTOSKELETON\_BY\_RHO\_GTPASES |  | 33 | 0.52 | 1.75 | 0.005 | 0.022 | 0.948 | 1895 | tags=36%, list=14%, signal=42% |
| 141 | SASAKI\_TCELL\_LYMPHOMA\_VS\_CD4\_UP |  | 167 | 0.39 | 1.74 | 0.000 | 0.022 | 0.953 | 2108 | tags=31%, list=16%, signal=37% |
| 142 | BHATTACHARYA\_ESC\_UP |  | 57 | 0.47 | 1.74 | 0.001 | 0.022 | 0.953 | 2004 | tags=40%, list=15%, signal=47% |
| 143 | LINDSTEDT\_DEND\_UP |  | 50 | 0.47 | 1.74 | 0.004 | 0.023 | 0.959 | 3824 | tags=58%, list=29%, signal=81% |
| 144 | CHR6Q16 |  | 17 | 0.61 | 1.74 | 0.003 | 0.023 | 0.961 | 4153 | tags=71%, list=31%, signal=102% |
| 145 | CMV\_8HRS\_UP |  | 31 | 0.52 | 1.74 | 0.002 | 0.023 | 0.962 | 1340 | tags=29%, list=10%, signal=32% |
| 146 | CHR3Q29 |  | 24 | 0.54 | 1.73 | 0.005 | 0.024 | 0.969 | 3169 | tags=54%, list=24%, signal=71% |
| 147 | EMT\_UP |  | 60 | 0.46 | 1.73 | 0.000 | 0.025 | 0.970 | 2118 | tags=33%, list=16%, signal=39% |
| 148 | TH1TH2PATHWAY |  | 17 | 0.60 | 1.73 | 0.006 | 0.025 | 0.973 | 4104 | tags=59%, list=31%, signal=85% |
| 149 | SASAKI\_ATL\_UP |  | 167 | 0.39 | 1.73 | 0.000 | 0.025 | 0.976 | 2108 | tags=31%, list=16%, signal=37% |
| 150 | CHR22Q13 |  | 123 | 0.41 | 1.73 | 0.003 | 0.025 | 0.976 | 4513 | tags=50%, list=34%, signal=76% |
| 151 | FALT\_BCLL\_UP |  | 44 | 0.48 | 1.73 | 0.006 | 0.026 | 0.978 | 3110 | tags=52%, list=23%, signal=68% |
| 152 | VEGF\_MMMEC\_3HRS\_UP |  | 64 | 0.45 | 1.72 | 0.006 | 0.026 | 0.983 | 2015 | tags=34%, list=15%, signal=40% |
| 153 | RADIATION\_SENSITIVITY |  | 24 | 0.56 | 1.72 | 0.006 | 0.028 | 0.988 | 2854 | tags=42%, list=21%, signal=53% |
| 154 | KTGGYRSGAA\_UNKNOWN |  | 48 | 0.49 | 1.71 | 0.002 | 0.028 | 0.990 | 2314 | tags=38%, list=17%, signal=45% |
| 155 | FERNANDEZ\_MYC\_TARGETS |  | 178 | 0.39 | 1.71 | 0.000 | 0.029 | 0.990 | 2225 | tags=28%, list=17%, signal=33% |
| 156 | ACTINYPATHWAY |  | 16 | 0.61 | 1.71 | 0.007 | 0.029 | 0.990 | 1189 | tags=44%, list=9%, signal=48% |
| 157 | STURLA\_SONIC\_HEDGEHOG |  | 16 | 0.62 | 1.71 | 0.008 | 0.029 | 0.990 | 2688 | tags=50%, list=20%, signal=63% |
| 158 | VERHAAK\_AML\_NPM1\_MUT\_VS\_WT\_UP |  | 189 | 0.38 | 1.71 | 0.000 | 0.029 | 0.992 | 3312 | tags=43%, list=25%, signal=56% |
| 159 | ZELLER\_MYC\_UP |  | 23 | 0.56 | 1.71 | 0.002 | 0.029 | 0.992 | 2647 | tags=43%, list=20%, signal=54% |
| 160 | OKUMURA\_MC\_LPS |  | 185 | 0.38 | 1.70 | 0.000 | 0.031 | 0.993 | 3233 | tags=39%, list=24%, signal=51% |
| 161 | CHIARETTI\_T\_ALL\_DIFF |  | 277 | 0.36 | 1.70 | 0.000 | 0.031 | 0.993 | 2838 | tags=33%, list=21%, signal=41% |
| 162 | CHR3Q25 |  | 34 | 0.51 | 1.70 | 0.003 | 0.032 | 0.995 | 2872 | tags=50%, list=22%, signal=64% |
| 163 | ICHIBA\_GVHD |  | 216 | 0.37 | 1.70 | 0.000 | 0.033 | 0.995 | 2807 | tags=35%, list=21%, signal=43% |
| 164 | MATSUDA\_VALPHAINKT\_DIFF |  | 361 | 0.35 | 1.70 | 0.000 | 0.033 | 0.995 | 2424 | tags=30%, list=18%, signal=36% |
| 165 | WALLACE\_JAK2\_DIFF |  | 30 | 0.52 | 1.70 | 0.002 | 0.032 | 0.995 | 943 | tags=27%, list=7%, signal=29% |
| 166 | CHR2P25 |  | 35 | 0.50 | 1.70 | 0.003 | 0.033 | 0.995 | 2358 | tags=37%, list=18%, signal=45% |
| 167 | FLECHNER\_KIDNEY\_TRANSPLANT\_REJECTION\_UP |  | 86 | 0.42 | 1.69 | 0.000 | 0.033 | 0.995 | 3780 | tags=52%, list=28%, signal=73% |
| 168 | IL1\_CORNEA\_UP |  | 62 | 0.45 | 1.69 | 0.000 | 0.033 | 0.995 | 3245 | tags=52%, list=24%, signal=68% |
| 169 | ACAGGGT,MIR-10A,MIR-10B |  | 95 | 0.41 | 1.69 | 0.001 | 0.035 | 0.996 | 3573 | tags=42%, list=27%, signal=57% |
| 170 | CMV\_HCMV\_TIMECOURSE\_24HRS\_UP |  | 72 | 0.44 | 1.69 | 0.001 | 0.035 | 0.998 | 3105 | tags=49%, list=23%, signal=63% |
| 171 | FLECHNER\_KIDNEY\_TRANSPLANT\_WELL\_PBL\_DN |  | 42 | 0.49 | 1.69 | 0.003 | 0.035 | 0.999 | 2215 | tags=38%, list=17%, signal=46% |
| 172 | PRMT5\_KD\_UP |  | 166 | 0.38 | 1.68 | 0.000 | 0.036 | 0.999 | 2611 | tags=33%, list=20%, signal=41% |
| 173 | UVB\_NHEK3\_C7 |  | 53 | 0.45 | 1.67 | 0.003 | 0.040 | 0.999 | 2265 | tags=43%, list=17%, signal=52% |
| 174 | TPA\_SENS\_MIDDLE\_UP |  | 65 | 0.44 | 1.67 | 0.003 | 0.040 | 0.999 | 3066 | tags=38%, list=23%, signal=50% |
| 175 | LIAN\_MYELOID\_DIFF\_RECEPTORS |  | 27 | 0.52 | 1.67 | 0.012 | 0.041 | 1.000 | 4648 | tags=74%, list=35%, signal=114% |
| 176 | HIPPOCAMPUS\_DEVELOPMENT\_NEONATAL |  | 26 | 0.53 | 1.67 | 0.014 | 0.041 | 1.000 | 3611 | tags=62%, list=27%, signal=84% |
| 177 | IDX\_TSA\_DN\_CLUSTER6 |  | 19 | 0.56 | 1.67 | 0.008 | 0.042 | 1.000 | 1022 | tags=21%, list=8%, signal=23% |
| 178 | WIELAND\_HEPATITIS\_B\_INDUCED |  | 106 | 0.40 | 1.67 | 0.000 | 0.042 | 1.000 | 4319 | tags=52%, list=32%, signal=76% |
| 179 | CMV\_HCMV\_TIMECOURSE\_8HRS\_DN |  | 16 | 0.60 | 1.67 | 0.015 | 0.042 | 1.000 | 2058 | tags=50%, list=15%, signal=59% |
| 180 | DAC\_IFN\_BLADDER\_UP |  | 16 | 0.60 | 1.66 | 0.010 | 0.043 | 1.000 | 1340 | tags=38%, list=10%, signal=42% |
| 181 | CHR7Q33 |  | 15 | 0.61 | 1.65 | 0.015 | 0.046 | 1.000 | 1931 | tags=40%, list=14%, signal=47% |
| 182 | POD1\_KO\_UP |  | 339 | 0.35 | 1.65 | 0.000 | 0.046 | 1.000 | 1855 | tags=27%, list=14%, signal=31% |
| 183 | AS3\_FIBRO\_DN |  | 31 | 0.51 | 1.65 | 0.010 | 0.047 | 1.000 | 1775 | tags=35%, list=13%, signal=41% |
| 184 | GCAAGAC,MIR-431 |  | 28 | 0.51 | 1.65 | 0.008 | 0.047 | 1.000 | 2085 | tags=43%, list=16%, signal=51% |
| 185 | CHR6P21 |  | 253 | 0.36 | 1.65 | 0.000 | 0.046 | 1.000 | 3951 | tags=41%, list=30%, signal=57% |
| 186 | NEMETH\_TNF\_UP |  | 77 | 0.42 | 1.65 | 0.003 | 0.047 | 1.000 | 2043 | tags=34%, list=15%, signal=40% |
| 187 | POMEROY\_DESMOPLASIC\_VS\_CLASSIC\_MD\_UP |  | 46 | 0.46 | 1.65 | 0.006 | 0.049 | 1.000 | 1893 | tags=35%, list=14%, signal=40% |
| 188 | V$HIF1\_Q3 |  | 158 | 0.37 | 1.65 | 0.000 | 0.049 | 1.000 | 2732 | tags=32%, list=21%, signal=40% |
| 189 | ATMPATHWAY |  | 19 | 0.57 | 1.64 | 0.013 | 0.049 | 1.000 | 1994 | tags=26%, list=15%, signal=31% |
| 190 | TAKEDA\_NUP8\_HOXA9\_10D\_DN |  | 130 | 0.39 | 1.64 | 0.000 | 0.050 | 1.000 | 3457 | tags=44%, list=26%, signal=59% |
| 191 | GCTNWTTGK\_UNKNOWN |  | 207 | 0.36 | 1.64 | 0.001 | 0.050 | 1.000 | 2788 | tags=32%, list=21%, signal=40% |
| 192 | KENNY\_WNT\_UP |  | 46 | 0.45 | 1.64 | 0.012 | 0.051 | 1.000 | 2730 | tags=50%, list=20%, signal=63% |
| 193 | STRESS\_ARSENIC\_SPECIFIC\_UP |  | 144 | 0.38 | 1.64 | 0.001 | 0.051 | 1.000 | 2481 | tags=36%, list=19%, signal=44% |
| 194 | GALINDO\_ACT\_UP |  | 73 | 0.42 | 1.64 | 0.003 | 0.051 | 1.000 | 2357 | tags=34%, list=18%, signal=41% |
| 195 | APOPTOSIS |  | 67 | 0.43 | 1.64 | 0.000 | 0.051 | 1.000 | 2645 | tags=39%, list=20%, signal=48% |
| 196 | CHR11Q23 |  | 88 | 0.40 | 1.63 | 0.003 | 0.055 | 1.000 | 3808 | tags=44%, list=29%, signal=62% |
| 197 | SMITH\_HTERT\_UP |  | 102 | 0.39 | 1.63 | 0.000 | 0.055 | 1.000 | 2727 | tags=36%, list=20%, signal=45% |
| 198 | ET743\_SARCOMA\_DN |  | 246 | 0.35 | 1.63 | 0.000 | 0.055 | 1.000 | 2653 | tags=33%, list=20%, signal=41% |
| 199 | RIBOSOMAL\_PROTEINS |  | 92 | 0.40 | 1.62 | 0.000 | 0.057 | 1.000 | 4057 | tags=41%, list=30%, signal=59% |
| 200 | CHR2P23 |  | 53 | 0.44 | 1.62 | 0.006 | 0.057 | 1.000 | 2701 | tags=40%, list=20%, signal=50% |
| 201 | IDX\_TSA\_UP\_CLUSTER4 |  | 36 | 0.48 | 1.62 | 0.009 | 0.058 | 1.000 | 2071 | tags=39%, list=16%, signal=46% |
| 202 | HBX\_HCC\_UP |  | 16 | 0.58 | 1.62 | 0.013 | 0.060 | 1.000 | 4228 | tags=75%, list=32%, signal=110% |
| 203 | V$CDPCR3\_01 |  | 33 | 0.48 | 1.62 | 0.008 | 0.060 | 1.000 | 1365 | tags=27%, list=10%, signal=30% |
| 204 | V$AHRARNT\_01 |  | 101 | 0.39 | 1.62 | 0.001 | 0.060 | 1.000 | 2891 | tags=36%, list=22%, signal=45% |
| 205 | G1PATHWAY |  | 25 | 0.52 | 1.62 | 0.016 | 0.060 | 1.000 | 2688 | tags=52%, list=20%, signal=65% |
| 206 | TTGCWCAAY\_V$CEBPB\_02 |  | 53 | 0.43 | 1.61 | 0.010 | 0.061 | 1.000 | 2670 | tags=38%, list=20%, signal=47% |
| 207 | GTTTGTT,MIR-495 |  | 201 | 0.35 | 1.61 | 0.000 | 0.062 | 1.000 | 2429 | tags=28%, list=18%, signal=34% |
| 208 | SARCOMAS\_LEIOMYOSARCOMA\_CALP\_UP |  | 15 | 0.59 | 1.61 | 0.024 | 0.062 | 1.000 | 3296 | tags=47%, list=25%, signal=62% |
| 209 | CHR5P13 |  | 30 | 0.49 | 1.61 | 0.020 | 0.063 | 1.000 | 4026 | tags=53%, list=30%, signal=76% |
| 210 | IDX\_TSA\_UP\_CLUSTER2 |  | 58 | 0.43 | 1.61 | 0.004 | 0.063 | 1.000 | 3866 | tags=55%, list=29%, signal=77% |
| 211 | RAAGNYNNCTTY\_UNKNOWN |  | 94 | 0.39 | 1.61 | 0.006 | 0.064 | 1.000 | 2681 | tags=28%, list=20%, signal=34% |
| 212 | CHR11P13 |  | 33 | 0.48 | 1.60 | 0.016 | 0.065 | 1.000 | 2046 | tags=27%, list=15%, signal=32% |
| 213 | PROTEASOME |  | 17 | 0.57 | 1.60 | 0.019 | 0.067 | 1.000 | 3924 | tags=59%, list=29%, signal=83% |
| 214 | IFN\_BETA\_GLIOMA\_UP |  | 61 | 0.43 | 1.60 | 0.009 | 0.068 | 1.000 | 3007 | tags=39%, list=23%, signal=51% |
| 215 | CROMER\_HYPOPHARYNGEAL\_MET\_VS\_NON\_DN |  | 80 | 0.40 | 1.59 | 0.004 | 0.071 | 1.000 | 2961 | tags=44%, list=22%, signal=56% |
| 216 | BASSO\_GERMINAL\_CENTER\_CD40\_UP |  | 97 | 0.39 | 1.59 | 0.005 | 0.073 | 1.000 | 3247 | tags=42%, list=24%, signal=55% |
| 217 | CHR8Q12 |  | 19 | 0.54 | 1.59 | 0.018 | 0.073 | 1.000 | 2599 | tags=58%, list=20%, signal=72% |
| 218 | CMV\_ALL\_UP |  | 90 | 0.39 | 1.59 | 0.006 | 0.073 | 1.000 | 3145 | tags=34%, list=24%, signal=45% |
| 219 | TNFALPHA\_4HRS\_UP |  | 39 | 0.46 | 1.59 | 0.015 | 0.073 | 1.000 | 3335 | tags=46%, list=25%, signal=61% |
| 220 | CTATGCA,MIR-153 |  | 171 | 0.36 | 1.59 | 0.001 | 0.072 | 1.000 | 2185 | tags=28%, list=16%, signal=33% |
| 221 | CASPASEPATHWAY |  | 22 | 0.52 | 1.59 | 0.017 | 0.074 | 1.000 | 2764 | tags=55%, list=21%, signal=69% |
| 222 | CHR12P12 |  | 43 | 0.45 | 1.58 | 0.009 | 0.076 | 1.000 | 2673 | tags=37%, list=20%, signal=46% |
| 223 | PYRIMIDINE\_METABOLISM |  | 55 | 0.42 | 1.58 | 0.013 | 0.077 | 1.000 | 3124 | tags=40%, list=23%, signal=52% |
| 224 | VHL\_NORMAL\_UP |  | 380 | 0.33 | 1.58 | 0.000 | 0.077 | 1.000 | 3736 | tags=39%, list=28%, signal=53% |
| 225 | CHR2Q14 |  | 23 | 0.52 | 1.58 | 0.022 | 0.077 | 1.000 | 3986 | tags=57%, list=30%, signal=81% |
| 226 | ET743\_SARCOMA\_72HRS\_DN |  | 202 | 0.35 | 1.58 | 0.005 | 0.076 | 1.000 | 2632 | tags=33%, list=20%, signal=41% |
| 227 | KNUDSEN\_PMNS\_UP |  | 74 | 0.40 | 1.58 | 0.007 | 0.076 | 1.000 | 3026 | tags=38%, list=23%, signal=49% |
| 228 | SANA\_IFNG\_ENDOTHELIAL\_DN |  | 80 | 0.40 | 1.58 | 0.005 | 0.076 | 1.000 | 2398 | tags=34%, list=18%, signal=41% |
| 229 | LAIRPATHWAY |  | 15 | 0.58 | 1.58 | 0.025 | 0.077 | 1.000 | 3211 | tags=47%, list=24%, signal=61% |
| 230 | MOREAUX\_TACI\_HI\_IN\_PPC\_UP |  | 56 | 0.43 | 1.58 | 0.009 | 0.076 | 1.000 | 2478 | tags=39%, list=19%, signal=48% |
| 231 | PENG\_GLUCOSE\_DN |  | 133 | 0.37 | 1.58 | 0.001 | 0.076 | 1.000 | 3484 | tags=40%, list=26%, signal=53% |
| 232 | GOLDRATH\_CYTOLYTIC |  | 17 | 0.55 | 1.57 | 0.041 | 0.081 | 1.000 | 4149 | tags=65%, list=31%, signal=94% |
| 233 | HIF1\_TARGETS |  | 35 | 0.46 | 1.57 | 0.013 | 0.082 | 1.000 | 2043 | tags=43%, list=15%, signal=50% |
| 234 | CHR7P15 |  | 47 | 0.43 | 1.57 | 0.013 | 0.084 | 1.000 | 4938 | tags=57%, list=37%, signal=91% |
| 235 | V$GNCF\_01 |  | 51 | 0.42 | 1.56 | 0.013 | 0.086 | 1.000 | 1486 | tags=31%, list=11%, signal=35% |
| 236 | ROME\_INSULIN\_2F\_DN |  | 24 | 0.49 | 1.56 | 0.019 | 0.086 | 1.000 | 3960 | tags=50%, list=30%, signal=71% |
| 237 | CHR7Q31 |  | 62 | 0.41 | 1.56 | 0.009 | 0.086 | 1.000 | 5033 | tags=53%, list=38%, signal=85% |
| 238 | UVC\_TTD\_4HR\_DN |  | 297 | 0.33 | 1.56 | 0.000 | 0.086 | 1.000 | 2467 | tags=28%, list=19%, signal=34% |
| 239 | DSRNA\_UP |  | 37 | 0.46 | 1.56 | 0.016 | 0.086 | 1.000 | 2662 | tags=43%, list=20%, signal=54% |
| 240 | CHR3Q21 |  | 69 | 0.40 | 1.56 | 0.011 | 0.086 | 1.000 | 3134 | tags=39%, list=24%, signal=51% |
| 241 | CHR3Q27 |  | 38 | 0.45 | 1.56 | 0.021 | 0.087 | 1.000 | 3602 | tags=50%, list=27%, signal=68% |
| 242 | KLEIN\_PEL\_DN |  | 58 | 0.41 | 1.56 | 0.009 | 0.087 | 1.000 | 1941 | tags=28%, list=15%, signal=32% |
| 243 | YRTCANNRCGC\_UNKNOWN |  | 43 | 0.44 | 1.56 | 0.020 | 0.087 | 1.000 | 3406 | tags=40%, list=26%, signal=53% |
| 244 | CELL\_CYCLE\_CHECKPOINT |  | 24 | 0.50 | 1.55 | 0.035 | 0.090 | 1.000 | 2380 | tags=42%, list=18%, signal=51% |
| 245 | HYPOXIA\_REVIEW |  | 79 | 0.39 | 1.55 | 0.007 | 0.090 | 1.000 | 2343 | tags=38%, list=18%, signal=46% |
| 246 | GTAAGAT,MIR-200A |  | 45 | 0.43 | 1.55 | 0.013 | 0.090 | 1.000 | 2789 | tags=33%, list=21%, signal=42% |
| 247 | CTLA4PATHWAY |  | 18 | 0.54 | 1.54 | 0.030 | 0.095 | 1.000 | 4563 | tags=61%, list=34%, signal=93% |
| 248 | CHRXP22 |  | 91 | 0.38 | 1.54 | 0.010 | 0.095 | 1.000 | 2473 | tags=32%, list=19%, signal=39% |
| 249 | AGCATTA,MIR-155 |  | 104 | 0.37 | 1.54 | 0.003 | 0.099 | 1.000 | 3048 | tags=35%, list=23%, signal=45% |
| 250 | SHEPARD\_CELL\_PROLIFERATION |  | 198 | 0.34 | 1.54 | 0.000 | 0.098 | 1.000 | 2414 | tags=29%, list=18%, signal=35% |
| 251 | V$IRF7\_01 |  | 174 | 0.35 | 1.54 | 0.003 | 0.099 | 1.000 | 3128 | tags=33%, list=23%, signal=43% |
| 252 | HSC\_LTHSC\_ADULT |  | 286 | 0.33 | 1.54 | 0.000 | 0.100 | 1.000 | 2746 | tags=32%, list=21%, signal=39% |
| 253 | V$MYCMAX\_01 |  | 189 | 0.34 | 1.54 | 0.000 | 0.100 | 1.000 | 2647 | tags=30%, list=20%, signal=36% |
| 254 | ST\_B\_CELL\_ANTIGEN\_RECEPTOR |  | 39 | 0.44 | 1.54 | 0.014 | 0.100 | 1.000 | 3460 | tags=49%, list=26%, signal=66% |
| 255 | CELL\_PROLIFERATION |  | 198 | 0.34 | 1.54 | 0.000 | 0.100 | 1.000 | 2414 | tags=29%, list=18%, signal=35% |
| 256 | PROTEASOME\_DEGRADATION |  | 31 | 0.46 | 1.54 | 0.024 | 0.100 | 1.000 | 3727 | tags=52%, list=28%, signal=71% |
| 257 | WONG\_IFNA\_HCC\_RESISTANT\_VS\_SENSITIVE\_DN |  | 30 | 0.47 | 1.53 | 0.027 | 0.100 | 1.000 | 1970 | tags=33%, list=15%, signal=39% |
| 258 | ST\_G\_ALPHA\_I\_PATHWAY |  | 34 | 0.46 | 1.53 | 0.018 | 0.103 | 1.000 | 3852 | tags=53%, list=29%, signal=74% |
| 259 | HOHENKIRK\_MONOCYTE\_DEND\_DN |  | 116 | 0.36 | 1.53 | 0.004 | 0.103 | 1.000 | 3873 | tags=47%, list=29%, signal=65% |
| 260 | ST\_T\_CELL\_SIGNAL\_TRANSDUCTION |  | 42 | 0.44 | 1.53 | 0.019 | 0.104 | 1.000 | 4715 | tags=55%, list=35%, signal=84% |
| 261 | YE\_INTRAMETASTATIC\_HCC\_UP |  | 21 | 0.51 | 1.53 | 0.047 | 0.106 | 1.000 | 2346 | tags=38%, list=18%, signal=46% |
| 262 | CHR11P14 |  | 15 | 0.56 | 1.53 | 0.044 | 0.106 | 1.000 | 3133 | tags=47%, list=24%, signal=61% |
| 263 | YAGI\_AML\_PROGNOSIS |  | 34 | 0.46 | 1.52 | 0.023 | 0.107 | 1.000 | 2713 | tags=44%, list=20%, signal=55% |
| 264 | AGEING\_KIDNEY\_UP |  | 329 | 0.32 | 1.52 | 0.000 | 0.109 | 1.000 | 2996 | tags=35%, list=22%, signal=44% |
| 265 | CELL\_CYCLE\_REGULATOR |  | 24 | 0.48 | 1.52 | 0.038 | 0.108 | 1.000 | 2211 | tags=42%, list=17%, signal=50% |
| 266 | CHR12P11 |  | 23 | 0.50 | 1.52 | 0.028 | 0.108 | 1.000 | 5286 | tags=70%, list=40%, signal=115% |
| 267 | CHR8Q11 |  | 17 | 0.53 | 1.52 | 0.053 | 0.108 | 1.000 | 2470 | tags=35%, list=19%, signal=43% |
| 268 | LEE\_TCELLS1\_UP |  | 136 | 0.35 | 1.52 | 0.001 | 0.109 | 1.000 | 1455 | tags=24%, list=11%, signal=26% |
| 269 | DNMT1\_KO\_DN |  | 15 | 0.55 | 1.52 | 0.049 | 0.109 | 1.000 | 2844 | tags=53%, list=21%, signal=68% |
| 270 | TNFALPHA\_ALL\_UP |  | 74 | 0.39 | 1.52 | 0.007 | 0.108 | 1.000 | 2581 | tags=34%, list=19%, signal=42% |
| 271 | PROTEASOMEPATHWAY |  | 21 | 0.52 | 1.52 | 0.038 | 0.109 | 1.000 | 3994 | tags=52%, list=30%, signal=75% |
| 272 | HDACI\_COLON\_BUT48HRS\_DN |  | 99 | 0.36 | 1.52 | 0.008 | 0.110 | 1.000 | 2192 | tags=33%, list=16%, signal=40% |
| 273 | V$NRSF\_01 |  | 69 | 0.39 | 1.52 | 0.016 | 0.109 | 1.000 | 5787 | tags=57%, list=43%, signal=99% |
| 274 | SIG\_PIP3\_SIGNALING\_IN\_B\_LYMPHOCYTES |  | 34 | 0.45 | 1.52 | 0.030 | 0.109 | 1.000 | 4563 | tags=53%, list=34%, signal=80% |
| 275 | IFN\_BETA\_UP |  | 65 | 0.40 | 1.52 | 0.013 | 0.110 | 1.000 | 3640 | tags=45%, list=27%, signal=61% |
| 276 | CHR9P21 |  | 20 | 0.52 | 1.52 | 0.052 | 0.109 | 1.000 | 2907 | tags=40%, list=22%, signal=51% |
| 277 | TNF\_AND\_FAS\_NETWORK |  | 16 | 0.54 | 1.52 | 0.043 | 0.110 | 1.000 | 2530 | tags=50%, list=19%, signal=62% |
| 278 | CELL\_MOTILITY |  | 101 | 0.37 | 1.52 | 0.007 | 0.110 | 1.000 | 2395 | tags=35%, list=18%, signal=42% |
| 279 | BRCA1\_MES\_UP |  | 38 | 0.44 | 1.51 | 0.019 | 0.111 | 1.000 | 2722 | tags=37%, list=20%, signal=46% |
| 280 | CMV\_HCMV\_TIMECOURSE\_48HRS\_UP |  | 66 | 0.39 | 1.51 | 0.007 | 0.113 | 1.000 | 2094 | tags=29%, list=16%, signal=34% |
| 281 | V$HIF1\_Q5 |  | 164 | 0.35 | 1.51 | 0.003 | 0.113 | 1.000 | 3806 | tags=41%, list=29%, signal=56% |
| 282 | UVB\_NHEK1\_DN |  | 270 | 0.33 | 1.51 | 0.000 | 0.113 | 1.000 | 1954 | tags=24%, list=15%, signal=28% |
| 283 | LEE\_TCELLS8\_UP |  | 136 | 0.35 | 1.51 | 0.007 | 0.113 | 1.000 | 1455 | tags=24%, list=11%, signal=26% |
| 284 | UVC\_XPCS\_4HR\_DN |  | 242 | 0.33 | 1.51 | 0.002 | 0.114 | 1.000 | 2217 | tags=24%, list=17%, signal=28% |
| 285 | UVC\_XPCS\_ALL\_DN |  | 478 | 0.31 | 1.51 | 0.000 | 0.114 | 1.000 | 2258 | tags=24%, list=17%, signal=28% |
| 286 | CMV\_HCMV\_TIMECOURSE\_ALL\_UP |  | 462 | 0.31 | 1.51 | 0.000 | 0.114 | 1.000 | 3118 | tags=32%, list=23%, signal=41% |
| 287 | AGUIRRE\_PANCREAS\_CHR18 |  | 17 | 0.53 | 1.51 | 0.052 | 0.114 | 1.000 | 1951 | tags=35%, list=15%, signal=41% |
| 288 | LEE\_TCELLS10\_UP |  | 136 | 0.35 | 1.51 | 0.005 | 0.114 | 1.000 | 1455 | tags=24%, list=11%, signal=26% |
| 289 | V$SOX9\_B1 |  | 173 | 0.34 | 1.51 | 0.004 | 0.114 | 1.000 | 3441 | tags=36%, list=26%, signal=48% |
| 290 | GOLUB\_ALL\_VS\_AML\_UP |  | 19 | 0.51 | 1.51 | 0.040 | 0.114 | 1.000 | 3145 | tags=47%, list=24%, signal=62% |
| 291 | GTATTAT,MIR-369-3P |  | 152 | 0.35 | 1.51 | 0.003 | 0.114 | 1.000 | 3025 | tags=32%, list=23%, signal=40% |
| 292 | IDX\_TSA\_UP\_CLUSTER1 |  | 23 | 0.49 | 1.51 | 0.031 | 0.114 | 1.000 | 1081 | tags=30%, list=8%, signal=33% |
| 293 | V$HP1SITEFACTOR\_Q6 |  | 164 | 0.34 | 1.50 | 0.005 | 0.114 | 1.000 | 2253 | tags=26%, list=17%, signal=30% |
| 294 | GH\_GHRHR\_KO\_6HRS\_UP |  | 62 | 0.40 | 1.50 | 0.023 | 0.114 | 1.000 | 3872 | tags=44%, list=29%, signal=61% |
| 295 | TACTTGA,MIR-26A,MIR-26B |  | 216 | 0.33 | 1.50 | 0.003 | 0.114 | 1.000 | 2506 | tags=28%, list=19%, signal=34% |
| 296 | ASTON\_OLIGODENDROGLIA\_MYELINATION\_SUBSET |  | 17 | 0.53 | 1.50 | 0.054 | 0.116 | 1.000 | 3074 | tags=53%, list=23%, signal=69% |
| 297 | BRENTANI\_SIGNALING |  | 174 | 0.34 | 1.50 | 0.003 | 0.119 | 1.000 | 3076 | tags=33%, list=23%, signal=43% |
| 298 | RHOPATHWAY |  | 29 | 0.46 | 1.50 | 0.032 | 0.120 | 1.000 | 2952 | tags=41%, list=22%, signal=53% |
| 299 | BLEO\_MOUSE\_LYMPH\_LOW\_24HRS\_DN |  | 24 | 0.48 | 1.49 | 0.040 | 0.121 | 1.000 | 2921 | tags=42%, list=22%, signal=53% |
| 300 | CELL\_CYCLE\_ARREST |  | 31 | 0.45 | 1.49 | 0.033 | 0.123 | 1.000 | 3739 | tags=52%, list=28%, signal=72% |
| 301 | HOFMANN\_MANTEL\_LYMPHOMA\_VS\_LYMPH\_NODES\_UP |  | 48 | 0.42 | 1.49 | 0.023 | 0.123 | 1.000 | 3105 | tags=42%, list=23%, signal=54% |
| 302 | IL12PATHWAY |  | 20 | 0.50 | 1.49 | 0.037 | 0.123 | 1.000 | 2309 | tags=35%, list=17%, signal=42% |
| 303 | O6BG\_RESIST\_MEDULLOBLASTOMA\_DN |  | 51 | 0.41 | 1.49 | 0.029 | 0.123 | 1.000 | 3429 | tags=41%, list=26%, signal=55% |
| 304 | CHR16Q13 |  | 31 | 0.46 | 1.49 | 0.034 | 0.123 | 1.000 | 3709 | tags=52%, list=28%, signal=71% |
| 305 | TPA\_RESIST\_MIDDLE\_DN |  | 107 | 0.36 | 1.49 | 0.017 | 0.125 | 1.000 | 3397 | tags=36%, list=26%, signal=49% |
| 306 | AGTCTTA,MIR-499 |  | 56 | 0.40 | 1.48 | 0.019 | 0.129 | 1.000 | 2722 | tags=36%, list=20%, signal=45% |
| 307 | PROLIFERATION\_GENES |  | 362 | 0.31 | 1.48 | 0.002 | 0.129 | 1.000 | 2811 | tags=27%, list=21%, signal=33% |
| 308 | CHR7Q34 |  | 22 | 0.49 | 1.48 | 0.046 | 0.134 | 1.000 | 4346 | tags=59%, list=33%, signal=88% |
| 309 | MAGRANGEAS\_MULTIPLE\_MYELOMA\_IGL\_VS\_IGK\_DN |  | 27 | 0.46 | 1.48 | 0.040 | 0.133 | 1.000 | 3442 | tags=59%, list=26%, signal=80% |
| 310 | TPA\_SENS\_MIDDLE\_DN |  | 299 | 0.31 | 1.48 | 0.001 | 0.136 | 1.000 | 1973 | tags=24%, list=15%, signal=28% |
| 311 | HIPPOCAMPUS\_DEVELOPMENT\_PRENATAL |  | 32 | 0.44 | 1.48 | 0.053 | 0.136 | 1.000 | 2709 | tags=38%, list=20%, signal=47% |
| 312 | HYPOXIA\_RCC\_UP |  | 86 | 0.36 | 1.48 | 0.023 | 0.137 | 1.000 | 2256 | tags=27%, list=17%, signal=32% |
| 313 | GLYCOLYSIS\_AND\_GLUCONEOGENESIS |  | 42 | 0.42 | 1.47 | 0.026 | 0.137 | 1.000 | 1763 | tags=31%, list=13%, signal=36% |
| 314 | BRCA1\_SW480\_DN |  | 16 | 0.53 | 1.47 | 0.058 | 0.136 | 1.000 | 1973 | tags=38%, list=15%, signal=44% |
| 315 | UVB\_NHEK1\_C6 |  | 130 | 0.35 | 1.47 | 0.011 | 0.137 | 1.000 | 1805 | tags=23%, list=14%, signal=26% |
| 316 | P53\_BRCA1\_UP |  | 28 | 0.46 | 1.47 | 0.063 | 0.137 | 1.000 | 1651 | tags=32%, list=12%, signal=37% |
| 317 | ZHAN\_MULTIPLE\_MYELOMA\_VS\_NORMAL\_DN |  | 38 | 0.43 | 1.47 | 0.029 | 0.138 | 1.000 | 4085 | tags=50%, list=31%, signal=72% |
| 318 | DAC\_PANC\_UP |  | 363 | 0.31 | 1.47 | 0.001 | 0.138 | 1.000 | 3355 | tags=34%, list=25%, signal=44% |
| 319 | ET743\_SARCOMA\_48HRS\_DN |  | 169 | 0.33 | 1.47 | 0.005 | 0.137 | 1.000 | 2591 | tags=34%, list=19%, signal=41% |
| 320 | AGGGCCA,MIR-328 |  | 59 | 0.39 | 1.47 | 0.025 | 0.138 | 1.000 | 4053 | tags=47%, list=30%, signal=68% |
| 321 | TSA\_CD4\_DN |  | 18 | 0.51 | 1.47 | 0.069 | 0.139 | 1.000 | 1188 | tags=28%, list=9%, signal=30% |
| 322 | MANALO\_HYPOXIA\_UP |  | 92 | 0.36 | 1.47 | 0.013 | 0.139 | 1.000 | 2653 | tags=35%, list=20%, signal=43% |
| 323 | CHR15Q24 |  | 53 | 0.40 | 1.47 | 0.041 | 0.139 | 1.000 | 3178 | tags=38%, list=24%, signal=49% |
| 324 | IFN\_ANY\_UP |  | 81 | 0.37 | 1.47 | 0.018 | 0.139 | 1.000 | 3640 | tags=42%, list=27%, signal=57% |
| 325 | CCGNMNNTNACG\_UNKNOWN |  | 53 | 0.40 | 1.47 | 0.029 | 0.138 | 1.000 | 1696 | tags=28%, list=13%, signal=32% |
| 326 | BRENTANI\_PROTEIN\_MODIFICATION |  | 142 | 0.34 | 1.47 | 0.011 | 0.138 | 1.000 | 3442 | tags=37%, list=26%, signal=50% |
| 327 | IRS1\_KO\_ADIP\_UP |  | 84 | 0.37 | 1.47 | 0.026 | 0.139 | 1.000 | 2679 | tags=35%, list=20%, signal=43% |
| 328 | INFLAMPATHWAY |  | 29 | 0.45 | 1.47 | 0.055 | 0.139 | 1.000 | 3818 | tags=41%, list=29%, signal=58% |
| 329 | BCRABL\_HL60\_CDNA\_DN |  | 28 | 0.45 | 1.47 | 0.043 | 0.139 | 1.000 | 2641 | tags=46%, list=20%, signal=58% |
| 330 | TSADAC\_RKOEXP\_UP |  | 15 | 0.53 | 1.47 | 0.051 | 0.139 | 1.000 | 1638 | tags=40%, list=12%, signal=46% |
| 331 | UVC\_XPCS\_8HR\_DN |  | 408 | 0.30 | 1.46 | 0.001 | 0.140 | 1.000 | 2258 | tags=24%, list=17%, signal=28% |
| 332 | IRITANI\_ADPROX\_DN |  | 60 | 0.39 | 1.46 | 0.029 | 0.140 | 1.000 | 2624 | tags=35%, list=20%, signal=43% |
| 333 | PROSTAGLANDIN\_SYNTHESIS\_REGULATION |  | 28 | 0.46 | 1.46 | 0.038 | 0.141 | 1.000 | 4026 | tags=57%, list=30%, signal=82% |
| 334 | GENOTOXINS\_ALL\_24HRS\_REG |  | 28 | 0.46 | 1.46 | 0.055 | 0.141 | 1.000 | 2645 | tags=39%, list=20%, signal=49% |
| 335 | V$ATF4\_Q2 |  | 172 | 0.33 | 1.46 | 0.009 | 0.142 | 1.000 | 4128 | tags=42%, list=31%, signal=61% |
| 336 | CHR3Q26 |  | 40 | 0.42 | 1.46 | 0.049 | 0.142 | 1.000 | 2826 | tags=35%, list=21%, signal=44% |
| 337 | ACTGTAG,MIR-139 |  | 95 | 0.36 | 1.46 | 0.020 | 0.142 | 1.000 | 1909 | tags=25%, list=14%, signal=29% |
| 338 | DER\_IFNG\_UP |  | 61 | 0.39 | 1.46 | 0.030 | 0.143 | 1.000 | 3184 | tags=41%, list=24%, signal=54% |
| 339 | P53\_SIGNALING |  | 91 | 0.36 | 1.46 | 0.014 | 0.143 | 1.000 | 2611 | tags=30%, list=20%, signal=37% |
| 340 | CHR1P32 |  | 53 | 0.40 | 1.46 | 0.031 | 0.143 | 1.000 | 1484 | tags=26%, list=11%, signal=30% |
| 341 | V$NFKAPPAB\_01 |  | 184 | 0.33 | 1.46 | 0.010 | 0.144 | 1.000 | 3547 | tags=37%, list=27%, signal=50% |
| 342 | V$LEF1\_Q6 |  | 184 | 0.33 | 1.46 | 0.008 | 0.145 | 1.000 | 3259 | tags=35%, list=24%, signal=46% |
| 343 | UVB\_NHEK3\_ALL |  | 390 | 0.30 | 1.46 | 0.000 | 0.144 | 1.000 | 2520 | tags=29%, list=19%, signal=35% |
| 344 | HUMAN\_CD34\_ENRICHED\_TRANSCRIPTION\_FACTORS |  | 179 | 0.33 | 1.46 | 0.008 | 0.145 | 1.000 | 3612 | tags=37%, list=27%, signal=51% |
| 345 | GTGCCAT,MIR-183 |  | 131 | 0.34 | 1.46 | 0.015 | 0.144 | 1.000 | 3162 | tags=38%, list=24%, signal=50% |
| 346 | HDACI\_COLON\_BUT16HRS\_DN |  | 97 | 0.36 | 1.46 | 0.017 | 0.144 | 1.000 | 2716 | tags=37%, list=20%, signal=46% |
| 347 | CMV\_HCMV\_TIMECOURSE\_16HRS\_UP |  | 56 | 0.39 | 1.46 | 0.028 | 0.144 | 1.000 | 1984 | tags=30%, list=15%, signal=36% |
| 348 | HDACI\_COLON\_BUT\_DN |  | 214 | 0.32 | 1.45 | 0.003 | 0.144 | 1.000 | 2753 | tags=33%, list=21%, signal=41% |
| 349 | TTTGCAG,MIR-518A-2 |  | 157 | 0.33 | 1.45 | 0.005 | 0.145 | 1.000 | 2950 | tags=31%, list=22%, signal=40% |
| 350 | CHR10P15 |  | 31 | 0.44 | 1.45 | 0.051 | 0.145 | 1.000 | 3301 | tags=48%, list=25%, signal=64% |
| 351 | KANNAN\_P53\_DN |  | 15 | 0.54 | 1.45 | 0.057 | 0.145 | 1.000 | 2365 | tags=40%, list=18%, signal=49% |
| 352 | XU\_ATRA\_UP |  | 15 | 0.53 | 1.45 | 0.060 | 0.146 | 1.000 | 1445 | tags=33%, list=11%, signal=37% |
| 353 | LEI\_MYB\_REGULATED\_GENES |  | 317 | 0.31 | 1.45 | 0.001 | 0.145 | 1.000 | 1444 | tags=22%, list=11%, signal=24% |
| 354 | V$TCF1P\_Q6 |  | 169 | 0.33 | 1.45 | 0.008 | 0.147 | 1.000 | 4115 | tags=43%, list=31%, signal=61% |
| 355 | DER\_IFNB\_UP |  | 91 | 0.36 | 1.45 | 0.016 | 0.147 | 1.000 | 3184 | tags=38%, list=24%, signal=50% |
| 356 | TPA\_SENS\_LATE\_DN |  | 232 | 0.32 | 1.45 | 0.001 | 0.147 | 1.000 | 2559 | tags=28%, list=19%, signal=35% |
| 357 | IRITANI\_ADPROX\_VASC |  | 151 | 0.33 | 1.45 | 0.017 | 0.151 | 1.000 | 2654 | tags=31%, list=20%, signal=38% |
| 358 | RUTELLA\_HEMATOGFSNDCS\_DIFF |  | 661 | 0.29 | 1.45 | 0.000 | 0.150 | 1.000 | 3510 | tags=38%, list=26%, signal=48% |
| 359 | CHR15Q25 |  | 31 | 0.43 | 1.44 | 0.061 | 0.155 | 1.000 | 2184 | tags=32%, list=16%, signal=38% |
| 360 | UVC\_HIGH\_D3\_DN |  | 46 | 0.40 | 1.44 | 0.032 | 0.154 | 1.000 | 1107 | tags=30%, list=8%, signal=33% |
| 361 | CTACTGT,MIR-199A |  | 131 | 0.34 | 1.44 | 0.008 | 0.156 | 1.000 | 3096 | tags=34%, list=23%, signal=43% |
| 362 | CHR2P11 |  | 32 | 0.43 | 1.44 | 0.048 | 0.156 | 1.000 | 1333 | tags=31%, list=10%, signal=35% |
| 363 | MARSHALL\_SPLEEN\_BAL |  | 20 | 0.49 | 1.44 | 0.078 | 0.156 | 1.000 | 3546 | tags=60%, list=27%, signal=82% |
| 364 | HOFFMANN\_BIVSBII\_LGBII |  | 90 | 0.36 | 1.44 | 0.021 | 0.157 | 1.000 | 3145 | tags=43%, list=24%, signal=56% |
| 365 | JNK\_DN |  | 31 | 0.43 | 1.44 | 0.062 | 0.157 | 1.000 | 2229 | tags=42%, list=17%, signal=50% |
| 366 | CHR11Q24 |  | 28 | 0.44 | 1.44 | 0.063 | 0.157 | 1.000 | 4133 | tags=39%, list=31%, signal=57% |
| 367 | V$E2F1\_Q4 |  | 175 | 0.32 | 1.44 | 0.010 | 0.157 | 1.000 | 3259 | tags=33%, list=24%, signal=43% |
| 368 | ZHAN\_MMPC\_SIM |  | 42 | 0.41 | 1.44 | 0.040 | 0.158 | 1.000 | 3028 | tags=43%, list=23%, signal=55% |
| 369 | YANG\_OSTECLASTS\_SIG |  | 38 | 0.42 | 1.44 | 0.047 | 0.159 | 1.000 | 3899 | tags=50%, list=29%, signal=70% |
| 370 | CHR9P24 |  | 28 | 0.45 | 1.44 | 0.069 | 0.159 | 1.000 | 3096 | tags=39%, list=23%, signal=51% |
| 371 | TACGGGT,MIR-99A,MIR-100,MIR-99B |  | 19 | 0.49 | 1.44 | 0.056 | 0.158 | 1.000 | 2502 | tags=32%, list=19%, signal=39% |
| 372 | TSADAC\_PANC50\_UP |  | 42 | 0.41 | 1.43 | 0.042 | 0.158 | 1.000 | 2744 | tags=36%, list=21%, signal=45% |
| 373 | ZHAN\_TONSIL\_PCBC |  | 43 | 0.40 | 1.43 | 0.032 | 0.160 | 1.000 | 2764 | tags=44%, list=21%, signal=56% |
| 374 | CHR2Q12 |  | 26 | 0.46 | 1.43 | 0.062 | 0.160 | 1.000 | 4413 | tags=54%, list=33%, signal=80% |
| 375 | CORDERO\_KRAS\_KD\_VS\_CONTROL\_DN |  | 55 | 0.39 | 1.43 | 0.043 | 0.160 | 1.000 | 2058 | tags=29%, list=15%, signal=34% |
| 376 | BRENTANI\_REPAIR |  | 35 | 0.42 | 1.43 | 0.048 | 0.161 | 1.000 | 1714 | tags=31%, list=13%, signal=36% |
| 377 | TGCCTTA,MIR-124A |  | 418 | 0.29 | 1.43 | 0.001 | 0.161 | 1.000 | 3023 | tags=32%, list=23%, signal=40% |
| 378 | BRENTANI\_CELL\_ADHESION |  | 91 | 0.35 | 1.43 | 0.014 | 0.162 | 1.000 | 3638 | tags=40%, list=27%, signal=54% |
| 379 | DNMT1\_KO\_UP |  | 70 | 0.37 | 1.43 | 0.024 | 0.161 | 1.000 | 3857 | tags=47%, list=29%, signal=66% |
| 380 | CHRXQ28 |  | 76 | 0.37 | 1.43 | 0.041 | 0.161 | 1.000 | 2950 | tags=39%, list=22%, signal=50% |
| 381 | TSA\_HEPATOMA\_CANCER\_DN |  | 16 | 0.52 | 1.43 | 0.067 | 0.161 | 1.000 | 2217 | tags=38%, list=17%, signal=45% |
| 382 | ACAACCT,MIR-453 |  | 37 | 0.42 | 1.43 | 0.056 | 0.162 | 1.000 | 3276 | tags=38%, list=25%, signal=50% |
| 383 | MYOD\_BRG1\_UP |  | 27 | 0.44 | 1.43 | 0.058 | 0.162 | 1.000 | 2958 | tags=33%, list=22%, signal=43% |
| 384 | LAL\_KO\_6MO\_UP |  | 56 | 0.38 | 1.43 | 0.038 | 0.162 | 1.000 | 2352 | tags=30%, list=18%, signal=37% |
| 385 | UVC\_TTD\_ALL\_DN |  | 358 | 0.30 | 1.43 | 0.000 | 0.162 | 1.000 | 2237 | tags=25%, list=17%, signal=29% |
| 386 | IFN\_GAMMA\_UP |  | 38 | 0.41 | 1.43 | 0.048 | 0.162 | 1.000 | 4056 | tags=47%, list=30%, signal=68% |
| 387 | TGGTGCT,MIR-29A,MIR-29B,MIR-29C |  | 370 | 0.30 | 1.43 | 0.003 | 0.163 | 1.000 | 3687 | tags=36%, list=28%, signal=48% |
| 388 | MOREAUX\_TACI\_HI\_VS\_LOW\_DN |  | 143 | 0.33 | 1.42 | 0.016 | 0.164 | 1.000 | 2722 | tags=32%, list=20%, signal=40% |
| 389 | TGASTMAGC\_V$NFE2\_01 |  | 147 | 0.33 | 1.42 | 0.009 | 0.166 | 1.000 | 3672 | tags=38%, list=28%, signal=52% |
| 390 | CHR18P11 |  | 51 | 0.39 | 1.42 | 0.044 | 0.166 | 1.000 | 4223 | tags=49%, list=32%, signal=71% |
| 391 | ZUCCHI\_EPITHELIAL\_UP |  | 41 | 0.40 | 1.42 | 0.052 | 0.166 | 1.000 | 2647 | tags=27%, list=20%, signal=33% |
| 392 | OXSTRESS\_RPETHREE\_DN |  | 28 | 0.45 | 1.42 | 0.052 | 0.166 | 1.000 | 1260 | tags=32%, list=9%, signal=35% |
| 393 | ST\_INTEGRIN\_SIGNALING\_PATHWAY |  | 75 | 0.36 | 1.42 | 0.033 | 0.170 | 1.000 | 3460 | tags=36%, list=26%, signal=48% |
| 394 | V$NFY\_Q6 |  | 181 | 0.32 | 1.42 | 0.016 | 0.171 | 1.000 | 3774 | tags=35%, list=28%, signal=49% |
| 395 | HDACI\_COLON\_SUL30MIN\_DN |  | 32 | 0.43 | 1.42 | 0.049 | 0.170 | 1.000 | 3784 | tags=44%, list=28%, signal=61% |
| 396 | ELONGINA\_KO\_UP |  | 146 | 0.33 | 1.42 | 0.018 | 0.170 | 1.000 | 1893 | tags=25%, list=14%, signal=28% |
| 397 | XU\_CBP\_UP |  | 19 | 0.49 | 1.42 | 0.073 | 0.170 | 1.000 | 2291 | tags=53%, list=17%, signal=63% |
| 398 | UVC\_HIGH\_ALL\_DN |  | 295 | 0.30 | 1.42 | 0.002 | 0.170 | 1.000 | 2571 | tags=30%, list=19%, signal=36% |
| 399 | BRG1\_ALAB\_UP |  | 37 | 0.41 | 1.42 | 0.051 | 0.171 | 1.000 | 3110 | tags=43%, list=23%, signal=56% |
| 400 | BLEO\_HUMAN\_LYMPH\_HIGH\_24HRS\_UP |  | 92 | 0.34 | 1.41 | 0.035 | 0.175 | 1.000 | 2654 | tags=34%, list=20%, signal=42% |
| 401 | ST\_GAQ\_PATHWAY |  | 27 | 0.44 | 1.41 | 0.066 | 0.175 | 1.000 | 3852 | tags=44%, list=29%, signal=62% |
| 402 | RRCCGTTA\_UNKNOWN |  | 60 | 0.37 | 1.41 | 0.047 | 0.179 | 1.000 | 1500 | tags=23%, list=11%, signal=26% |
| 403 | TTCNRGNNNNTTC\_V$HSF\_Q6 |  | 110 | 0.34 | 1.41 | 0.025 | 0.179 | 1.000 | 2851 | tags=32%, list=21%, signal=40% |
| 404 | V$GATA1\_01 |  | 172 | 0.32 | 1.41 | 0.015 | 0.179 | 1.000 | 3245 | tags=34%, list=24%, signal=44% |
| 405 | V$PAX2\_01 |  | 37 | 0.41 | 1.41 | 0.056 | 0.180 | 1.000 | 2881 | tags=46%, list=22%, signal=58% |
| 406 | TPA\_SENS\_LATE\_UP |  | 52 | 0.39 | 1.41 | 0.044 | 0.181 | 1.000 | 3709 | tags=44%, list=28%, signal=61% |
| 407 | ST\_FAS\_SIGNALING\_PATHWAY |  | 59 | 0.37 | 1.41 | 0.034 | 0.181 | 1.000 | 3546 | tags=41%, list=27%, signal=55% |
| 408 | ZHAN\_MMPC\_EARLYVS |  | 48 | 0.39 | 1.41 | 0.040 | 0.181 | 1.000 | 2764 | tags=42%, list=21%, signal=52% |
| 409 | UV\_UNIQUE\_FIBRO\_DN |  | 30 | 0.43 | 1.40 | 0.065 | 0.182 | 1.000 | 1694 | tags=27%, list=13%, signal=30% |
| 410 | PASSERINI\_APOPTOSIS |  | 42 | 0.40 | 1.40 | 0.061 | 0.183 | 1.000 | 3483 | tags=43%, list=26%, signal=58% |
| 411 | V$CEBP\_C |  | 149 | 0.32 | 1.40 | 0.021 | 0.183 | 1.000 | 2215 | tags=25%, list=17%, signal=29% |
| 412 | HYPOXIA\_FIBRO\_UP |  | 20 | 0.48 | 1.40 | 0.073 | 0.184 | 1.000 | 3911 | tags=60%, list=29%, signal=85% |
| 413 | ATTCTTT,MIR-186 |  | 206 | 0.31 | 1.40 | 0.004 | 0.185 | 1.000 | 3494 | tags=34%, list=26%, signal=46% |
| 414 | CELLCYCLEPATHWAY |  | 22 | 0.46 | 1.40 | 0.090 | 0.185 | 1.000 | 3148 | tags=64%, list=24%, signal=83% |
| 415 | FLECHNER\_KIDNEY\_TRANSPLANT\_REJECTION\_PBL\_DN |  | 51 | 0.38 | 1.40 | 0.051 | 0.187 | 1.000 | 2074 | tags=31%, list=16%, signal=37% |
| 416 | VANASSE\_BCL2\_TARGETS |  | 76 | 0.35 | 1.40 | 0.047 | 0.187 | 1.000 | 1748 | tags=25%, list=13%, signal=29% |
| 417 | RUTELLA\_HEPATGFSNDCS\_UP |  | 158 | 0.32 | 1.40 | 0.025 | 0.189 | 1.000 | 3457 | tags=38%, list=26%, signal=51% |
| 418 | TPA\_SENS\_EARLY\_DN |  | 283 | 0.30 | 1.40 | 0.005 | 0.190 | 1.000 | 2270 | tags=25%, list=17%, signal=30% |
| 419 | CHR15Q26 |  | 41 | 0.40 | 1.40 | 0.061 | 0.189 | 1.000 | 2429 | tags=32%, list=18%, signal=39% |
| 420 | GAMMA\_UNIQUE\_FIBRO\_DN |  | 56 | 0.37 | 1.39 | 0.039 | 0.190 | 1.000 | 3066 | tags=34%, list=23%, signal=44% |
| 421 | STEMCELL\_COMMON\_DN |  | 55 | 0.37 | 1.39 | 0.063 | 0.191 | 1.000 | 3634 | tags=53%, list=27%, signal=72% |
| 422 | PURINE\_METABOLISM |  | 111 | 0.34 | 1.39 | 0.032 | 0.192 | 1.000 | 2407 | tags=27%, list=18%, signal=33% |
| 423 | V$USF2\_Q6 |  | 175 | 0.31 | 1.39 | 0.016 | 0.191 | 1.000 | 2628 | tags=29%, list=20%, signal=35% |
| 424 | TATTATA,MIR-374 |  | 221 | 0.31 | 1.39 | 0.011 | 0.191 | 1.000 | 3436 | tags=34%, list=26%, signal=45% |
| 425 | AGUIRRE\_PANCREAS\_CHR1 |  | 31 | 0.42 | 1.39 | 0.070 | 0.192 | 1.000 | 2566 | tags=48%, list=19%, signal=60% |
| 426 | HPV31\_DN |  | 44 | 0.39 | 1.39 | 0.055 | 0.192 | 1.000 | 3441 | tags=36%, list=26%, signal=49% |
| 427 | V$RORA1\_01 |  | 171 | 0.32 | 1.39 | 0.008 | 0.192 | 1.000 | 4115 | tags=39%, list=31%, signal=55% |
| 428 | CHR9Q22 |  | 60 | 0.37 | 1.39 | 0.059 | 0.195 | 1.000 | 1328 | tags=23%, list=10%, signal=26% |
| 429 | LAL\_KO\_3MO\_UP |  | 42 | 0.40 | 1.39 | 0.076 | 0.195 | 1.000 | 1940 | tags=29%, list=15%, signal=33% |
| 430 | PASSERINI\_SIGNAL |  | 329 | 0.29 | 1.39 | 0.007 | 0.195 | 1.000 | 3135 | tags=32%, list=24%, signal=41% |
| 431 | TNFALPHA\_30MIN\_UP |  | 40 | 0.40 | 1.39 | 0.038 | 0.195 | 1.000 | 1255 | tags=25%, list=9%, signal=28% |
| 432 | BRACX\_UP |  | 19 | 0.48 | 1.39 | 0.083 | 0.195 | 1.000 | 3459 | tags=47%, list=26%, signal=64% |
| 433 | ADDYA\_K562\_HEMIN\_TREATMENT |  | 70 | 0.36 | 1.39 | 0.036 | 0.196 | 1.000 | 3483 | tags=41%, list=26%, signal=56% |
| 434 | TAKEDA\_NUP8\_HOXA9\_16D\_DN |  | 189 | 0.31 | 1.39 | 0.013 | 0.198 | 1.000 | 3344 | tags=35%, list=25%, signal=47% |
| 435 | HSC\_LTHSC\_FETAL |  | 210 | 0.30 | 1.39 | 0.011 | 0.197 | 1.000 | 2441 | tags=28%, list=18%, signal=33% |
| 436 | TGFBETA\_C5\_UP |  | 17 | 0.48 | 1.38 | 0.101 | 0.198 | 1.000 | 2000 | tags=35%, list=15%, signal=41% |
| 437 | V$HMGIY\_Q6 |  | 164 | 0.32 | 1.38 | 0.017 | 0.199 | 1.000 | 3610 | tags=34%, list=27%, signal=45% |
| 438 | V$CEBPB\_02 |  | 188 | 0.31 | 1.38 | 0.018 | 0.199 | 1.000 | 3588 | tags=36%, list=27%, signal=49% |
| 439 | HSC\_LTHSC\_SHARED |  | 210 | 0.30 | 1.38 | 0.008 | 0.201 | 1.000 | 2441 | tags=28%, list=18%, signal=33% |
| 440 | AGED\_MOUSE\_HYPOTH\_UP |  | 43 | 0.39 | 1.38 | 0.060 | 0.201 | 1.000 | 2769 | tags=40%, list=21%, signal=50% |
| 441 | WTGAAAT\_UNKNOWN |  | 414 | 0.28 | 1.38 | 0.005 | 0.201 | 1.000 | 3365 | tags=32%, list=25%, signal=41% |
| 442 | HANSON\_NFKAPPB\_IND |  | 16 | 0.50 | 1.38 | 0.108 | 0.201 | 1.000 | 4944 | tags=56%, list=37%, signal=89% |
| 443 | PASSERINI\_GROWTH |  | 33 | 0.42 | 1.38 | 0.075 | 0.201 | 1.000 | 2548 | tags=33%, list=19%, signal=41% |
| 444 | V$ERR1\_Q2 |  | 186 | 0.31 | 1.38 | 0.014 | 0.203 | 1.000 | 3347 | tags=33%, list=25%, signal=43% |
| 445 | CALRES\_MOUSE\_NEOCORTEX\_UP |  | 57 | 0.37 | 1.38 | 0.066 | 0.203 | 1.000 | 1548 | tags=25%, list=12%, signal=28% |
| 446 | CAGCAGG,MIR-370 |  | 114 | 0.33 | 1.38 | 0.027 | 0.203 | 1.000 | 3564 | tags=33%, list=27%, signal=45% |
| 447 | CHR20P12 |  | 23 | 0.44 | 1.38 | 0.083 | 0.204 | 1.000 | 1846 | tags=35%, list=14%, signal=40% |
| 448 | GOLDRATH\_MEMORY |  | 47 | 0.38 | 1.38 | 0.077 | 0.204 | 1.000 | 2088 | tags=34%, list=16%, signal=40% |
| 449 | RETT\_UP |  | 28 | 0.42 | 1.37 | 0.072 | 0.206 | 1.000 | 2521 | tags=43%, list=19%, signal=53% |
| 450 | NKTPATHWAY |  | 26 | 0.43 | 1.37 | 0.085 | 0.206 | 1.000 | 4104 | tags=50%, list=31%, signal=72% |
| 451 | IL1RPATHWAY |  | 30 | 0.43 | 1.37 | 0.079 | 0.206 | 1.000 | 2217 | tags=33%, list=17%, signal=40% |
| 452 | V$DBP\_Q6 |  | 184 | 0.31 | 1.37 | 0.030 | 0.208 | 1.000 | 3374 | tags=33%, list=25%, signal=43% |
| 453 | TPA\_SENS\_EARLY\_UP |  | 46 | 0.38 | 1.37 | 0.060 | 0.207 | 1.000 | 3265 | tags=48%, list=25%, signal=63% |
| 454 | CHR20P13 |  | 44 | 0.39 | 1.37 | 0.069 | 0.207 | 1.000 | 1735 | tags=25%, list=13%, signal=29% |
| 455 | ST\_TUMOR\_NECROSIS\_FACTOR\_PATHWAY |  | 29 | 0.43 | 1.37 | 0.083 | 0.207 | 1.000 | 3034 | tags=41%, list=23%, signal=53% |
| 456 | ACCATTT,MIR-522 |  | 129 | 0.32 | 1.37 | 0.031 | 0.207 | 1.000 | 4168 | tags=39%, list=31%, signal=56% |
| 457 | ROME\_INSULIN\_2F\_UP |  | 167 | 0.31 | 1.37 | 0.022 | 0.208 | 1.000 | 3471 | tags=38%, list=26%, signal=51% |
| 458 | ALZHEIMERS\_INCIPIENT\_DN |  | 144 | 0.32 | 1.37 | 0.038 | 0.208 | 1.000 | 2856 | tags=32%, list=21%, signal=40% |
| 459 | V$CART1\_01 |  | 145 | 0.32 | 1.37 | 0.015 | 0.207 | 1.000 | 3148 | tags=29%, list=24%, signal=38% |
| 460 | MRNA\_PROCESSING\_REACTOME |  | 102 | 0.33 | 1.37 | 0.052 | 0.207 | 1.000 | 4942 | tags=50%, list=37%, signal=79% |
| 461 | ASTIER\_FN\_DIFF |  | 61 | 0.36 | 1.37 | 0.057 | 0.207 | 1.000 | 2363 | tags=30%, list=18%, signal=36% |
| 462 | ATGCTGC,MIR-103,MIR-107 |  | 163 | 0.31 | 1.37 | 0.017 | 0.210 | 1.000 | 2680 | tags=27%, list=20%, signal=33% |
| 463 | LIZUKA\_G1\_SM\_G2 |  | 25 | 0.43 | 1.37 | 0.079 | 0.209 | 1.000 | 3682 | tags=48%, list=28%, signal=66% |
| 464 | LEE\_MYC\_TGFA\_UP |  | 60 | 0.36 | 1.37 | 0.050 | 0.209 | 1.000 | 1503 | tags=27%, list=11%, signal=30% |
| 465 | GN\_CAMP\_GRANULOSA\_UP |  | 53 | 0.37 | 1.37 | 0.076 | 0.210 | 1.000 | 2727 | tags=36%, list=20%, signal=45% |
| 466 | CHR2P12 |  | 21 | 0.46 | 1.37 | 0.096 | 0.210 | 1.000 | 3910 | tags=57%, list=29%, signal=81% |
| 467 | SANSOM\_APC\_LOSS4\_UP |  | 100 | 0.33 | 1.37 | 0.051 | 0.211 | 1.000 | 3638 | tags=44%, list=27%, signal=60% |
| 468 | TTAYRTAA\_V$E4BP4\_01 |  | 173 | 0.31 | 1.37 | 0.024 | 0.211 | 1.000 | 2450 | tags=24%, list=18%, signal=29% |
| 469 | CHAUHAN\_2ME2 |  | 46 | 0.38 | 1.37 | 0.074 | 0.211 | 1.000 | 2838 | tags=37%, list=21%, signal=47% |
| 470 | CTGRYYYNATT\_UNKNOWN |  | 49 | 0.38 | 1.37 | 0.078 | 0.211 | 1.000 | 2418 | tags=31%, list=18%, signal=37% |
| 471 | ST\_PHOSPHOINOSITIDE\_3\_KINASE\_PATHWAY |  | 33 | 0.41 | 1.37 | 0.095 | 0.211 | 1.000 | 3014 | tags=39%, list=23%, signal=51% |
| 472 | GTGTGAG,MIR-342 |  | 56 | 0.37 | 1.36 | 0.062 | 0.212 | 1.000 | 3936 | tags=46%, list=30%, signal=66% |
| 473 | METASTASIS\_ADENOCARC\_DN |  | 34 | 0.40 | 1.36 | 0.097 | 0.215 | 1.000 | 2842 | tags=35%, list=21%, signal=45% |
| 474 | GAGCCTG,MIR-484 |  | 78 | 0.35 | 1.36 | 0.048 | 0.215 | 1.000 | 4026 | tags=45%, list=30%, signal=64% |
| 475 | PYRUVATE\_METABOLISM |  | 35 | 0.41 | 1.36 | 0.097 | 0.216 | 1.000 | 842 | tags=23%, list=6%, signal=24% |
| 476 | IFNA\_HCMV\_6HRS\_UP |  | 53 | 0.37 | 1.36 | 0.071 | 0.219 | 1.000 | 3118 | tags=36%, list=23%, signal=47% |
| 477 | UEDA\_MOUSE\_LIVER |  | 119 | 0.32 | 1.36 | 0.026 | 0.219 | 1.000 | 1474 | tags=22%, list=11%, signal=24% |
| 478 | ST\_WNT\_CA2\_CYCLIC\_GMP\_PATHWAY |  | 19 | 0.47 | 1.36 | 0.090 | 0.219 | 1.000 | 3852 | tags=53%, list=29%, signal=74% |
| 479 | PARK\_RARALPHA\_UP |  | 37 | 0.40 | 1.36 | 0.090 | 0.220 | 1.000 | 3122 | tags=46%, list=23%, signal=60% |
| 480 | V$AP1\_01 |  | 186 | 0.30 | 1.36 | 0.026 | 0.220 | 1.000 | 3821 | tags=38%, list=29%, signal=52% |
| 481 | IFN\_ALPHA\_UP |  | 40 | 0.39 | 1.36 | 0.081 | 0.220 | 1.000 | 3118 | tags=35%, list=23%, signal=46% |
| 482 | CHR10P12 |  | 18 | 0.48 | 1.36 | 0.105 | 0.220 | 1.000 | 3904 | tags=56%, list=29%, signal=78% |
| 483 | V$TCF4\_Q5 |  | 165 | 0.31 | 1.36 | 0.021 | 0.220 | 1.000 | 2306 | tags=25%, list=17%, signal=30% |
| 484 | TAKEDA\_NUP8\_HOXA9\_3D\_DN |  | 27 | 0.43 | 1.36 | 0.085 | 0.220 | 1.000 | 4931 | tags=56%, list=37%, signal=88% |
| 485 | CACCAGC,MIR-138 |  | 153 | 0.31 | 1.36 | 0.022 | 0.220 | 1.000 | 3571 | tags=37%, list=27%, signal=49% |
| 486 | CHEN\_LUNG\_SURVIVAL |  | 20 | 0.46 | 1.35 | 0.111 | 0.220 | 1.000 | 1262 | tags=25%, list=9%, signal=28% |
| 487 | NAKAJIMA\_MCS\_UP |  | 91 | 0.33 | 1.35 | 0.048 | 0.221 | 1.000 | 3899 | tags=41%, list=29%, signal=57% |
| 488 | IFN\_ALL\_UP |  | 18 | 0.47 | 1.35 | 0.119 | 0.222 | 1.000 | 5152 | tags=61%, list=39%, signal=100% |
| 489 | V$MEF2\_04 |  | 20 | 0.46 | 1.35 | 0.123 | 0.222 | 1.000 | 1820 | tags=30%, list=14%, signal=35% |
| 490 | V$VDR\_Q6 |  | 170 | 0.30 | 1.35 | 0.024 | 0.222 | 1.000 | 3938 | tags=39%, list=30%, signal=54% |
| 491 | V$PAX6\_01 |  | 67 | 0.35 | 1.35 | 0.065 | 0.222 | 1.000 | 4048 | tags=39%, list=30%, signal=55% |
| 492 | LEE\_E2F1\_DN |  | 62 | 0.35 | 1.35 | 0.075 | 0.226 | 1.000 | 4418 | tags=44%, list=33%, signal=65% |
| 493 | TCTAGAG,MIR-517 |  | 37 | 0.40 | 1.35 | 0.074 | 0.225 | 1.000 | 2042 | tags=30%, list=15%, signal=35% |
| 494 | V$MYCMAX\_02 |  | 194 | 0.30 | 1.35 | 0.017 | 0.225 | 1.000 | 3289 | tags=33%, list=25%, signal=43% |
| 495 | CHR18Q12 |  | 35 | 0.39 | 1.35 | 0.098 | 0.230 | 1.000 | 1077 | tags=23%, list=8%, signal=25% |
| 496 | ARFPATHWAY |  | 15 | 0.49 | 1.34 | 0.125 | 0.233 | 1.000 | 3330 | tags=33%, list=25%, signal=44% |
| 497 | IL6\_FIBRO\_UP |  | 47 | 0.37 | 1.34 | 0.090 | 0.235 | 1.000 | 2646 | tags=34%, list=20%, signal=42% |
| 498 | UV-CMV\_UNIQUE\_HCMV\_6HRS\_UP |  | 102 | 0.33 | 1.34 | 0.044 | 0.235 | 1.000 | 3118 | tags=32%, list=23%, signal=42% |
| 499 | GTCAACC,MIR-380-5P |  | 16 | 0.48 | 1.34 | 0.121 | 0.237 | 1.000 | 2201 | tags=44%, list=17%, signal=52% |
| 500 | AGUIRRE\_PANCREAS\_CHR22 |  | 60 | 0.36 | 1.34 | 0.079 | 0.238 | 1.000 | 4314 | tags=47%, list=32%, signal=69% |
| 501 | BRENTANI\_CYTOSKELETON |  | 22 | 0.44 | 1.34 | 0.102 | 0.238 | 1.000 | 2943 | tags=36%, list=22%, signal=47% |
| 502 | UNDERHILL\_PROLIFERATION |  | 16 | 0.49 | 1.34 | 0.128 | 0.238 | 1.000 | 1622 | tags=44%, list=12%, signal=50% |
| 503 | CMV\_HCMV\_TIMECOURSE\_14HRS\_UP |  | 44 | 0.38 | 1.34 | 0.087 | 0.237 | 1.000 | 2019 | tags=27%, list=15%, signal=32% |
| 504 | NO2IL12PATHWAY |  | 15 | 0.49 | 1.34 | 0.126 | 0.238 | 1.000 | 5305 | tags=60%, list=40%, signal=100% |
| 505 | STEMPATHWAY |  | 15 | 0.49 | 1.34 | 0.127 | 0.238 | 1.000 | 5443 | tags=60%, list=41%, signal=101% |
| 506 | GTCAGGA,MIR-378 |  | 38 | 0.38 | 1.34 | 0.100 | 0.239 | 1.000 | 1387 | tags=21%, list=10%, signal=23% |
| 507 | ET743\_HELA\_DN |  | 15 | 0.49 | 1.34 | 0.126 | 0.239 | 1.000 | 733 | tags=33%, list=6%, signal=35% |
| 508 | ASTIER\_BCELL |  | 60 | 0.35 | 1.34 | 0.085 | 0.240 | 1.000 | 2363 | tags=28%, list=18%, signal=34% |
| 509 | CAATGCA,MIR-33 |  | 75 | 0.34 | 1.34 | 0.069 | 0.240 | 1.000 | 2550 | tags=25%, list=19%, signal=31% |
| 510 | TGANTCA\_V$AP1\_C |  | 786 | 0.26 | 1.34 | 0.001 | 0.239 | 1.000 | 3610 | tags=33%, list=27%, signal=42% |
| 511 | TELPATHWAY |  | 15 | 0.49 | 1.34 | 0.125 | 0.239 | 1.000 | 2959 | tags=33%, list=22%, signal=43% |
| 512 | REOVIRUS\_HEK293\_UP |  | 235 | 0.29 | 1.34 | 0.025 | 0.239 | 1.000 | 2365 | tags=24%, list=18%, signal=29% |
| 513 | ST\_GA13\_PATHWAY |  | 35 | 0.39 | 1.34 | 0.103 | 0.241 | 1.000 | 3368 | tags=34%, list=25%, signal=46% |
| 514 | SHEPARD\_NEG\_REG\_OF\_CELL\_PROLIFERATION |  | 105 | 0.32 | 1.33 | 0.052 | 0.241 | 1.000 | 2231 | tags=24%, list=17%, signal=28% |
| 515 | CHRXQ24 |  | 19 | 0.45 | 1.33 | 0.095 | 0.240 | 1.000 | 3994 | tags=58%, list=30%, signal=83% |
| 516 | UVC\_TTD-XPCS\_COMMON\_DN |  | 144 | 0.31 | 1.33 | 0.030 | 0.242 | 1.000 | 2199 | tags=24%, list=17%, signal=29% |
| 517 | LEE\_TCELLS4\_UP |  | 52 | 0.36 | 1.33 | 0.072 | 0.242 | 1.000 | 3212 | tags=40%, list=24%, signal=53% |
| 518 | DNA\_DAMAGE\_SIGNALING |  | 87 | 0.33 | 1.33 | 0.077 | 0.242 | 1.000 | 2105 | tags=25%, list=16%, signal=30% |
| 519 | XU\_ATRA\_PLUSNSC\_UP |  | 15 | 0.49 | 1.33 | 0.132 | 0.244 | 1.000 | 1510 | tags=33%, list=11%, signal=38% |
| 520 | ADIPOGENESIS\_HMSC\_CLASS8\_DN |  | 32 | 0.40 | 1.33 | 0.096 | 0.244 | 1.000 | 1992 | tags=28%, list=15%, signal=33% |
| 521 | V$CEBPA\_01 |  | 166 | 0.30 | 1.33 | 0.033 | 0.246 | 1.000 | 2278 | tags=25%, list=17%, signal=29% |
| 522 | PASSERINI\_PROLIFERATION |  | 63 | 0.35 | 1.33 | 0.069 | 0.246 | 1.000 | 1486 | tags=24%, list=11%, signal=27% |
| 523 | ST\_DICTYOSTELIUM\_DISCOIDEUM\_CAMP\_CHEMOTAXIS\_PATHWAY |  | 30 | 0.40 | 1.33 | 0.094 | 0.246 | 1.000 | 4212 | tags=53%, list=32%, signal=78% |
| 524 | BRENTANI\_DEATH |  | 69 | 0.34 | 1.33 | 0.079 | 0.246 | 1.000 | 2744 | tags=35%, list=21%, signal=44% |
| 525 | V$TST1\_01 |  | 180 | 0.30 | 1.33 | 0.030 | 0.246 | 1.000 | 3146 | tags=31%, list=24%, signal=39% |
| 526 | SHEPARD\_POS\_REG\_OF\_CELL\_PROLIFERATION |  | 88 | 0.33 | 1.33 | 0.070 | 0.247 | 1.000 | 4104 | tags=40%, list=31%, signal=57% |
| 527 | POMEROY\_MD\_TREATMENT\_GOOD\_VS\_POOR\_DN |  | 24 | 0.44 | 1.33 | 0.118 | 0.248 | 1.000 | 3105 | tags=46%, list=23%, signal=60% |
| 528 | V$GATA1\_02 |  | 169 | 0.30 | 1.33 | 0.037 | 0.249 | 1.000 | 2381 | tags=24%, list=18%, signal=28% |
| 529 | SIG\_CHEMOTAXIS |  | 42 | 0.38 | 1.33 | 0.097 | 0.250 | 1.000 | 2329 | tags=29%, list=17%, signal=35% |
| 530 | RAY\_P210\_DIFF |  | 53 | 0.36 | 1.32 | 0.091 | 0.250 | 1.000 | 2873 | tags=34%, list=22%, signal=43% |
| 531 | V$ISRE\_01 |  | 164 | 0.30 | 1.32 | 0.032 | 0.250 | 1.000 | 3423 | tags=33%, list=26%, signal=44% |
| 532 | CHR1P22 |  | 49 | 0.37 | 1.32 | 0.078 | 0.250 | 1.000 | 2041 | tags=27%, list=15%, signal=31% |
| 533 | GTGCCTT,MIR-506 |  | 495 | 0.27 | 1.32 | 0.006 | 0.250 | 1.000 | 3107 | tags=30%, list=23%, signal=38% |
| 534 | NADLER\_OBESITY\_HYPERGLYCEMIA |  | 42 | 0.37 | 1.32 | 0.098 | 0.256 | 1.000 | 2421 | tags=31%, list=18%, signal=38% |
| 535 | ACAWYAAAG\_UNKNOWN |  | 71 | 0.33 | 1.32 | 0.087 | 0.255 | 1.000 | 2234 | tags=25%, list=17%, signal=30% |
| 536 | DAC\_BLADDER\_UP |  | 28 | 0.41 | 1.32 | 0.109 | 0.255 | 1.000 | 2270 | tags=32%, list=17%, signal=39% |
| 537 | V$AP1FJ\_Q2 |  | 201 | 0.30 | 1.32 | 0.019 | 0.256 | 1.000 | 4198 | tags=42%, list=32%, signal=61% |
| 538 | V$PTF1BETA\_Q6 |  | 169 | 0.30 | 1.32 | 0.045 | 0.256 | 1.000 | 2674 | tags=28%, list=20%, signal=34% |
| 539 | AGGCACT,MIR-515-3P |  | 65 | 0.35 | 1.32 | 0.079 | 0.256 | 1.000 | 2156 | tags=29%, list=16%, signal=35% |
| 540 | SIG\_BCR\_SIGNALING\_PATHWAY |  | 44 | 0.36 | 1.32 | 0.100 | 0.257 | 1.000 | 3460 | tags=39%, list=26%, signal=52% |
| 541 | V$MYOGNF1\_01 |  | 32 | 0.40 | 1.32 | 0.109 | 0.257 | 1.000 | 2326 | tags=28%, list=17%, signal=34% |
| 542 | JAIN\_NEMO\_DIFF |  | 74 | 0.34 | 1.32 | 0.066 | 0.258 | 1.000 | 4061 | tags=43%, list=30%, signal=62% |
| 543 | CAMPTOTHECIN\_PROBCELL\_DN |  | 28 | 0.41 | 1.32 | 0.121 | 0.259 | 1.000 | 2920 | tags=50%, list=22%, signal=64% |
| 544 | CHR9P13 |  | 43 | 0.37 | 1.32 | 0.078 | 0.259 | 1.000 | 3413 | tags=40%, list=26%, signal=53% |
| 545 | BRCA1\_OVEREXP\_PROSTATE\_DN |  | 77 | 0.34 | 1.32 | 0.085 | 0.259 | 1.000 | 2986 | tags=36%, list=22%, signal=47% |
| 546 | FERRANDO\_T\_CELL\_DIFFERENTIATION\_PATHWAY |  | 18 | 0.45 | 1.32 | 0.127 | 0.259 | 1.000 | 4272 | tags=61%, list=32%, signal=90% |
| 547 | INTEGRIN\_MEDIATED\_CELL\_ADHESION\_KEGG |  | 84 | 0.33 | 1.31 | 0.070 | 0.261 | 1.000 | 3505 | tags=40%, list=26%, signal=55% |
| 548 | GOLDRATH\_HP |  | 129 | 0.31 | 1.31 | 0.064 | 0.261 | 1.000 | 2614 | tags=30%, list=20%, signal=37% |
| 549 | BCNU\_GLIOMA\_MGMT\_48HRS\_UP |  | 18 | 0.46 | 1.31 | 0.145 | 0.261 | 1.000 | 2247 | tags=33%, list=17%, signal=40% |
| 550 | V$CIZ\_01 |  | 164 | 0.30 | 1.31 | 0.048 | 0.261 | 1.000 | 3796 | tags=40%, list=28%, signal=55% |
| 551 | ATGCAGT,MIR-217 |  | 81 | 0.33 | 1.31 | 0.072 | 0.261 | 1.000 | 4002 | tags=40%, list=30%, signal=56% |
| 552 | CELL\_SURFACE\_RECEPTOR\_LINKED\_SIGNAL\_TRANSDUCTION |  | 128 | 0.30 | 1.31 | 0.049 | 0.261 | 1.000 | 3425 | tags=33%, list=26%, signal=44% |
| 553 | HADDAD\_HSC\_CD7\_UP |  | 64 | 0.34 | 1.31 | 0.084 | 0.262 | 1.000 | 4182 | tags=42%, list=31%, signal=61% |
| 554 | GAGCCAG,MIR-149 |  | 108 | 0.31 | 1.31 | 0.062 | 0.262 | 1.000 | 2201 | tags=26%, list=17%, signal=31% |
| 555 | V$E2F1\_Q3\_01 |  | 174 | 0.30 | 1.31 | 0.039 | 0.262 | 1.000 | 3475 | tags=36%, list=26%, signal=48% |
| 556 | CHRXQ26 |  | 30 | 0.40 | 1.31 | 0.114 | 0.262 | 1.000 | 1997 | tags=30%, list=15%, signal=35% |
| 557 | V$CREL\_01 |  | 186 | 0.29 | 1.31 | 0.029 | 0.262 | 1.000 | 3610 | tags=35%, list=27%, signal=47% |
| 558 | YAGI\_AML\_PROG\_FAB |  | 190 | 0.29 | 1.31 | 0.034 | 0.264 | 1.000 | 4342 | tags=45%, list=33%, signal=65% |
| 559 | CHR13Q32 |  | 15 | 0.47 | 1.31 | 0.151 | 0.264 | 1.000 | 4803 | tags=60%, list=36%, signal=94% |
| 560 | ATGTCAC,MIR-489 |  | 72 | 0.33 | 1.31 | 0.067 | 0.264 | 1.000 | 3119 | tags=35%, list=23%, signal=45% |
| 561 | AAACCAC,MIR-140 |  | 81 | 0.33 | 1.31 | 0.068 | 0.264 | 1.000 | 3561 | tags=38%, list=27%, signal=52% |
| 562 | VEGF\_HUVEC\_2HRS\_UP |  | 30 | 0.41 | 1.31 | 0.122 | 0.264 | 1.000 | 5464 | tags=70%, list=41%, signal=118% |
| 563 | V$HSF\_Q6 |  | 128 | 0.31 | 1.31 | 0.051 | 0.264 | 1.000 | 1321 | tags=18%, list=10%, signal=20% |
| 564 | UVB\_SCC\_DN |  | 104 | 0.32 | 1.31 | 0.062 | 0.264 | 1.000 | 2058 | tags=27%, list=15%, signal=32% |
| 565 | CCTGCTG,MIR-214 |  | 174 | 0.30 | 1.31 | 0.040 | 0.263 | 1.000 | 2961 | tags=32%, list=22%, signal=40% |
| 566 | STEFFEN\_AML\_PML\_PLZF\_TRGT |  | 42 | 0.37 | 1.31 | 0.086 | 0.263 | 1.000 | 3265 | tags=40%, list=25%, signal=53% |
| 567 | BRENTANI\_DNA\_METHYLATION\_AND\_MODIFICATION |  | 23 | 0.43 | 1.31 | 0.128 | 0.263 | 1.000 | 370 | tags=17%, list=3%, signal=18% |
| 568 | CCR3PATHWAY |  | 22 | 0.43 | 1.31 | 0.142 | 0.264 | 1.000 | 3899 | tags=50%, list=29%, signal=71% |
| 569 | CHR16Q24 |  | 58 | 0.35 | 1.31 | 0.095 | 0.263 | 1.000 | 3650 | tags=34%, list=27%, signal=47% |
| 570 | CELL\_ADHESION\_MOLECULE\_ACTIVITY |  | 99 | 0.32 | 1.31 | 0.071 | 0.263 | 1.000 | 1586 | tags=17%, list=12%, signal=19% |
| 571 | HUMAN\_TISSUE\_THYMUS |  | 16 | 0.46 | 1.31 | 0.146 | 0.263 | 1.000 | 2359 | tags=38%, list=18%, signal=46% |
| 572 | FERRANDO\_MLL\_T\_ALL\_UP |  | 85 | 0.33 | 1.31 | 0.077 | 0.265 | 1.000 | 2762 | tags=33%, list=21%, signal=41% |
| 573 | CALRES\_MOUSE\_NEOCORTEX\_DN |  | 63 | 0.34 | 1.31 | 0.099 | 0.265 | 1.000 | 3099 | tags=35%, list=23%, signal=45% |
| 574 | GAMMA\_ESR\_WS\_UNREG |  | 26 | 0.41 | 1.30 | 0.150 | 0.266 | 1.000 | 4210 | tags=50%, list=32%, signal=73% |
| 575 | MYOD\_NIH3T3\_UP |  | 75 | 0.33 | 1.30 | 0.098 | 0.266 | 1.000 | 2958 | tags=33%, list=22%, signal=43% |
| 576 | V$HFH4\_01 |  | 149 | 0.30 | 1.30 | 0.063 | 0.266 | 1.000 | 3337 | tags=34%, list=25%, signal=44% |
| 577 | V$MEIS1BHOXA9\_01 |  | 97 | 0.32 | 1.30 | 0.070 | 0.266 | 1.000 | 2356 | tags=24%, list=18%, signal=29% |
| 578 | V$E2F\_Q2 |  | 118 | 0.31 | 1.30 | 0.078 | 0.267 | 1.000 | 3347 | tags=31%, list=25%, signal=42% |
| 579 | CHR4Q21 |  | 62 | 0.34 | 1.30 | 0.097 | 0.269 | 1.000 | 3558 | tags=31%, list=27%, signal=42% |
| 580 | UVC\_HIGH\_D4\_DN |  | 46 | 0.36 | 1.30 | 0.120 | 0.270 | 1.000 | 4453 | tags=52%, list=33%, signal=78% |
| 581 | HOFMANN\_MDS\_CD34\_HIGH\_RISK |  | 28 | 0.41 | 1.30 | 0.133 | 0.269 | 1.000 | 2811 | tags=32%, list=21%, signal=41% |
| 582 | HOGERKORP\_CD44\_UP |  | 21 | 0.43 | 1.30 | 0.132 | 0.269 | 1.000 | 1988 | tags=29%, list=15%, signal=34% |
| 583 | V$BACH2\_01 |  | 192 | 0.29 | 1.30 | 0.043 | 0.269 | 1.000 | 2834 | tags=26%, list=21%, signal=32% |
| 584 | V$FOXD3\_01 |  | 143 | 0.30 | 1.30 | 0.041 | 0.269 | 1.000 | 3444 | tags=37%, list=26%, signal=49% |
| 585 | V$LEF1\_Q2 |  | 139 | 0.30 | 1.30 | 0.058 | 0.269 | 1.000 | 3842 | tags=37%, list=29%, signal=52% |
| 586 | BRCA2\_BRCA1\_UP |  | 47 | 0.36 | 1.30 | 0.098 | 0.271 | 1.000 | 2279 | tags=30%, list=17%, signal=36% |
| 587 | V$CEBPB\_01 |  | 191 | 0.29 | 1.30 | 0.047 | 0.273 | 1.000 | 3651 | tags=37%, list=27%, signal=50% |
| 588 | TGACAGNY\_V$MEIS1\_01 |  | 562 | 0.26 | 1.30 | 0.012 | 0.273 | 1.000 | 3937 | tags=36%, list=30%, signal=49% |
| 589 | GCAAGGA,MIR-502 |  | 51 | 0.35 | 1.30 | 0.104 | 0.275 | 1.000 | 2467 | tags=31%, list=19%, signal=38% |
| 590 | CMV\_24HRS\_UP |  | 69 | 0.34 | 1.30 | 0.090 | 0.275 | 1.000 | 3145 | tags=30%, list=24%, signal=40% |
| 591 | GALACTOSE\_METABOLISM |  | 23 | 0.42 | 1.30 | 0.129 | 0.275 | 1.000 | 3083 | tags=48%, list=23%, signal=62% |
| 592 | RCC\_NL\_UP |  | 443 | 0.27 | 1.30 | 0.016 | 0.275 | 1.000 | 3357 | tags=31%, list=25%, signal=41% |
| 593 | IL6PATHWAY |  | 21 | 0.44 | 1.29 | 0.126 | 0.276 | 1.000 | 2391 | tags=43%, list=18%, signal=52% |
| 594 | GH\_GHRHR\_KO\_24HRS\_UP |  | 115 | 0.31 | 1.29 | 0.085 | 0.276 | 1.000 | 2182 | tags=23%, list=16%, signal=28% |
| 595 | DER\_IFNA\_UP |  | 65 | 0.34 | 1.29 | 0.097 | 0.278 | 1.000 | 3139 | tags=34%, list=24%, signal=44% |
| 596 | HADDAD\_CD45CD7\_PLUS\_VS\_MINUS\_UP |  | 64 | 0.34 | 1.29 | 0.097 | 0.281 | 1.000 | 4182 | tags=42%, list=31%, signal=61% |
| 597 | CTTTGA\_V$LEF1\_Q2 |  | 793 | 0.26 | 1.29 | 0.003 | 0.282 | 1.000 | 3782 | tags=33%, list=28%, signal=43% |
| 598 | PASSERINI\_INFLAMMATION |  | 24 | 0.42 | 1.29 | 0.138 | 0.283 | 1.000 | 3328 | tags=46%, list=25%, signal=61% |
| 599 | OXSTRESS\_RPE\_H2O2HNE\_DN |  | 31 | 0.40 | 1.29 | 0.119 | 0.283 | 1.000 | 2530 | tags=35%, list=19%, signal=44% |
| 600 | GTACAGG,MIR-486 |  | 49 | 0.35 | 1.29 | 0.094 | 0.282 | 1.000 | 3647 | tags=37%, list=27%, signal=50% |
| 601 | CHR13Q14 |  | 59 | 0.35 | 1.29 | 0.109 | 0.284 | 1.000 | 3273 | tags=34%, list=25%, signal=45% |
| 602 | MARCINIAK\_CHOP\_DIFF |  | 19 | 0.44 | 1.29 | 0.145 | 0.288 | 1.000 | 1714 | tags=26%, list=13%, signal=30% |
| 603 | AAGCCAT,MIR-135A,MIR-135B |  | 249 | 0.28 | 1.29 | 0.046 | 0.289 | 1.000 | 3817 | tags=36%, list=29%, signal=49% |
| 604 | V$ELF1\_Q6 |  | 168 | 0.29 | 1.28 | 0.054 | 0.291 | 1.000 | 4043 | tags=39%, list=30%, signal=55% |
| 605 | CTTTGT\_V$LEF1\_Q2 |  | 1325 | 0.25 | 1.28 | 0.002 | 0.290 | 1.000 | 3436 | tags=30%, list=26%, signal=37% |
| 606 | TAVOR\_CEBP\_UP |  | 49 | 0.36 | 1.28 | 0.133 | 0.290 | 1.000 | 3903 | tags=53%, list=29%, signal=75% |
| 607 | V$CEBP\_Q2\_01 |  | 197 | 0.28 | 1.28 | 0.055 | 0.290 | 1.000 | 3183 | tags=29%, list=24%, signal=38% |
| 608 | SMITH\_HCV\_INDUCED\_HCC\_UP |  | 33 | 0.39 | 1.28 | 0.134 | 0.290 | 1.000 | 1827 | tags=30%, list=14%, signal=35% |
| 609 | VEGF\_MMMEC\_ALL\_UP |  | 91 | 0.31 | 1.28 | 0.091 | 0.291 | 1.000 | 2270 | tags=30%, list=17%, signal=36% |
| 610 | AATGTGA,MIR-23A,MIR-23B |  | 329 | 0.27 | 1.28 | 0.025 | 0.291 | 1.000 | 2449 | tags=24%, list=18%, signal=28% |
| 611 | V$NFY\_01 |  | 178 | 0.29 | 1.28 | 0.044 | 0.293 | 1.000 | 2888 | tags=29%, list=22%, signal=37% |
| 612 | CHR2P22 |  | 24 | 0.41 | 1.28 | 0.150 | 0.293 | 1.000 | 4027 | tags=54%, list=30%, signal=77% |
| 613 | HINATA\_NFKB\_DN |  | 20 | 0.44 | 1.28 | 0.155 | 0.294 | 1.000 | 2199 | tags=45%, list=17%, signal=54% |
| 614 | V$TCF11MAFG\_01 |  | 144 | 0.29 | 1.28 | 0.072 | 0.293 | 1.000 | 4048 | tags=40%, list=30%, signal=57% |
| 615 | AGGAGTG,MIR-483 |  | 53 | 0.35 | 1.28 | 0.118 | 0.293 | 1.000 | 3125 | tags=34%, list=23%, signal=44% |
| 616 | PGC |  | 343 | 0.27 | 1.28 | 0.036 | 0.294 | 1.000 | 3489 | tags=33%, list=26%, signal=43% |
| 617 | CACGTG\_V$MYC\_Q2 |  | 715 | 0.26 | 1.28 | 0.005 | 0.294 | 1.000 | 3059 | tags=28%, list=23%, signal=35% |
| 618 | AAANWWTGC\_UNKNOWN |  | 142 | 0.30 | 1.28 | 0.074 | 0.294 | 1.000 | 3937 | tags=40%, list=30%, signal=56% |
| 619 | LYSINE\_DEGRADATION |  | 28 | 0.39 | 1.28 | 0.136 | 0.294 | 1.000 | 2904 | tags=32%, list=22%, signal=41% |
| 620 | V$NFKB\_Q6 |  | 186 | 0.28 | 1.28 | 0.049 | 0.294 | 1.000 | 3797 | tags=37%, list=29%, signal=50% |
| 621 | ATACTGT,MIR-144 |  | 161 | 0.29 | 1.28 | 0.049 | 0.295 | 1.000 | 3872 | tags=37%, list=29%, signal=51% |
| 622 | TGCTGCT,MIR-15A,MIR-16,MIR-15B,MIR-195,MIR-424,MIR-497 |  | 459 | 0.26 | 1.28 | 0.016 | 0.295 | 1.000 | 2766 | tags=25%, list=21%, signal=30% |
| 623 | HOFFMANN\_BIVSBII\_BI |  | 88 | 0.31 | 1.28 | 0.087 | 0.296 | 1.000 | 3943 | tags=40%, list=30%, signal=56% |
| 624 | WNT\_TARGETS |  | 25 | 0.41 | 1.28 | 0.155 | 0.296 | 1.000 | 1060 | tags=24%, list=8%, signal=26% |
| 625 | V$CEBP\_Q2 |  | 162 | 0.29 | 1.28 | 0.057 | 0.297 | 1.000 | 3608 | tags=34%, list=27%, signal=46% |
| 626 | LEE\_DENA\_UP |  | 59 | 0.34 | 1.28 | 0.117 | 0.297 | 1.000 | 2861 | tags=41%, list=21%, signal=52% |
| 627 | AACYNNNNTTCCS\_UNKNOWN |  | 62 | 0.34 | 1.27 | 0.122 | 0.298 | 1.000 | 3293 | tags=34%, list=25%, signal=45% |
| 628 | V$GR\_Q6 |  | 182 | 0.28 | 1.27 | 0.069 | 0.299 | 1.000 | 2234 | tags=23%, list=17%, signal=27% |
| 629 | ST\_ADRENERGIC |  | 33 | 0.38 | 1.27 | 0.152 | 0.300 | 1.000 | 1229 | tags=21%, list=9%, signal=23% |
| 630 | HDACI\_COLON\_SUL24HRS\_UP |  | 55 | 0.34 | 1.27 | 0.111 | 0.299 | 1.000 | 2391 | tags=31%, list=18%, signal=38% |
| 631 | HSC\_STHSC\_SHARED |  | 26 | 0.40 | 1.27 | 0.156 | 0.300 | 1.000 | 1443 | tags=31%, list=11%, signal=34% |
| 632 | 5FU\_RESIST\_GASTRIC\_UP |  | 21 | 0.42 | 1.27 | 0.167 | 0.300 | 1.000 | 829 | tags=24%, list=6%, signal=25% |
| 633 | GLYCOLYSIS |  | 49 | 0.35 | 1.27 | 0.134 | 0.302 | 1.000 | 1890 | tags=27%, list=14%, signal=31% |
| 634 | CHR4Q31 |  | 41 | 0.36 | 1.27 | 0.131 | 0.304 | 1.000 | 2377 | tags=37%, list=18%, signal=44% |
| 635 | GGCACTT,MIR-519E |  | 100 | 0.31 | 1.27 | 0.089 | 0.304 | 1.000 | 3172 | tags=35%, list=24%, signal=46% |
| 636 | V$NFAT\_Q4\_01 |  | 184 | 0.28 | 1.27 | 0.064 | 0.307 | 1.000 | 3371 | tags=34%, list=25%, signal=44% |
| 637 | ASTON\_DEPRESSION\_DN |  | 149 | 0.29 | 1.27 | 0.072 | 0.306 | 1.000 | 3896 | tags=42%, list=29%, signal=58% |
| 638 | V$OCT1\_05 |  | 182 | 0.28 | 1.27 | 0.072 | 0.307 | 1.000 | 4305 | tags=39%, list=32%, signal=57% |
| 639 | SA\_CASPASE\_CASCADE |  | 16 | 0.45 | 1.27 | 0.178 | 0.308 | 1.000 | 2471 | tags=44%, list=19%, signal=54% |
| 640 | OLDONLY\_FIBRO\_UP |  | 34 | 0.38 | 1.27 | 0.138 | 0.308 | 1.000 | 2226 | tags=29%, list=17%, signal=35% |
| 641 | ABBUD\_LIF\_DN |  | 22 | 0.42 | 1.27 | 0.166 | 0.308 | 1.000 | 1448 | tags=32%, list=11%, signal=36% |
| 642 | CHR4P15 |  | 27 | 0.40 | 1.27 | 0.162 | 0.308 | 1.000 | 1132 | tags=26%, list=8%, signal=28% |
| 643 | GLUCONEOGENESIS |  | 49 | 0.35 | 1.27 | 0.125 | 0.307 | 1.000 | 1890 | tags=27%, list=14%, signal=31% |
| 644 | TRNA\_SYNTHETASES |  | 18 | 0.45 | 1.27 | 0.163 | 0.308 | 1.000 | 2066 | tags=44%, list=16%, signal=53% |
| 645 | HDACI\_COLON\_CLUSTER6 |  | 33 | 0.38 | 1.27 | 0.152 | 0.308 | 1.000 | 3883 | tags=48%, list=29%, signal=68% |
| 646 | GGGNNTTTCC\_V$NFKB\_Q6\_01 |  | 96 | 0.31 | 1.27 | 0.080 | 0.308 | 1.000 | 3484 | tags=35%, list=26%, signal=48% |
| 647 | STEMCELL\_COMMON\_UP |  | 153 | 0.29 | 1.26 | 0.072 | 0.308 | 1.000 | 2726 | tags=31%, list=20%, signal=39% |
| 648 | GH\_EXOGENOUS\_ANY\_DN |  | 83 | 0.32 | 1.26 | 0.101 | 0.309 | 1.000 | 2501 | tags=25%, list=19%, signal=31% |
| 649 | ALZHEIMERS\_DISEASE\_UP |  | 1450 | 0.25 | 1.26 | 0.001 | 0.309 | 1.000 | 3148 | tags=28%, list=24%, signal=32% |
| 650 | SCHURINGA\_STAT5A\_DN |  | 17 | 0.44 | 1.26 | 0.185 | 0.308 | 1.000 | 5145 | tags=71%, list=39%, signal=115% |
| 651 | UVC\_TTD\_8HR\_DN |  | 165 | 0.28 | 1.26 | 0.070 | 0.309 | 1.000 | 1352 | tags=19%, list=10%, signal=21% |
| 652 | MRNA\_PROCESSING |  | 41 | 0.36 | 1.26 | 0.141 | 0.311 | 1.000 | 2921 | tags=32%, list=22%, signal=40% |
| 653 | AGED\_MOUSE\_NEOCORTEX\_UP |  | 61 | 0.34 | 1.26 | 0.123 | 0.312 | 1.000 | 2624 | tags=30%, list=20%, signal=37% |
| 654 | V$IRF1\_01 |  | 177 | 0.28 | 1.26 | 0.071 | 0.314 | 1.000 | 3605 | tags=38%, list=27%, signal=51% |
| 655 | V$AP1\_Q2 |  | 193 | 0.28 | 1.26 | 0.062 | 0.314 | 1.000 | 4198 | tags=40%, list=32%, signal=57% |
| 656 | IFN\_BETA\_GLIOMA\_DN |  | 44 | 0.36 | 1.26 | 0.138 | 0.314 | 1.000 | 3742 | tags=45%, list=28%, signal=63% |
| 657 | V$HOX13\_01 |  | 27 | 0.39 | 1.26 | 0.163 | 0.315 | 1.000 | 1541 | tags=30%, list=12%, signal=33% |
| 658 | WANG\_MLL\_CBP\_VS\_GMP\_DN |  | 38 | 0.36 | 1.26 | 0.142 | 0.315 | 1.000 | 3251 | tags=45%, list=24%, signal=59% |
| 659 | NFKBPATHWAY |  | 23 | 0.41 | 1.26 | 0.157 | 0.316 | 1.000 | 2143 | tags=30%, list=16%, signal=36% |
| 660 | DIAB\_NEPH\_DN |  | 373 | 0.26 | 1.26 | 0.033 | 0.316 | 1.000 | 2087 | tags=23%, list=16%, signal=26% |
| 661 | HBX\_NL\_UP |  | 22 | 0.42 | 1.26 | 0.181 | 0.317 | 1.000 | 4330 | tags=59%, list=33%, signal=87% |
| 662 | AGEING\_BRAIN\_DN |  | 119 | 0.30 | 1.26 | 0.098 | 0.318 | 1.000 | 3621 | tags=34%, list=27%, signal=47% |
| 663 | HSC\_MATURE\_FETAL |  | 251 | 0.27 | 1.26 | 0.057 | 0.317 | 1.000 | 3721 | tags=34%, list=28%, signal=47% |
| 664 | BRCA1\_OVEREXP\_PROSTATE\_UP |  | 154 | 0.28 | 1.26 | 0.074 | 0.319 | 1.000 | 1389 | tags=20%, list=10%, signal=22% |
| 665 | GGCAGAC,MIR-346 |  | 27 | 0.40 | 1.26 | 0.152 | 0.319 | 1.000 | 3369 | tags=41%, list=25%, signal=54% |
| 666 | ADIPOGENESIS\_HMSC\_CLASS1\_UP |  | 18 | 0.44 | 1.26 | 0.176 | 0.319 | 1.000 | 3092 | tags=56%, list=23%, signal=72% |
| 667 | IDX\_TSA\_DN\_CLUSTER1 |  | 41 | 0.35 | 1.25 | 0.152 | 0.319 | 1.000 | 3638 | tags=41%, list=27%, signal=57% |
| 668 | V$AP2GAMMA\_01 |  | 186 | 0.28 | 1.25 | 0.063 | 0.319 | 1.000 | 2947 | tags=26%, list=22%, signal=33% |
| 669 | MYOD\_NIH3T3\_DN |  | 45 | 0.35 | 1.25 | 0.151 | 0.319 | 1.000 | 2154 | tags=27%, list=16%, signal=32% |
| 670 | HSC\_STHSC\_FETAL |  | 26 | 0.40 | 1.25 | 0.168 | 0.319 | 1.000 | 1443 | tags=31%, list=11%, signal=34% |
| 671 | CANCERDRUGS\_PROBCELL\_UP |  | 15 | 0.46 | 1.25 | 0.194 | 0.319 | 1.000 | 2492 | tags=40%, list=19%, signal=49% |
| 672 | AGED\_MOUSE\_CORTEX\_UP |  | 31 | 0.38 | 1.25 | 0.148 | 0.319 | 1.000 | 2118 | tags=39%, list=16%, signal=46% |
| 673 | V$FREAC2\_01 |  | 175 | 0.28 | 1.25 | 0.066 | 0.319 | 1.000 | 3937 | tags=39%, list=30%, signal=54% |
| 674 | WANG\_MLL\_CBP\_VS\_GMP\_UP |  | 32 | 0.38 | 1.25 | 0.159 | 0.319 | 1.000 | 4944 | tags=59%, list=37%, signal=94% |
| 675 | V$AREB6\_04 |  | 178 | 0.28 | 1.25 | 0.073 | 0.319 | 1.000 | 3148 | tags=30%, list=24%, signal=38% |
| 676 | CHR1P36 |  | 230 | 0.27 | 1.25 | 0.061 | 0.319 | 1.000 | 3515 | tags=30%, list=26%, signal=40% |
| 677 | AACTGAC,MIR-223 |  | 77 | 0.32 | 1.25 | 0.125 | 0.318 | 1.000 | 3081 | tags=34%, list=23%, signal=44% |
| 678 | GPCRDB\_CLASS\_B\_SECRETIN\_LIKE |  | 23 | 0.41 | 1.25 | 0.170 | 0.319 | 1.000 | 3459 | tags=39%, list=26%, signal=53% |
| 679 | AGED\_RHESUS\_UP |  | 193 | 0.28 | 1.25 | 0.064 | 0.319 | 1.000 | 4027 | tags=38%, list=30%, signal=54% |
| 680 | MYAATNNNNNNNGGC\_UNKNOWN |  | 92 | 0.30 | 1.25 | 0.102 | 0.320 | 1.000 | 2156 | tags=23%, list=16%, signal=27% |
| 681 | GPCRS\_CLASS\_B\_SECRETIN\_LIKE |  | 23 | 0.41 | 1.25 | 0.171 | 0.322 | 1.000 | 3459 | tags=39%, list=26%, signal=53% |
| 682 | CDMACPATHWAY |  | 16 | 0.44 | 1.25 | 0.195 | 0.323 | 1.000 | 3608 | tags=50%, list=27%, signal=68% |
| 683 | FLECHNER\_KIDNEY\_TRANSPLANT\_WELL\_PBL\_UP |  | 152 | 0.29 | 1.25 | 0.088 | 0.323 | 1.000 | 2266 | tags=26%, list=17%, signal=31% |
| 684 | GGGCATT,MIR-365 |  | 81 | 0.31 | 1.25 | 0.107 | 0.323 | 1.000 | 3775 | tags=36%, list=28%, signal=50% |
| 685 | CORDERO\_KRAS\_KD\_VS\_CONTROL\_UP |  | 74 | 0.32 | 1.25 | 0.113 | 0.323 | 1.000 | 2440 | tags=32%, list=18%, signal=39% |
| 686 | TCCAGAG,MIR-518C |  | 109 | 0.30 | 1.25 | 0.108 | 0.326 | 1.000 | 3489 | tags=34%, list=26%, signal=46% |
| 687 | CMV\_HCMV\_TIMECOURSE\_20HRS\_UP |  | 84 | 0.31 | 1.25 | 0.112 | 0.325 | 1.000 | 2528 | tags=27%, list=19%, signal=34% |
| 688 | V$HNF3\_Q6 |  | 137 | 0.29 | 1.25 | 0.085 | 0.325 | 1.000 | 3444 | tags=33%, list=26%, signal=44% |
| 689 | AAAGGAT,MIR-501 |  | 104 | 0.30 | 1.25 | 0.105 | 0.327 | 1.000 | 2042 | tags=25%, list=15%, signal=29% |
| 690 | V$NCX\_01 |  | 122 | 0.29 | 1.25 | 0.104 | 0.327 | 1.000 | 2426 | tags=26%, list=18%, signal=32% |
| 691 | XU\_CBP\_DN |  | 34 | 0.37 | 1.25 | 0.173 | 0.327 | 1.000 | 1390 | tags=21%, list=10%, signal=23% |
| 692 | BECKER\_TAMOXIFEN\_RESISTANT\_DN |  | 51 | 0.34 | 1.24 | 0.157 | 0.328 | 1.000 | 789 | tags=20%, list=6%, signal=21% |
| 693 | ACATTCC,MIR-1,MIR-206 |  | 231 | 0.27 | 1.24 | 0.069 | 0.330 | 1.000 | 2948 | tags=29%, list=22%, signal=36% |
| 694 | HBX\_HEP\_UP |  | 18 | 0.44 | 1.24 | 0.183 | 0.330 | 1.000 | 2546 | tags=50%, list=19%, signal=62% |
| 695 | TTTTGAG,MIR-373 |  | 178 | 0.28 | 1.24 | 0.079 | 0.332 | 1.000 | 3433 | tags=31%, list=26%, signal=42% |
| 696 | IDX\_TSA\_DN\_CLUSTER4 |  | 24 | 0.41 | 1.24 | 0.164 | 0.333 | 1.000 | 2748 | tags=42%, list=21%, signal=52% |
| 697 | V$NRF2\_Q4 |  | 181 | 0.28 | 1.24 | 0.085 | 0.333 | 1.000 | 2813 | tags=27%, list=21%, signal=34% |
| 698 | ATATGCA,MIR-448 |  | 157 | 0.28 | 1.24 | 0.097 | 0.333 | 1.000 | 3375 | tags=33%, list=25%, signal=44% |
| 699 | GALE\_FLT3ANDAPL\_UP |  | 59 | 0.33 | 1.24 | 0.147 | 0.332 | 1.000 | 3125 | tags=32%, list=23%, signal=42% |
| 700 | DEATHPATHWAY |  | 32 | 0.37 | 1.24 | 0.176 | 0.332 | 1.000 | 2652 | tags=34%, list=20%, signal=43% |
| 701 | TGCACTT,MIR-519C,MIR-519B,MIR-519A |  | 330 | 0.26 | 1.24 | 0.057 | 0.332 | 1.000 | 2704 | tags=26%, list=20%, signal=32% |
| 702 | V$AP1\_C |  | 202 | 0.28 | 1.24 | 0.075 | 0.333 | 1.000 | 4128 | tags=41%, list=31%, signal=58% |
| 703 | TGANNYRGCA\_V$TCF11MAFG\_01 |  | 215 | 0.27 | 1.24 | 0.068 | 0.333 | 1.000 | 3136 | tags=27%, list=24%, signal=34% |
| 704 | V$E2A\_Q2 |  | 166 | 0.28 | 1.24 | 0.078 | 0.334 | 1.000 | 3670 | tags=35%, list=28%, signal=48% |
| 705 | CMV-UV\_HCMV\_6HRS\_UP |  | 120 | 0.30 | 1.24 | 0.109 | 0.335 | 1.000 | 1340 | tags=18%, list=10%, signal=20% |
| 706 | GTAGGCA,MIR-189 |  | 20 | 0.42 | 1.24 | 0.197 | 0.336 | 1.000 | 4068 | tags=45%, list=31%, signal=65% |
| 707 | ASTON\_DEPRESSION\_UP |  | 45 | 0.35 | 1.24 | 0.170 | 0.335 | 1.000 | 2247 | tags=31%, list=17%, signal=37% |
| 708 | LIN\_WNT\_UP |  | 53 | 0.33 | 1.24 | 0.143 | 0.337 | 1.000 | 2943 | tags=32%, list=22%, signal=41% |
| 709 | ZHAN\_MM\_CD138\_MF\_VS\_REST |  | 42 | 0.35 | 1.24 | 0.182 | 0.336 | 1.000 | 2680 | tags=31%, list=20%, signal=39% |
| 710 | AMINOACYL\_TRNA\_BIOSYNTHESIS |  | 21 | 0.42 | 1.24 | 0.188 | 0.337 | 1.000 | 2066 | tags=38%, list=16%, signal=45% |
| 711 | APOPTOSIS\_GENMAPP |  | 43 | 0.35 | 1.24 | 0.171 | 0.339 | 1.000 | 1726 | tags=23%, list=13%, signal=27% |
| 712 | LIZUKA\_L0\_GR\_L1 |  | 15 | 0.45 | 1.23 | 0.227 | 0.339 | 1.000 | 2150 | tags=40%, list=16%, signal=48% |
| 713 | ET743\_RESIST\_DN |  | 40 | 0.35 | 1.23 | 0.170 | 0.341 | 1.000 | 1108 | tags=23%, list=8%, signal=24% |
| 714 | KRETZSCHMAR\_IL6\_DIFF |  | 144 | 0.28 | 1.23 | 0.096 | 0.340 | 1.000 | 2732 | tags=31%, list=21%, signal=38% |
| 715 | V$CP2\_01 |  | 179 | 0.28 | 1.23 | 0.082 | 0.340 | 1.000 | 3701 | tags=37%, list=28%, signal=51% |
| 716 | TGGAAA\_V$NFAT\_Q4\_01 |  | 1292 | 0.24 | 1.23 | 0.005 | 0.341 | 1.000 | 3504 | tags=30%, list=26%, signal=37% |
| 717 | BOQUEST\_CD31PLUS\_VS\_CD31MINUS\_UP |  | 660 | 0.25 | 1.23 | 0.021 | 0.342 | 1.000 | 2848 | tags=27%, list=21%, signal=33% |
| 718 | HSC\_MATURE\_ADULT |  | 270 | 0.26 | 1.23 | 0.061 | 0.343 | 1.000 | 3616 | tags=33%, list=27%, signal=45% |
| 719 | V$TEF1\_Q6 |  | 155 | 0.28 | 1.23 | 0.089 | 0.342 | 1.000 | 3307 | tags=33%, list=25%, signal=43% |
| 720 | V$AR\_Q6 |  | 164 | 0.28 | 1.23 | 0.086 | 0.343 | 1.000 | 3138 | tags=30%, list=24%, signal=39% |
| 721 | GH\_EXOGENOUS\_MIDDLE\_UP |  | 105 | 0.30 | 1.23 | 0.114 | 0.343 | 1.000 | 4604 | tags=41%, list=35%, signal=62% |
| 722 | NI2\_MOUSE\_UP |  | 37 | 0.36 | 1.23 | 0.165 | 0.343 | 1.000 | 1900 | tags=30%, list=14%, signal=35% |
| 723 | AACTGGA,MIR-145 |  | 172 | 0.28 | 1.23 | 0.091 | 0.343 | 1.000 | 2950 | tags=30%, list=22%, signal=38% |
| 724 | HSC\_MATURE\_SHARED |  | 202 | 0.27 | 1.23 | 0.075 | 0.343 | 1.000 | 3662 | tags=34%, list=27%, signal=46% |
| 725 | CHEN\_HOXA5\_TARGETS\_DN |  | 47 | 0.34 | 1.23 | 0.166 | 0.344 | 1.000 | 2381 | tags=28%, list=18%, signal=34% |
| 726 | V$ALPHACP1\_01 |  | 173 | 0.28 | 1.23 | 0.108 | 0.344 | 1.000 | 2790 | tags=29%, list=21%, signal=36% |
| 727 | UVB\_NHEK4\_6HRS\_DN |  | 19 | 0.43 | 1.23 | 0.208 | 0.344 | 1.000 | 2356 | tags=32%, list=18%, signal=38% |
| 728 | ACACTCC,MIR-122A |  | 57 | 0.33 | 1.23 | 0.140 | 0.344 | 1.000 | 2650 | tags=32%, list=20%, signal=39% |
| 729 | UVC\_HIGH\_D1\_DN |  | 15 | 0.45 | 1.23 | 0.195 | 0.344 | 1.000 | 3475 | tags=53%, list=26%, signal=72% |
| 730 | REFRACTORY\_GASTRIC\_UP |  | 87 | 0.30 | 1.23 | 0.134 | 0.345 | 1.000 | 4406 | tags=41%, list=33%, signal=61% |
| 731 | V$AP1\_Q4 |  | 199 | 0.27 | 1.23 | 0.088 | 0.345 | 1.000 | 3163 | tags=32%, list=24%, signal=41% |
| 732 | V$MAX\_01 |  | 191 | 0.27 | 1.23 | 0.101 | 0.347 | 1.000 | 1829 | tags=21%, list=14%, signal=24% |
| 733 | PENG\_GLUCOSE\_UP |  | 33 | 0.37 | 1.23 | 0.170 | 0.348 | 1.000 | 846 | tags=24%, list=6%, signal=26% |
| 734 | BROCKE\_IL6 |  | 144 | 0.28 | 1.23 | 0.119 | 0.348 | 1.000 | 2732 | tags=31%, list=21%, signal=38% |
| 735 | VHL\_RCC\_UP |  | 96 | 0.30 | 1.23 | 0.121 | 0.348 | 1.000 | 3287 | tags=35%, list=25%, signal=47% |
| 736 | V$GATA3\_01 |  | 174 | 0.27 | 1.23 | 0.082 | 0.348 | 1.000 | 2182 | tags=21%, list=16%, signal=24% |
| 737 | AGTTCTC,MIR-146A,MIR-146B |  | 43 | 0.35 | 1.23 | 0.171 | 0.348 | 1.000 | 1751 | tags=23%, list=13%, signal=27% |
| 738 | LEE\_ACOX1\_UP |  | 64 | 0.32 | 1.22 | 0.153 | 0.350 | 1.000 | 2993 | tags=36%, list=22%, signal=46% |
| 739 | CHR2Q32 |  | 28 | 0.38 | 1.22 | 0.191 | 0.350 | 1.000 | 2620 | tags=25%, list=20%, signal=31% |
| 740 | V$GATA6\_01 |  | 184 | 0.27 | 1.22 | 0.096 | 0.350 | 1.000 | 2965 | tags=28%, list=22%, signal=36% |
| 741 | TGAATGT,MIR-181A,MIR-181B,MIR-181C,MIR-181D |  | 368 | 0.25 | 1.22 | 0.067 | 0.350 | 1.000 | 3147 | tags=30%, list=24%, signal=38% |
| 742 | V$ETF\_Q6 |  | 77 | 0.31 | 1.22 | 0.133 | 0.350 | 1.000 | 3283 | tags=31%, list=25%, signal=41% |
| 743 | V$SOX5\_01 |  | 182 | 0.28 | 1.22 | 0.100 | 0.353 | 1.000 | 2202 | tags=23%, list=17%, signal=27% |
| 744 | UVB\_NHEK3\_C8 |  | 65 | 0.32 | 1.22 | 0.157 | 0.355 | 1.000 | 2506 | tags=32%, list=19%, signal=40% |
| 745 | WGGAATGY\_V$TEF1\_Q6 |  | 258 | 0.26 | 1.22 | 0.087 | 0.356 | 1.000 | 3321 | tags=31%, list=25%, signal=40% |
| 746 | V$ZIC3\_01 |  | 187 | 0.27 | 1.22 | 0.091 | 0.358 | 1.000 | 3420 | tags=29%, list=26%, signal=39% |
| 747 | LOTEM\_LEUKEMIA\_UP |  | 21 | 0.41 | 1.22 | 0.198 | 0.358 | 1.000 | 2292 | tags=33%, list=17%, signal=40% |
| 748 | HASLINGER\_B\_CLL\_17P13 |  | 16 | 0.44 | 1.22 | 0.222 | 0.357 | 1.000 | 2694 | tags=44%, list=20%, signal=55% |
| 749 | CHR6P25 |  | 23 | 0.40 | 1.22 | 0.198 | 0.358 | 1.000 | 2484 | tags=43%, list=19%, signal=53% |
| 750 | CCAATNNSNNNGCG\_UNKNOWN |  | 47 | 0.34 | 1.22 | 0.203 | 0.358 | 1.000 | 2965 | tags=36%, list=22%, signal=46% |
| 751 | OLD\_FIBRO\_UP |  | 55 | 0.33 | 1.22 | 0.175 | 0.358 | 1.000 | 2463 | tags=27%, list=18%, signal=33% |
| 752 | V$CREB\_Q4\_01 |  | 137 | 0.28 | 1.22 | 0.127 | 0.358 | 1.000 | 1206 | tags=16%, list=9%, signal=17% |
| 753 | CSKPATHWAY |  | 21 | 0.40 | 1.22 | 0.209 | 0.360 | 1.000 | 4156 | tags=43%, list=31%, signal=62% |
| 754 | V$HAND1E47\_01 |  | 173 | 0.27 | 1.22 | 0.100 | 0.360 | 1.000 | 2450 | tags=21%, list=18%, signal=26% |
| 755 | CMV\_HCMV\_TIMECOURSE\_18HRS\_UP |  | 74 | 0.31 | 1.22 | 0.153 | 0.360 | 1.000 | 2898 | tags=31%, list=22%, signal=40% |
| 756 | INFLAMMATORY\_RESPONSE\_PATHWAY |  | 29 | 0.37 | 1.21 | 0.203 | 0.362 | 1.000 | 3652 | tags=45%, list=27%, signal=62% |
| 757 | V$IPF1\_Q4 |  | 171 | 0.27 | 1.21 | 0.111 | 0.362 | 1.000 | 3927 | tags=37%, list=29%, signal=52% |
| 758 | CARDIACEGFPATHWAY |  | 17 | 0.43 | 1.21 | 0.219 | 0.361 | 1.000 | 1025 | tags=24%, list=8%, signal=25% |
| 759 | ACTGAAA,MIR-30A-3P,MIR-30E-3P |  | 155 | 0.28 | 1.21 | 0.098 | 0.362 | 1.000 | 2058 | tags=23%, list=15%, signal=26% |
| 760 | CHR18Q11 |  | 19 | 0.41 | 1.21 | 0.218 | 0.362 | 1.000 | 2275 | tags=32%, list=17%, signal=38% |
| 761 | V$NFY\_Q6\_01 |  | 184 | 0.27 | 1.21 | 0.100 | 0.365 | 1.000 | 3516 | tags=34%, list=26%, signal=46% |
| 762 | AMIPATHWAY |  | 21 | 0.40 | 1.21 | 0.204 | 0.365 | 1.000 | 4156 | tags=43%, list=31%, signal=62% |
| 763 | UVB\_NHEK4\_24HRS\_DN |  | 18 | 0.42 | 1.21 | 0.207 | 0.366 | 1.000 | 3076 | tags=33%, list=23%, signal=43% |
| 764 | IGF1\_NIH3T3\_UP |  | 34 | 0.35 | 1.21 | 0.193 | 0.367 | 1.000 | 3048 | tags=32%, list=23%, signal=42% |
| 765 | V$ETS2\_B |  | 191 | 0.27 | 1.21 | 0.107 | 0.366 | 1.000 | 3221 | tags=30%, list=24%, signal=39% |
| 766 | ALZHEIMERS\_DISEASE\_DN |  | 1116 | 0.24 | 1.21 | 0.012 | 0.367 | 1.000 | 2722 | tags=25%, list=20%, signal=29% |
| 767 | TSADAC\_RKOSILENT\_UP |  | 19 | 0.42 | 1.21 | 0.229 | 0.367 | 1.000 | 1435 | tags=32%, list=11%, signal=35% |
| 768 | ST\_GA12\_PATHWAY |  | 21 | 0.41 | 1.21 | 0.230 | 0.366 | 1.000 | 4005 | tags=38%, list=30%, signal=54% |
| 769 | AS3\_FIBRO\_UP |  | 43 | 0.34 | 1.21 | 0.191 | 0.368 | 1.000 | 3265 | tags=40%, list=25%, signal=52% |
| 770 | CMV\_HCMV\_TIMECOURSE\_14HRS\_DN |  | 41 | 0.35 | 1.21 | 0.171 | 0.369 | 1.000 | 1046 | tags=22%, list=8%, signal=24% |
| 771 | LH\_GRANULOSA\_UP |  | 80 | 0.31 | 1.21 | 0.164 | 0.369 | 1.000 | 2727 | tags=33%, list=20%, signal=41% |
| 772 | V$PAX8\_01 |  | 25 | 0.39 | 1.21 | 0.206 | 0.369 | 1.000 | 1436 | tags=20%, list=11%, signal=22% |
| 773 | CHR6Q22 |  | 38 | 0.35 | 1.21 | 0.191 | 0.369 | 1.000 | 2997 | tags=37%, list=22%, signal=47% |
| 774 | ZMPSTE24\_KO\_DN |  | 24 | 0.38 | 1.21 | 0.215 | 0.371 | 1.000 | 4182 | tags=50%, list=31%, signal=73% |
| 775 | V$P53\_DECAMER\_Q2 |  | 180 | 0.27 | 1.21 | 0.116 | 0.373 | 1.000 | 3588 | tags=34%, list=27%, signal=47% |
| 776 | TCATCTC,MIR-143 |  | 111 | 0.28 | 1.21 | 0.137 | 0.373 | 1.000 | 3294 | tags=32%, list=25%, signal=42% |
| 777 | GGGATGC,MIR-324-5P |  | 38 | 0.35 | 1.20 | 0.196 | 0.374 | 1.000 | 4717 | tags=55%, list=35%, signal=85% |
| 778 | V$TTF1\_Q6 |  | 182 | 0.27 | 1.20 | 0.111 | 0.376 | 1.000 | 2990 | tags=26%, list=22%, signal=34% |
| 779 | HOHENKIRK\_MONOCYTE\_DEND\_UP |  | 106 | 0.30 | 1.20 | 0.167 | 0.377 | 1.000 | 2795 | tags=30%, list=21%, signal=38% |
| 780 | YYCATTCAWW\_UNKNOWN |  | 141 | 0.28 | 1.20 | 0.129 | 0.378 | 1.000 | 3687 | tags=34%, list=28%, signal=47% |
| 781 | HSC\_EARLYPROGENITORS\_FETAL |  | 342 | 0.25 | 1.20 | 0.066 | 0.378 | 1.000 | 3907 | tags=37%, list=29%, signal=51% |
| 782 | TGCTTTG,MIR-330 |  | 245 | 0.26 | 1.20 | 0.090 | 0.379 | 1.000 | 2238 | tags=22%, list=17%, signal=26% |
| 783 | UVC\_HIGH\_D6\_DN |  | 31 | 0.36 | 1.20 | 0.218 | 0.381 | 1.000 | 718 | tags=19%, list=5%, signal=20% |
| 784 | HSC\_STHSC\_ADULT |  | 30 | 0.36 | 1.20 | 0.219 | 0.380 | 1.000 | 2135 | tags=33%, list=16%, signal=40% |
| 785 | GCACTTT,MIR-17-5P,MIR-20A,MIR-106A,MIR-106B,MIR-20B,MIR-519D |  | 442 | 0.25 | 1.20 | 0.061 | 0.381 | 1.000 | 2690 | tags=26%, list=20%, signal=32% |
| 786 | AGGGCAG,MIR-18A |  | 99 | 0.29 | 1.20 | 0.139 | 0.381 | 1.000 | 2226 | tags=22%, list=17%, signal=26% |
| 787 | ROSS\_CBF\_MYH |  | 54 | 0.33 | 1.20 | 0.192 | 0.381 | 1.000 | 2424 | tags=28%, list=18%, signal=34% |
| 788 | CCAGGTT,MIR-490 |  | 48 | 0.33 | 1.20 | 0.178 | 0.381 | 1.000 | 2200 | tags=25%, list=17%, signal=30% |
| 789 | TCTATGA,MIR-376A,MIR-376B |  | 68 | 0.31 | 1.20 | 0.176 | 0.381 | 1.000 | 3828 | tags=37%, list=29%, signal=51% |
| 790 | GATTGGY\_V$NFY\_Q6\_01 |  | 797 | 0.24 | 1.20 | 0.037 | 0.381 | 1.000 | 3669 | tags=31%, list=28%, signal=40% |
| 791 | V$NFKB\_C |  | 195 | 0.27 | 1.20 | 0.115 | 0.382 | 1.000 | 3129 | tags=30%, list=23%, signal=38% |
| 792 | V$MYCMAX\_03 |  | 185 | 0.27 | 1.20 | 0.116 | 0.381 | 1.000 | 3447 | tags=33%, list=26%, signal=44% |
| 793 | MOREAUX\_TACI\_HI\_VS\_LOW\_UP |  | 391 | 0.25 | 1.20 | 0.076 | 0.382 | 1.000 | 3505 | tags=30%, list=26%, signal=40% |
| 794 | MMS\_MOUSE\_LYMPH\_HIGH\_4HRS\_UP |  | 33 | 0.35 | 1.20 | 0.214 | 0.382 | 1.000 | 3382 | tags=39%, list=25%, signal=53% |
| 795 | CHR2P24 |  | 38 | 0.35 | 1.20 | 0.194 | 0.384 | 1.000 | 1623 | tags=24%, list=12%, signal=27% |
| 796 | V$CDPCR1\_01 |  | 88 | 0.29 | 1.20 | 0.153 | 0.384 | 1.000 | 1200 | tags=17%, list=9%, signal=19% |
| 797 | HSC\_EARLYPROGENITORS\_SHARED |  | 342 | 0.25 | 1.19 | 0.083 | 0.387 | 1.000 | 3907 | tags=37%, list=29%, signal=51% |
| 798 | CCAWWNAAGG\_V$SRF\_Q4 |  | 64 | 0.31 | 1.19 | 0.196 | 0.389 | 1.000 | 2653 | tags=28%, list=20%, signal=35% |
| 799 | SANA\_IFNG\_ENDOTHELIAL\_UP |  | 60 | 0.31 | 1.19 | 0.189 | 0.390 | 1.000 | 3137 | tags=33%, list=24%, signal=43% |
| 800 | TTANTCA\_UNKNOWN |  | 642 | 0.24 | 1.19 | 0.049 | 0.390 | 1.000 | 4133 | tags=34%, list=31%, signal=47% |
| 801 | V$OCT1\_07 |  | 101 | 0.29 | 1.19 | 0.160 | 0.394 | 1.000 | 3175 | tags=36%, list=24%, signal=46% |
| 802 | KUROKAWA\_5FU\_IFN\_SENSITIVE\_VS\_RESISTANT\_DN |  | 31 | 0.36 | 1.19 | 0.214 | 0.394 | 1.000 | 1448 | tags=26%, list=11%, signal=29% |
| 803 | TAATGTG,MIR-323 |  | 115 | 0.28 | 1.19 | 0.156 | 0.399 | 1.000 | 2656 | tags=27%, list=20%, signal=33% |
| 804 | CMV-UV\_HCMV\_6HRS\_DN |  | 107 | 0.29 | 1.19 | 0.166 | 0.399 | 1.000 | 3536 | tags=39%, list=27%, signal=53% |
| 805 | FSH\_GRANULOSA\_UP |  | 78 | 0.30 | 1.19 | 0.191 | 0.400 | 1.000 | 2727 | tags=32%, list=20%, signal=40% |
| 806 | AS3\_FIBRO\_C3 |  | 43 | 0.34 | 1.19 | 0.211 | 0.404 | 1.000 | 3265 | tags=40%, list=25%, signal=52% |
| 807 | V$ICSBP\_Q6 |  | 170 | 0.27 | 1.19 | 0.126 | 0.404 | 1.000 | 3457 | tags=34%, list=26%, signal=45% |
| 808 | V$MSX1\_01 |  | 130 | 0.28 | 1.19 | 0.161 | 0.404 | 1.000 | 2706 | tags=26%, list=20%, signal=33% |
| 809 | ROS\_MOUSE\_AORTA\_DN |  | 69 | 0.30 | 1.19 | 0.167 | 0.404 | 1.000 | 2103 | tags=29%, list=16%, signal=34% |
| 810 | JISON\_SICKLECELL\_DIFF |  | 367 | 0.25 | 1.18 | 0.106 | 0.405 | 1.000 | 3777 | tags=34%, list=28%, signal=46% |
| 811 | V$EGR3\_01 |  | 57 | 0.32 | 1.18 | 0.188 | 0.405 | 1.000 | 4095 | tags=42%, list=31%, signal=61% |
| 812 | TAKEDA\_NUP8\_HOXA9\_16D\_UP |  | 135 | 0.28 | 1.18 | 0.153 | 0.405 | 1.000 | 4361 | tags=39%, list=33%, signal=58% |
| 813 | LEI\_HOXC8\_DN |  | 15 | 0.43 | 1.18 | 0.247 | 0.405 | 1.000 | 1982 | tags=33%, list=15%, signal=39% |
| 814 | HSC\_EARLYPROGENITORS\_ADULT |  | 344 | 0.25 | 1.18 | 0.112 | 0.405 | 1.000 | 3907 | tags=37%, list=29%, signal=50% |
| 815 | V$NFKB\_Q6\_01 |  | 159 | 0.27 | 1.18 | 0.149 | 0.405 | 1.000 | 3599 | tags=34%, list=27%, signal=46% |
| 816 | CAGNWMCNNNGAC\_UNKNOWN |  | 64 | 0.31 | 1.18 | 0.196 | 0.404 | 1.000 | 5999 | tags=63%, list=45%, signal=113% |
| 817 | V$TFIIA\_Q6 |  | 187 | 0.27 | 1.18 | 0.135 | 0.404 | 1.000 | 2931 | tags=26%, list=22%, signal=32% |
| 818 | SMTTTTGT\_UNKNOWN |  | 301 | 0.25 | 1.18 | 0.102 | 0.405 | 1.000 | 3817 | tags=33%, list=29%, signal=45% |
| 819 | TENEDINI\_MEGAKARYOCYTIC\_GENES |  | 53 | 0.32 | 1.18 | 0.218 | 0.405 | 1.000 | 3540 | tags=42%, list=27%, signal=56% |
| 820 | V$BACH1\_01 |  | 181 | 0.27 | 1.18 | 0.122 | 0.408 | 1.000 | 3672 | tags=34%, list=28%, signal=46% |
| 821 | PARK\_MSCS\_DIFF |  | 28 | 0.36 | 1.18 | 0.233 | 0.410 | 1.000 | 2762 | tags=39%, list=21%, signal=49% |
| 822 | V$CEBPGAMMA\_Q6 |  | 171 | 0.26 | 1.18 | 0.158 | 0.412 | 1.000 | 3000 | tags=30%, list=23%, signal=38% |
| 823 | V$CREBP1\_Q2 |  | 174 | 0.26 | 1.18 | 0.151 | 0.412 | 1.000 | 3564 | tags=33%, list=27%, signal=44% |
| 824 | LINDSTEDT\_DEND\_8H\_VS\_48H\_DN |  | 67 | 0.30 | 1.18 | 0.199 | 0.413 | 1.000 | 3571 | tags=36%, list=27%, signal=49% |
| 825 | CTACCTC,LET-7A,LET-7B,LET-7C,LET-7D,LET-7E,LET-7F,MIR-98,LET-7G,LET-7I |  | 267 | 0.25 | 1.18 | 0.120 | 0.412 | 1.000 | 3540 | tags=32%, list=27%, signal=42% |
| 826 | LEE\_ACOX1\_DN |  | 63 | 0.31 | 1.18 | 0.188 | 0.413 | 1.000 | 2353 | tags=27%, list=18%, signal=33% |
| 827 | STRIATED\_MUSCLE\_CONTRACTION |  | 36 | 0.35 | 1.18 | 0.247 | 0.413 | 1.000 | 5007 | tags=47%, list=38%, signal=75% |
| 828 | RADMACHER\_AMLNORMALKARYTYPE\_SIG |  | 78 | 0.30 | 1.18 | 0.189 | 0.413 | 1.000 | 3965 | tags=44%, list=30%, signal=62% |
| 829 | ETSPATHWAY |  | 15 | 0.42 | 1.18 | 0.246 | 0.412 | 1.000 | 2414 | tags=33%, list=18%, signal=41% |
| 830 | GAMMA-UV\_FIBRO\_DN |  | 36 | 0.34 | 1.18 | 0.233 | 0.412 | 1.000 | 3302 | tags=33%, list=25%, signal=44% |
| 831 | FERRARI\_4HPR\_UP |  | 22 | 0.39 | 1.18 | 0.254 | 0.412 | 1.000 | 1202 | tags=32%, list=9%, signal=35% |
| 832 | CHR6Q23 |  | 29 | 0.36 | 1.18 | 0.236 | 0.413 | 1.000 | 2873 | tags=34%, list=22%, signal=44% |
| 833 | HUMAN\_TISSUE\_PANCREAS |  | 37 | 0.35 | 1.18 | 0.224 | 0.412 | 1.000 | 4382 | tags=43%, list=33%, signal=64% |
| 834 | CHR1Q25 |  | 50 | 0.32 | 1.18 | 0.215 | 0.414 | 1.000 | 3946 | tags=40%, list=30%, signal=57% |
| 835 | V$ARNT\_02 |  | 180 | 0.26 | 1.18 | 0.140 | 0.414 | 1.000 | 2787 | tags=27%, list=21%, signal=34% |
| 836 | FETAL\_LIVER\_ENRICHED\_TRANSCRIPTION\_FACTORS |  | 75 | 0.30 | 1.18 | 0.200 | 0.415 | 1.000 | 3354 | tags=37%, list=25%, signal=50% |
| 837 | ZHANG\_EFT\_EWSFLI1\_UP |  | 88 | 0.29 | 1.18 | 0.194 | 0.415 | 1.000 | 3169 | tags=35%, list=24%, signal=46% |
| 838 | HDACI\_COLON\_SUL\_DN |  | 182 | 0.26 | 1.18 | 0.134 | 0.415 | 1.000 | 3806 | tags=34%, list=29%, signal=46% |
| 839 | LEE\_TCELLS5\_UP |  | 17 | 0.42 | 1.17 | 0.257 | 0.414 | 1.000 | 1932 | tags=29%, list=15%, signal=34% |
| 840 | SA\_TRKA\_RECEPTOR |  | 16 | 0.42 | 1.17 | 0.264 | 0.414 | 1.000 | 2312 | tags=31%, list=17%, signal=38% |
| 841 | WERNER\_FIBRO\_DN |  | 153 | 0.27 | 1.17 | 0.140 | 0.414 | 1.000 | 3151 | tags=31%, list=24%, signal=41% |
| 842 | ZHAN\_MMPC\_SIMAL |  | 47 | 0.33 | 1.17 | 0.195 | 0.414 | 1.000 | 3279 | tags=43%, list=25%, signal=56% |
| 843 | V$GATA1\_04 |  | 179 | 0.26 | 1.17 | 0.152 | 0.416 | 1.000 | 3561 | tags=30%, list=27%, signal=40% |
| 844 | LVAD\_HEARTFAILURE\_UP |  | 89 | 0.29 | 1.17 | 0.189 | 0.418 | 1.000 | 2276 | tags=26%, list=17%, signal=31% |
| 845 | DORSAM\_HOXA9\_UP |  | 31 | 0.35 | 1.17 | 0.226 | 0.418 | 1.000 | 2118 | tags=32%, list=16%, signal=38% |
| 846 | V$AP2ALPHA\_01 |  | 177 | 0.26 | 1.17 | 0.150 | 0.418 | 1.000 | 3040 | tags=26%, list=23%, signal=33% |
| 847 | UV-4NQO\_FIBRO\_UP |  | 28 | 0.36 | 1.17 | 0.222 | 0.418 | 1.000 | 2074 | tags=32%, list=16%, signal=38% |
| 848 | CHR8Q22 |  | 55 | 0.31 | 1.17 | 0.209 | 0.420 | 1.000 | 4359 | tags=45%, list=33%, signal=67% |
| 849 | ZHAN\_MM\_MOLECULAR\_CLASSI\_DN |  | 41 | 0.33 | 1.17 | 0.220 | 0.421 | 1.000 | 3499 | tags=37%, list=26%, signal=49% |
| 850 | CHRXP21 |  | 17 | 0.40 | 1.17 | 0.275 | 0.421 | 1.000 | 4351 | tags=53%, list=33%, signal=79% |
| 851 | OXSTRESS\_RPETWO\_DN |  | 108 | 0.28 | 1.17 | 0.179 | 0.421 | 1.000 | 2594 | tags=28%, list=19%, signal=34% |
| 852 | HSC\_LATEPROGENITORS\_SHARED |  | 366 | 0.24 | 1.17 | 0.106 | 0.422 | 1.000 | 4267 | tags=41%, list=32%, signal=59% |
| 853 | RGAGGAARY\_V$PU1\_Q6 |  | 340 | 0.25 | 1.17 | 0.122 | 0.421 | 1.000 | 4340 | tags=39%, list=33%, signal=57% |
| 854 | SANA\_TNFA\_ENDOTHELIAL\_DN |  | 74 | 0.29 | 1.17 | 0.205 | 0.421 | 1.000 | 2395 | tags=24%, list=18%, signal=29% |
| 855 | CANTHARIDIN\_DN |  | 49 | 0.32 | 1.17 | 0.212 | 0.422 | 1.000 | 2760 | tags=31%, list=21%, signal=38% |
| 856 | GGCAGCT,MIR-22 |  | 177 | 0.26 | 1.17 | 0.158 | 0.422 | 1.000 | 4573 | tags=44%, list=34%, signal=65% |
| 857 | HSC\_LATEPROGENITORS\_ADULT |  | 373 | 0.24 | 1.17 | 0.107 | 0.421 | 1.000 | 4267 | tags=41%, list=32%, signal=58% |
| 858 | HSC\_HSCANDPROGENITORS\_FETAL |  | 452 | 0.24 | 1.17 | 0.100 | 0.423 | 1.000 | 3124 | tags=28%, list=23%, signal=35% |
| 859 | KUMAR\_HOXA\_DIFF |  | 313 | 0.25 | 1.17 | 0.125 | 0.422 | 1.000 | 2766 | tags=27%, list=21%, signal=33% |
| 860 | UVB\_NHEK2\_DN |  | 81 | 0.29 | 1.17 | 0.188 | 0.422 | 1.000 | 3031 | tags=37%, list=23%, signal=48% |
| 861 | V$NF1\_Q6\_01 |  | 169 | 0.26 | 1.17 | 0.152 | 0.422 | 1.000 | 2732 | tags=28%, list=21%, signal=35% |
| 862 | GH\_EXOGENOUS\_LATE\_DN |  | 70 | 0.30 | 1.17 | 0.190 | 0.422 | 1.000 | 2501 | tags=24%, list=19%, signal=30% |
| 863 | HDACI\_COLON\_BUT24HRS\_DN |  | 95 | 0.29 | 1.17 | 0.194 | 0.424 | 1.000 | 2533 | tags=32%, list=19%, signal=39% |
| 864 | V$CRX\_Q4 |  | 191 | 0.26 | 1.17 | 0.149 | 0.423 | 1.000 | 4133 | tags=40%, list=31%, signal=58% |
| 865 | CHR2P14 |  | 20 | 0.40 | 1.17 | 0.239 | 0.423 | 1.000 | 1605 | tags=25%, list=12%, signal=28% |
| 866 | TGCTGAY\_UNKNOWN |  | 370 | 0.24 | 1.17 | 0.104 | 0.423 | 1.000 | 3686 | tags=31%, list=28%, signal=41% |
| 867 | YNTTTNNNANGCARM\_UNKNOWN |  | 46 | 0.32 | 1.17 | 0.230 | 0.424 | 1.000 | 3119 | tags=26%, list=23%, signal=34% |
| 868 | APOPTOSIS\_KEGG |  | 49 | 0.32 | 1.17 | 0.227 | 0.424 | 1.000 | 2645 | tags=31%, list=20%, signal=38% |
| 869 | V$MAZ\_Q6 |  | 131 | 0.27 | 1.16 | 0.173 | 0.426 | 1.000 | 4654 | tags=46%, list=35%, signal=70% |
| 870 | V$CREB\_Q2\_01 |  | 150 | 0.27 | 1.16 | 0.170 | 0.427 | 1.000 | 3588 | tags=32%, list=27%, signal=43% |
| 871 | BCNU\_GLIOMA\_MGMT\_24HRS\_DN |  | 32 | 0.35 | 1.16 | 0.239 | 0.429 | 1.000 | 3212 | tags=41%, list=24%, signal=53% |
| 872 | ABRAHAM\_MM\_VS\_AL\_UP |  | 19 | 0.40 | 1.16 | 0.278 | 0.429 | 1.000 | 2918 | tags=37%, list=22%, signal=47% |
| 873 | CTCTGGA,MIR-520A,MIR-525 |  | 121 | 0.28 | 1.16 | 0.181 | 0.429 | 1.000 | 1665 | tags=18%, list=12%, signal=21% |
| 874 | RNGTGGGC\_UNKNOWN |  | 529 | 0.23 | 1.16 | 0.081 | 0.429 | 1.000 | 2274 | tags=21%, list=17%, signal=25% |
| 875 | UV-CMV\_UNIQUE\_HCMV\_6HRS\_DN |  | 81 | 0.30 | 1.16 | 0.218 | 0.429 | 1.000 | 3255 | tags=37%, list=24%, signal=49% |
| 876 | CAGTATT,MIR-200B,MIR-200C,MIR-429 |  | 364 | 0.24 | 1.16 | 0.124 | 0.429 | 1.000 | 2019 | tags=20%, list=15%, signal=22% |
| 877 | HIPPOCAMPUS\_DEVELOPMENT\_POSTNATAL |  | 44 | 0.32 | 1.16 | 0.221 | 0.429 | 1.000 | 2049 | tags=27%, list=15%, signal=32% |
| 878 | ABRAHAM\_AL\_VS\_MM\_DN |  | 18 | 0.40 | 1.16 | 0.279 | 0.431 | 1.000 | 2918 | tags=39%, list=22%, signal=50% |
| 879 | ACTACCT,MIR-196A,MIR-196B |  | 97 | 0.28 | 1.16 | 0.199 | 0.432 | 1.000 | 4484 | tags=47%, list=34%, signal=71% |
| 880 | UBIQUITIN\_MEDIATED\_PROTEOLYSIS |  | 22 | 0.38 | 1.16 | 0.259 | 0.432 | 1.000 | 1309 | tags=18%, list=10%, signal=20% |
| 881 | HSC\_HSCANDPROGENITORS\_SHARED |  | 452 | 0.24 | 1.16 | 0.114 | 0.434 | 1.000 | 3124 | tags=28%, list=23%, signal=35% |
| 882 | ADIP\_HUMAN\_UP |  | 59 | 0.31 | 1.16 | 0.222 | 0.434 | 1.000 | 2206 | tags=25%, list=17%, signal=30% |
| 883 | V$PAX4\_04 |  | 150 | 0.27 | 1.16 | 0.178 | 0.433 | 1.000 | 3465 | tags=35%, list=26%, signal=46% |
| 884 | V$NFY\_C |  | 176 | 0.26 | 1.16 | 0.178 | 0.433 | 1.000 | 3817 | tags=32%, list=29%, signal=44% |
| 885 | HSC\_LATEPROGENITORS\_FETAL |  | 373 | 0.24 | 1.16 | 0.127 | 0.433 | 1.000 | 2792 | tags=27%, list=21%, signal=34% |
| 886 | AAGTCCA,MIR-422B,MIR-422A |  | 48 | 0.32 | 1.16 | 0.230 | 0.433 | 1.000 | 3036 | tags=33%, list=23%, signal=43% |
| 887 | MENSE\_HYPOXIA\_UP |  | 90 | 0.28 | 1.16 | 0.205 | 0.433 | 1.000 | 2927 | tags=30%, list=22%, signal=38% |
| 888 | V$OCT\_Q6 |  | 173 | 0.26 | 1.16 | 0.179 | 0.433 | 1.000 | 3668 | tags=32%, list=28%, signal=44% |
| 889 | ZHAN\_MM\_MOLECULAR\_CLASSI\_UP |  | 47 | 0.33 | 1.16 | 0.222 | 0.433 | 1.000 | 1855 | tags=28%, list=14%, signal=32% |
| 890 | ZHAN\_MM\_CD138\_MS\_VS\_REST |  | 41 | 0.33 | 1.16 | 0.248 | 0.433 | 1.000 | 1068 | tags=20%, list=8%, signal=21% |
| 891 | V$GATA1\_05 |  | 196 | 0.25 | 1.16 | 0.149 | 0.435 | 1.000 | 2690 | tags=23%, list=20%, signal=29% |
| 892 | PARK\_MSCS\_LIN2 |  | 38 | 0.34 | 1.16 | 0.252 | 0.435 | 1.000 | 2621 | tags=34%, list=20%, signal=42% |
| 893 | V$CREBP1\_01 |  | 119 | 0.27 | 1.16 | 0.193 | 0.435 | 1.000 | 2450 | tags=22%, list=18%, signal=27% |
| 894 | V$TAL1ALPHAE47\_01 |  | 167 | 0.26 | 1.16 | 0.196 | 0.436 | 1.000 | 4281 | tags=42%, list=32%, signal=61% |
| 895 | V$OCT1\_01 |  | 178 | 0.26 | 1.15 | 0.176 | 0.437 | 1.000 | 1411 | tags=16%, list=11%, signal=18% |
| 896 | V$COMP1\_01 |  | 84 | 0.29 | 1.15 | 0.227 | 0.437 | 1.000 | 3805 | tags=40%, list=29%, signal=56% |
| 897 | LEE\_E2F1\_UP |  | 60 | 0.30 | 1.15 | 0.223 | 0.437 | 1.000 | 1419 | tags=23%, list=11%, signal=26% |
| 898 | CAGTGTT,MIR-141,MIR-200A |  | 245 | 0.25 | 1.15 | 0.126 | 0.439 | 1.000 | 3209 | tags=29%, list=24%, signal=38% |
| 899 | GGCAGTG,MIR-324-3P |  | 75 | 0.30 | 1.15 | 0.199 | 0.441 | 1.000 | 3146 | tags=29%, list=24%, signal=38% |
| 900 | SHIPP\_DLBCL\_CURED\_DN |  | 36 | 0.34 | 1.15 | 0.249 | 0.441 | 1.000 | 2915 | tags=39%, list=22%, signal=50% |
| 901 | CAMPTOTHECIN\_PROBCELL\_UP |  | 17 | 0.40 | 1.15 | 0.272 | 0.444 | 1.000 | 2492 | tags=41%, list=19%, signal=51% |
| 902 | HEMATOPOESIS\_RELATED\_TRANSCRIPTION\_FACTORS |  | 83 | 0.29 | 1.15 | 0.220 | 0.446 | 1.000 | 2762 | tags=28%, list=21%, signal=35% |
| 903 | V$OCT1\_B |  | 182 | 0.26 | 1.15 | 0.166 | 0.446 | 1.000 | 2813 | tags=25%, list=21%, signal=31% |
| 904 | V$EVI1\_05 |  | 117 | 0.27 | 1.15 | 0.208 | 0.445 | 1.000 | 2542 | tags=25%, list=19%, signal=30% |
| 905 | H2O2\_CSBDIFF\_C2 |  | 33 | 0.34 | 1.15 | 0.254 | 0.445 | 1.000 | 4138 | tags=42%, list=31%, signal=61% |
| 906 | YCATTAA\_UNKNOWN |  | 379 | 0.24 | 1.15 | 0.138 | 0.444 | 1.000 | 4017 | tags=34%, list=30%, signal=47% |
| 907 | HYPOPHYSECTOMY\_RAT\_UP |  | 33 | 0.34 | 1.15 | 0.261 | 0.444 | 1.000 | 2865 | tags=36%, list=22%, signal=46% |
| 908 | TAKEDA\_NUP8\_HOXA9\_10D\_UP |  | 152 | 0.26 | 1.15 | 0.190 | 0.445 | 1.000 | 2577 | tags=24%, list=19%, signal=30% |
| 909 | TARTE\_BCELL |  | 38 | 0.33 | 1.15 | 0.258 | 0.445 | 1.000 | 3034 | tags=34%, list=23%, signal=44% |
| 910 | GCCNNNWTAAR\_UNKNOWN |  | 104 | 0.28 | 1.15 | 0.219 | 0.445 | 1.000 | 3796 | tags=38%, list=28%, signal=53% |
| 911 | BYSTROM\_IL5\_DN |  | 56 | 0.30 | 1.15 | 0.234 | 0.446 | 1.000 | 3754 | tags=39%, list=28%, signal=54% |
| 912 | HYPERTROPHY\_MODEL |  | 20 | 0.39 | 1.15 | 0.281 | 0.446 | 1.000 | 65 | tags=10%, list=0%, signal=10% |
| 913 | RTTTNNNYTGGM\_UNKNOWN |  | 114 | 0.27 | 1.15 | 0.208 | 0.446 | 1.000 | 2182 | tags=23%, list=16%, signal=27% |
| 914 | V$CHX10\_01 |  | 154 | 0.26 | 1.15 | 0.198 | 0.448 | 1.000 | 4154 | tags=37%, list=31%, signal=53% |
| 915 | TPA\_RESIST\_EARLY\_DN |  | 74 | 0.29 | 1.15 | 0.222 | 0.448 | 1.000 | 3397 | tags=34%, list=26%, signal=45% |
| 916 | PARK\_MSCS\_BOTH |  | 41 | 0.33 | 1.15 | 0.257 | 0.448 | 1.000 | 3450 | tags=39%, list=26%, signal=53% |
| 917 | CTCTATG,MIR-368 |  | 31 | 0.35 | 1.15 | 0.261 | 0.449 | 1.000 | 3339 | tags=39%, list=25%, signal=52% |
| 918 | V$FOXJ2\_02 |  | 160 | 0.26 | 1.15 | 0.180 | 0.449 | 1.000 | 2813 | tags=24%, list=21%, signal=30% |
| 919 | CHESLER\_BRAIN\_HIGHEST\_VARIANCE\_GENES |  | 21 | 0.38 | 1.14 | 0.255 | 0.449 | 1.000 | 1815 | tags=29%, list=14%, signal=33% |
| 920 | NTHIPATHWAY |  | 21 | 0.38 | 1.14 | 0.266 | 0.452 | 1.000 | 2143 | tags=29%, list=16%, signal=34% |
| 921 | P53PATHWAY |  | 16 | 0.41 | 1.14 | 0.303 | 0.453 | 1.000 | 2688 | tags=38%, list=20%, signal=47% |
| 922 | UVB\_NHEK3\_C2 |  | 43 | 0.32 | 1.14 | 0.255 | 0.453 | 1.000 | 3642 | tags=40%, list=27%, signal=54% |
| 923 | PRMT5\_KD\_DN |  | 23 | 0.37 | 1.14 | 0.275 | 0.454 | 1.000 | 2627 | tags=26%, list=20%, signal=32% |
| 924 | KANG\_TERT\_UP |  | 79 | 0.29 | 1.14 | 0.231 | 0.454 | 1.000 | 3877 | tags=33%, list=29%, signal=46% |
| 925 | V$MEF2\_03 |  | 172 | 0.25 | 1.14 | 0.202 | 0.455 | 1.000 | 4340 | tags=39%, list=33%, signal=57% |
| 926 | ATAAGCT,MIR-21 |  | 90 | 0.28 | 1.14 | 0.245 | 0.457 | 1.000 | 2912 | tags=32%, list=22%, signal=41% |
| 927 | FASPATHWAY |  | 27 | 0.35 | 1.14 | 0.258 | 0.459 | 1.000 | 3098 | tags=44%, list=23%, signal=58% |
| 928 | DISTECHE\_XINACTIVATED\_GENES |  | 19 | 0.39 | 1.14 | 0.294 | 0.459 | 1.000 | 757 | tags=16%, list=6%, signal=17% |
| 929 | INSULIN\_ADIP\_INSENS\_UP |  | 20 | 0.39 | 1.14 | 0.296 | 0.459 | 1.000 | 2113 | tags=30%, list=16%, signal=36% |
| 930 | OLDWERNER\_FIBRO\_DN |  | 101 | 0.28 | 1.14 | 0.220 | 0.460 | 1.000 | 2653 | tags=27%, list=20%, signal=33% |
| 931 | GTTATAT,MIR-410 |  | 75 | 0.29 | 1.14 | 0.257 | 0.462 | 1.000 | 4061 | tags=40%, list=30%, signal=57% |
| 932 | TGACCTY\_V$ERR1\_Q2 |  | 713 | 0.23 | 1.14 | 0.107 | 0.463 | 1.000 | 3072 | tags=25%, list=23%, signal=31% |
| 933 | TAAWWATAG\_V$RSRFC4\_Q2 |  | 111 | 0.27 | 1.14 | 0.256 | 0.464 | 1.000 | 4281 | tags=41%, list=32%, signal=61% |
| 934 | HYPOXIA\_REG\_UP |  | 38 | 0.33 | 1.14 | 0.283 | 0.464 | 1.000 | 1648 | tags=29%, list=12%, signal=33% |
| 935 | SMALL\_LIGAND\_GPCRS |  | 17 | 0.41 | 1.14 | 0.318 | 0.463 | 1.000 | 4026 | tags=41%, list=30%, signal=59% |
| 936 | PASSERINI\_IMMUNE |  | 21 | 0.37 | 1.13 | 0.283 | 0.467 | 1.000 | 4285 | tags=52%, list=32%, signal=77% |
| 937 | ACATATC,MIR-190 |  | 44 | 0.32 | 1.13 | 0.254 | 0.468 | 1.000 | 2650 | tags=30%, list=20%, signal=37% |
| 938 | GGCACAT,MIR-455 |  | 42 | 0.32 | 1.13 | 0.270 | 0.468 | 1.000 | 2100 | tags=24%, list=16%, signal=28% |
| 939 | V$POU3F2\_02 |  | 180 | 0.25 | 1.13 | 0.208 | 0.468 | 1.000 | 3330 | tags=32%, list=25%, signal=42% |
| 940 | V$NFKAPPAB65\_01 |  | 168 | 0.26 | 1.13 | 0.201 | 0.469 | 1.000 | 3610 | tags=33%, list=27%, signal=44% |
| 941 | V$OCT1\_Q6 |  | 176 | 0.25 | 1.13 | 0.204 | 0.470 | 1.000 | 2954 | tags=24%, list=22%, signal=30% |
| 942 | WNT\_SIGNALING |  | 58 | 0.30 | 1.13 | 0.260 | 0.470 | 1.000 | 4209 | tags=47%, list=32%, signal=68% |
| 943 | UVB\_NHEK3\_C5 |  | 35 | 0.34 | 1.13 | 0.271 | 0.470 | 1.000 | 4617 | tags=51%, list=35%, signal=79% |
| 944 | V$ATF\_01 |  | 185 | 0.25 | 1.13 | 0.213 | 0.471 | 1.000 | 3486 | tags=32%, list=26%, signal=43% |
| 945 | TGACCTTG\_V$SF1\_Q6 |  | 171 | 0.26 | 1.13 | 0.204 | 0.471 | 1.000 | 3855 | tags=36%, list=29%, signal=50% |
| 946 | HSC\_HSCANDPROGENITORS\_ADULT |  | 459 | 0.23 | 1.13 | 0.155 | 0.472 | 1.000 | 3124 | tags=28%, list=23%, signal=35% |
| 947 | IRS\_KO\_ADIP\_UP |  | 23 | 0.37 | 1.13 | 0.270 | 0.473 | 1.000 | 2067 | tags=35%, list=16%, signal=41% |
| 948 | V$E4BP4\_01 |  | 154 | 0.26 | 1.13 | 0.211 | 0.473 | 1.000 | 4159 | tags=38%, list=31%, signal=54% |
| 949 | CHR16Q23 |  | 20 | 0.38 | 1.13 | 0.291 | 0.473 | 1.000 | 447 | tags=15%, list=3%, signal=15% |
| 950 | WGTTNNNNNAAA\_UNKNOWN |  | 383 | 0.23 | 1.13 | 0.167 | 0.476 | 1.000 | 3561 | tags=30%, list=27%, signal=39% |
| 951 | DFOSB\_BRAIN\_2WKS\_UP |  | 34 | 0.34 | 1.13 | 0.310 | 0.477 | 1.000 | 3272 | tags=32%, list=25%, signal=43% |
| 952 | V$TAXCREB\_01 |  | 102 | 0.27 | 1.13 | 0.230 | 0.478 | 1.000 | 1761 | tags=19%, list=13%, signal=21% |
| 953 | ET743\_HELA\_UP |  | 56 | 0.30 | 1.13 | 0.263 | 0.481 | 1.000 | 1668 | tags=27%, list=13%, signal=30% |
| 954 | CHR1P31 |  | 55 | 0.30 | 1.13 | 0.273 | 0.482 | 1.000 | 2025 | tags=20%, list=15%, signal=23% |
| 955 | GTGACTT,MIR-224 |  | 121 | 0.27 | 1.13 | 0.242 | 0.481 | 1.000 | 4082 | tags=39%, list=31%, signal=55% |
| 956 | UVB\_NHEK3\_C0 |  | 82 | 0.28 | 1.13 | 0.254 | 0.481 | 1.000 | 2500 | tags=28%, list=19%, signal=34% |
| 957 | V$SREBP\_Q3 |  | 167 | 0.25 | 1.13 | 0.212 | 0.481 | 1.000 | 3425 | tags=33%, list=26%, signal=44% |
| 958 | V$MZF1\_01 |  | 164 | 0.25 | 1.12 | 0.203 | 0.482 | 1.000 | 3833 | tags=35%, list=29%, signal=48% |
| 959 | CELL\_GROWTH\_AND\_OR\_MAINTENANCE |  | 58 | 0.30 | 1.12 | 0.280 | 0.483 | 1.000 | 2815 | tags=28%, list=21%, signal=35% |
| 960 | ANDROGEN\_AND\_ESTROGEN\_METABOLISM |  | 24 | 0.36 | 1.12 | 0.301 | 0.483 | 1.000 | 5226 | tags=67%, list=39%, signal=110% |
| 961 | ROTH\_HTERT\_DIFF |  | 31 | 0.34 | 1.12 | 0.303 | 0.482 | 1.000 | 3642 | tags=39%, list=27%, signal=53% |
| 962 | TESTIS\_EXPRESSED\_GENES |  | 61 | 0.29 | 1.12 | 0.273 | 0.482 | 1.000 | 2040 | tags=21%, list=15%, signal=25% |
| 963 | V$AML\_Q6 |  | 179 | 0.25 | 1.12 | 0.233 | 0.482 | 1.000 | 3747 | tags=34%, list=28%, signal=47% |
| 964 | TMTCGCGANR\_UNKNOWN |  | 95 | 0.27 | 1.12 | 0.236 | 0.482 | 1.000 | 1562 | tags=18%, list=12%, signal=20% |
| 965 | AGCACTT,MIR-93,MIR-302A,MIR-302B,MIR-302C,MIR-302D,MIR-372,MIR-373,MIR-520E,MIR-520A,MIR-526B,MIR-520B,MIR-520C,MIR-520D |  | 242 | 0.24 | 1.12 | 0.190 | 0.482 | 1.000 | 3105 | tags=29%, list=23%, signal=37% |
| 966 | CCAWYNNGAAR\_UNKNOWN |  | 94 | 0.27 | 1.12 | 0.240 | 0.484 | 1.000 | 3125 | tags=31%, list=23%, signal=40% |
| 967 | LIZUKA\_G2\_GR\_G3 |  | 26 | 0.35 | 1.12 | 0.289 | 0.484 | 1.000 | 4146 | tags=46%, list=31%, signal=67% |
| 968 | IL7PATHWAY |  | 16 | 0.41 | 1.12 | 0.313 | 0.483 | 1.000 | 3330 | tags=44%, list=25%, signal=58% |
| 969 | PDGF\_ES\_UP |  | 17 | 0.39 | 1.12 | 0.315 | 0.483 | 1.000 | 1493 | tags=29%, list=11%, signal=33% |
| 970 | V$TAL1BETAITF2\_01 |  | 164 | 0.25 | 1.12 | 0.223 | 0.483 | 1.000 | 4281 | tags=39%, list=32%, signal=57% |
| 971 | TTGTTT\_V$FOXO4\_01 |  | 1393 | 0.22 | 1.12 | 0.075 | 0.483 | 1.000 | 3459 | tags=29%, list=26%, signal=35% |
| 972 | V$DR1\_Q3 |  | 170 | 0.25 | 1.12 | 0.237 | 0.483 | 1.000 | 4324 | tags=35%, list=32%, signal=52% |
| 973 | YAGI\_AML\_PROG\_ASSOC |  | 125 | 0.26 | 1.12 | 0.248 | 0.483 | 1.000 | 2713 | tags=28%, list=20%, signal=35% |
| 974 | S1P\_SIGNALING |  | 24 | 0.37 | 1.12 | 0.291 | 0.483 | 1.000 | 4304 | tags=54%, list=32%, signal=80% |
| 975 | GTTAAAG,MIR-302B |  | 52 | 0.31 | 1.12 | 0.287 | 0.483 | 1.000 | 3043 | tags=31%, list=23%, signal=40% |
| 976 | TGTTTAC,MIR-30A-5P,MIR-30C,MIR-30D,MIR-30B,MIR-30E-5P |  | 428 | 0.23 | 1.12 | 0.168 | 0.483 | 1.000 | 3628 | tags=32%, list=27%, signal=43% |
| 977 | V$EVI1\_03 |  | 42 | 0.31 | 1.12 | 0.288 | 0.484 | 1.000 | 3321 | tags=33%, list=25%, signal=44% |
| 978 | V$HLF\_01 |  | 182 | 0.25 | 1.12 | 0.228 | 0.485 | 1.000 | 3782 | tags=36%, list=28%, signal=50% |
| 979 | STANELLE\_E2F1\_UP |  | 28 | 0.35 | 1.12 | 0.300 | 0.485 | 1.000 | 1583 | tags=25%, list=12%, signal=28% |
| 980 | LIZUKA\_L0\_SM\_L1 |  | 19 | 0.39 | 1.12 | 0.294 | 0.486 | 1.000 | 2599 | tags=42%, list=20%, signal=52% |
| 981 | CHEN\_HOXA5\_TARGETS\_UP |  | 229 | 0.24 | 1.12 | 0.217 | 0.486 | 1.000 | 2609 | tags=24%, list=20%, signal=29% |
| 982 | V$TAL1BETAE47\_01 |  | 171 | 0.25 | 1.12 | 0.250 | 0.486 | 1.000 | 3404 | tags=32%, list=26%, signal=42% |
| 983 | V$OCT\_C |  | 184 | 0.25 | 1.12 | 0.222 | 0.486 | 1.000 | 4305 | tags=38%, list=32%, signal=55% |
| 984 | KREBS\_TCA\_CYCLE |  | 29 | 0.35 | 1.12 | 0.301 | 0.486 | 1.000 | 3331 | tags=38%, list=25%, signal=50% |
| 985 | V$AP1\_Q2\_01 |  | 189 | 0.25 | 1.12 | 0.241 | 0.487 | 1.000 | 4143 | tags=37%, list=31%, signal=53% |
| 986 | CHR6P24 |  | 17 | 0.39 | 1.12 | 0.302 | 0.486 | 1.000 | 3435 | tags=53%, list=26%, signal=71% |
| 987 | TGCACTG,MIR-148A,MIR-152,MIR-148B |  | 219 | 0.24 | 1.12 | 0.205 | 0.487 | 1.000 | 2424 | tags=23%, list=18%, signal=27% |
| 988 | INTEGRINPATHWAY |  | 32 | 0.33 | 1.12 | 0.293 | 0.487 | 1.000 | 3460 | tags=31%, list=26%, signal=42% |
| 989 | BASSO\_GERMINAL\_CENTER\_CD40\_DN |  | 69 | 0.29 | 1.12 | 0.278 | 0.487 | 1.000 | 4319 | tags=48%, list=32%, signal=70% |
| 990 | V$FOXJ2\_01 |  | 142 | 0.26 | 1.12 | 0.241 | 0.487 | 1.000 | 3406 | tags=31%, list=26%, signal=41% |
| 991 | ET743\_SARCOMA\_24HRS\_DN |  | 95 | 0.27 | 1.12 | 0.259 | 0.486 | 1.000 | 2568 | tags=28%, list=19%, signal=35% |
| 992 | TCTGATC,MIR-383 |  | 36 | 0.33 | 1.12 | 0.304 | 0.486 | 1.000 | 1779 | tags=25%, list=13%, signal=29% |
| 993 | V$TATA\_01 |  | 196 | 0.25 | 1.12 | 0.211 | 0.487 | 1.000 | 3632 | tags=30%, list=27%, signal=41% |
| 994 | CORTEX\_ENRICHMENT\_LATE\_UP |  | 19 | 0.38 | 1.12 | 0.313 | 0.487 | 1.000 | 1008 | tags=26%, list=8%, signal=28% |
| 995 | CHR10Q11 |  | 39 | 0.32 | 1.11 | 0.310 | 0.489 | 1.000 | 1598 | tags=21%, list=12%, signal=23% |
| 996 | REOVIRUS\_HEK293\_DN |  | 230 | 0.24 | 1.11 | 0.222 | 0.490 | 1.000 | 2649 | tags=27%, list=20%, signal=34% |
| 997 | HOFFMANN\_BIVSBII\_IMVM |  | 75 | 0.28 | 1.11 | 0.255 | 0.492 | 1.000 | 2506 | tags=24%, list=19%, signal=29% |
| 998 | PHOSPHATIDYLINOSITOL\_SIGNALING\_SYSTEM |  | 88 | 0.27 | 1.11 | 0.275 | 0.495 | 1.000 | 2517 | tags=24%, list=19%, signal=29% |
| 999 | SPPAPATHWAY |  | 20 | 0.37 | 1.11 | 0.314 | 0.500 | 1.000 | 1859 | tags=25%, list=14%, signal=29% |
| 1000 | CARBON\_FIXATION |  | 20 | 0.37 | 1.11 | 0.339 | 0.500 | 1.000 | 1698 | tags=30%, list=13%, signal=34% |
| 1001 | SMOOTH\_MUSCLE\_CONTRACTION |  | 133 | 0.26 | 1.11 | 0.252 | 0.500 | 1.000 | 3757 | tags=35%, list=28%, signal=49% |
| 1002 | ST\_MYOCYTE\_AD\_PATHWAY |  | 24 | 0.36 | 1.11 | 0.326 | 0.500 | 1.000 | 3852 | tags=42%, list=29%, signal=59% |
| 1003 | CTCAAGA,MIR-526B |  | 50 | 0.31 | 1.11 | 0.304 | 0.500 | 1.000 | 2023 | tags=24%, list=15%, signal=28% |
| 1004 | LIAN\_MYELOID\_DIFF\_TF |  | 34 | 0.33 | 1.11 | 0.308 | 0.502 | 1.000 | 1025 | tags=21%, list=8%, signal=22% |
| 1005 | VEGFPATHWAY |  | 27 | 0.34 | 1.11 | 0.305 | 0.501 | 1.000 | 4563 | tags=44%, list=34%, signal=67% |
| 1006 | AACTTT\_UNKNOWN |  | 1333 | 0.21 | 1.11 | 0.109 | 0.505 | 1.000 | 3193 | tags=26%, list=24%, signal=31% |
| 1007 | V$NFE2\_01 |  | 200 | 0.24 | 1.11 | 0.228 | 0.505 | 1.000 | 2786 | tags=28%, list=21%, signal=35% |
| 1008 | CACTGTG,MIR-128A,MIR-128B |  | 244 | 0.24 | 1.11 | 0.236 | 0.505 | 1.000 | 3872 | tags=36%, list=29%, signal=50% |
| 1009 | BRG1\_SW13\_UP |  | 43 | 0.31 | 1.11 | 0.301 | 0.504 | 1.000 | 2565 | tags=30%, list=19%, signal=37% |
| 1010 | V$AP3\_Q6 |  | 174 | 0.25 | 1.11 | 0.269 | 0.506 | 1.000 | 3403 | tags=30%, list=26%, signal=40% |
| 1011 | GUO\_HEX\_UP |  | 76 | 0.28 | 1.11 | 0.281 | 0.506 | 1.000 | 2467 | tags=25%, list=19%, signal=31% |
| 1012 | COCAINE\_BRAIN\_4WKS\_UP |  | 55 | 0.30 | 1.10 | 0.308 | 0.509 | 1.000 | 2485 | tags=25%, list=19%, signal=31% |
| 1013 | APPEL\_IMATINIB\_UP |  | 31 | 0.34 | 1.10 | 0.302 | 0.509 | 1.000 | 1002 | tags=16%, list=8%, signal=17% |
| 1014 | V$AREB6\_01 |  | 173 | 0.25 | 1.10 | 0.250 | 0.512 | 1.000 | 2474 | tags=23%, list=19%, signal=28% |
| 1015 | AGGAAGC,MIR-516-3P |  | 88 | 0.27 | 1.10 | 0.274 | 0.512 | 1.000 | 3608 | tags=32%, list=27%, signal=43% |
| 1016 | VERHAAK\_AML\_NPM1\_MUT\_VS\_WT\_DN |  | 258 | 0.24 | 1.10 | 0.232 | 0.512 | 1.000 | 2803 | tags=27%, list=21%, signal=34% |
| 1017 | V$IRF1\_Q6 |  | 179 | 0.25 | 1.10 | 0.251 | 0.513 | 1.000 | 3348 | tags=31%, list=25%, signal=41% |
| 1018 | V$PITX2\_Q2 |  | 176 | 0.25 | 1.10 | 0.252 | 0.514 | 1.000 | 2975 | tags=26%, list=22%, signal=32% |
| 1019 | V$STAT6\_01 |  | 179 | 0.25 | 1.10 | 0.261 | 0.514 | 1.000 | 2521 | tags=22%, list=19%, signal=27% |
| 1020 | V$SRF\_Q6 |  | 191 | 0.25 | 1.10 | 0.260 | 0.515 | 1.000 | 3212 | tags=29%, list=24%, signal=37% |
| 1021 | DAVIES\_MGUS\_MM |  | 35 | 0.33 | 1.10 | 0.313 | 0.516 | 1.000 | 4489 | tags=49%, list=34%, signal=73% |
| 1022 | GGCCAGT,MIR-193A,MIR-193B |  | 73 | 0.28 | 1.10 | 0.279 | 0.516 | 1.000 | 3775 | tags=37%, list=28%, signal=51% |
| 1023 | UVB\_SCC\_UP |  | 86 | 0.28 | 1.10 | 0.297 | 0.517 | 1.000 | 2452 | tags=27%, list=18%, signal=33% |
| 1024 | V$MYCMAX\_B |  | 189 | 0.25 | 1.10 | 0.258 | 0.517 | 1.000 | 3969 | tags=38%, list=30%, signal=53% |
| 1025 | LEE\_TCELLS6\_UP |  | 21 | 0.36 | 1.10 | 0.323 | 0.519 | 1.000 | 2479 | tags=29%, list=19%, signal=35% |
| 1026 | DCPATHWAY |  | 20 | 0.37 | 1.10 | 0.334 | 0.519 | 1.000 | 5157 | tags=45%, list=39%, signal=73% |
| 1027 | TAATTA\_V$CHX10\_01 |  | 542 | 0.22 | 1.10 | 0.194 | 0.520 | 1.000 | 2756 | tags=23%, list=21%, signal=28% |
| 1028 | V$EN1\_01 |  | 80 | 0.28 | 1.10 | 0.315 | 0.521 | 1.000 | 4196 | tags=41%, list=31%, signal=60% |
| 1029 | TGACATY\_UNKNOWN |  | 448 | 0.23 | 1.10 | 0.220 | 0.522 | 1.000 | 3153 | tags=25%, list=24%, signal=32% |
| 1030 | PEART\_HISTONE\_UP |  | 51 | 0.30 | 1.10 | 0.311 | 0.523 | 1.000 | 1827 | tags=24%, list=14%, signal=27% |
| 1031 | CROMER\_HYPOPHARYNGEAL\_MET\_VS\_NON\_UP |  | 71 | 0.28 | 1.10 | 0.305 | 0.523 | 1.000 | 3091 | tags=39%, list=23%, signal=51% |
| 1032 | RYTTCCTG\_V$ETS2\_B |  | 756 | 0.22 | 1.09 | 0.171 | 0.524 | 1.000 | 4188 | tags=36%, list=31%, signal=49% |
| 1033 | CHR7Q35 |  | 19 | 0.37 | 1.09 | 0.350 | 0.524 | 1.000 | 764 | tags=16%, list=6%, signal=17% |
| 1034 | AGEING\_LYMPH\_DN |  | 17 | 0.39 | 1.09 | 0.355 | 0.526 | 1.000 | 2926 | tags=47%, list=22%, signal=60% |
| 1035 | TARTE\_PC |  | 80 | 0.28 | 1.09 | 0.281 | 0.527 | 1.000 | 2351 | tags=29%, list=18%, signal=35% |
| 1036 | DRUG\_RESISTANCE\_AND\_METABOLISM |  | 95 | 0.27 | 1.09 | 0.316 | 0.529 | 1.000 | 2804 | tags=29%, list=21%, signal=37% |
| 1037 | LEE\_MYC\_E2F1\_UP |  | 55 | 0.30 | 1.09 | 0.309 | 0.529 | 1.000 | 2834 | tags=35%, list=21%, signal=44% |
| 1038 | V$ZIC1\_01 |  | 185 | 0.25 | 1.09 | 0.271 | 0.529 | 1.000 | 3078 | tags=28%, list=23%, signal=35% |
| 1039 | V$FREAC7\_01 |  | 139 | 0.26 | 1.09 | 0.272 | 0.530 | 1.000 | 3423 | tags=33%, list=26%, signal=44% |
| 1040 | FLOTHO\_CASP8AP2\_MRD\_DIFF |  | 84 | 0.27 | 1.09 | 0.323 | 0.530 | 1.000 | 3639 | tags=35%, list=27%, signal=47% |
| 1041 | V$TAXCREB\_02 |  | 19 | 0.38 | 1.09 | 0.326 | 0.531 | 1.000 | 1435 | tags=21%, list=11%, signal=24% |
| 1042 | NOVA2\_KO\_SPLICING |  | 41 | 0.32 | 1.09 | 0.329 | 0.531 | 1.000 | 3494 | tags=32%, list=26%, signal=43% |
| 1043 | IFNALPHA\_HCC\_UP |  | 29 | 0.33 | 1.09 | 0.346 | 0.531 | 1.000 | 1849 | tags=24%, list=14%, signal=28% |
| 1044 | BRENTANI\_TRANSCRIPTION\_FACTORS |  | 64 | 0.28 | 1.09 | 0.319 | 0.532 | 1.000 | 2576 | tags=31%, list=19%, signal=39% |
| 1045 | CHR9P22 |  | 27 | 0.34 | 1.09 | 0.329 | 0.531 | 1.000 | 4109 | tags=37%, list=31%, signal=53% |
| 1046 | HASLINGER\_B\_CLL\_11Q23 |  | 20 | 0.36 | 1.09 | 0.347 | 0.531 | 1.000 | 3276 | tags=40%, list=25%, signal=53% |
| 1047 | NITROGEN\_METABOLISM |  | 21 | 0.36 | 1.09 | 0.327 | 0.533 | 1.000 | 909 | tags=19%, list=7%, signal=20% |
| 1048 | GACTGTT,MIR-212,MIR-132 |  | 118 | 0.26 | 1.09 | 0.299 | 0.537 | 1.000 | 3460 | tags=31%, list=26%, signal=42% |
| 1049 | V$SP3\_Q3 |  | 165 | 0.25 | 1.09 | 0.292 | 0.537 | 1.000 | 2992 | tags=28%, list=22%, signal=36% |
| 1050 | V$GATA\_Q6 |  | 147 | 0.25 | 1.09 | 0.306 | 0.537 | 1.000 | 2221 | tags=20%, list=17%, signal=24% |
| 1051 | V$AMEF2\_Q6 |  | 184 | 0.24 | 1.09 | 0.289 | 0.539 | 1.000 | 2874 | tags=26%, list=22%, signal=33% |
| 1052 | UVB\_NHEK1\_C1 |  | 51 | 0.29 | 1.08 | 0.332 | 0.543 | 1.000 | 4094 | tags=45%, list=31%, signal=65% |
| 1053 | TRANSLATION\_FACTORS |  | 45 | 0.31 | 1.08 | 0.353 | 0.543 | 1.000 | 2812 | tags=27%, list=21%, signal=34% |
| 1054 | SA\_B\_CELL\_RECEPTOR\_COMPLEXES |  | 24 | 0.36 | 1.08 | 0.323 | 0.545 | 1.000 | 1859 | tags=33%, list=14%, signal=39% |
| 1055 | ESR\_FIBROBLAST\_UP |  | 50 | 0.30 | 1.08 | 0.339 | 0.545 | 1.000 | 3229 | tags=34%, list=24%, signal=45% |
| 1056 | V$CEBP\_01 |  | 187 | 0.24 | 1.08 | 0.291 | 0.545 | 1.000 | 4482 | tags=38%, list=34%, signal=56% |
| 1057 | GCAAAAA,MIR-129 |  | 142 | 0.25 | 1.08 | 0.321 | 0.545 | 1.000 | 3743 | tags=35%, list=28%, signal=48% |
| 1058 | LEE\_MYC\_TGFA\_DN |  | 63 | 0.29 | 1.08 | 0.324 | 0.547 | 1.000 | 4418 | tags=35%, list=33%, signal=52% |
| 1059 | CCACACA,MIR-147 |  | 53 | 0.29 | 1.08 | 0.334 | 0.548 | 1.000 | 2399 | tags=23%, list=18%, signal=28% |
| 1060 | IL2PATHWAY |  | 22 | 0.35 | 1.08 | 0.345 | 0.548 | 1.000 | 2391 | tags=32%, list=18%, signal=39% |
| 1061 | V$MYOD\_Q6\_01 |  | 165 | 0.24 | 1.08 | 0.295 | 0.548 | 1.000 | 3926 | tags=35%, list=29%, signal=48% |
| 1062 | V$CDX2\_Q5 |  | 181 | 0.24 | 1.08 | 0.285 | 0.551 | 1.000 | 3149 | tags=28%, list=24%, signal=36% |
| 1063 | CATTTCA,MIR-203 |  | 215 | 0.23 | 1.08 | 0.285 | 0.551 | 1.000 | 1774 | tags=19%, list=13%, signal=21% |
| 1064 | MEF2DPATHWAY |  | 18 | 0.37 | 1.08 | 0.366 | 0.551 | 1.000 | 720 | tags=17%, list=5%, signal=18% |
| 1065 | HEARTFAILURE\_ATRIA\_DN |  | 110 | 0.26 | 1.08 | 0.313 | 0.552 | 1.000 | 3085 | tags=33%, list=23%, signal=42% |
| 1066 | LEE\_MYC\_UP |  | 53 | 0.30 | 1.08 | 0.343 | 0.554 | 1.000 | 2993 | tags=32%, list=22%, signal=41% |
| 1067 | V$E47\_01 |  | 170 | 0.24 | 1.08 | 0.297 | 0.555 | 1.000 | 2992 | tags=29%, list=22%, signal=37% |
| 1068 | V$NKX61\_01 |  | 161 | 0.25 | 1.08 | 0.312 | 0.555 | 1.000 | 3337 | tags=30%, list=25%, signal=39% |
| 1069 | CXCR4PATHWAY |  | 23 | 0.35 | 1.08 | 0.357 | 0.555 | 1.000 | 3460 | tags=35%, list=26%, signal=47% |
| 1070 | RYTAAWNNNTGAY\_UNKNOWN |  | 45 | 0.30 | 1.08 | 0.374 | 0.555 | 1.000 | 4709 | tags=56%, list=35%, signal=86% |
| 1071 | V$NFAT\_Q6 |  | 169 | 0.24 | 1.08 | 0.310 | 0.556 | 1.000 | 3610 | tags=33%, list=27%, signal=45% |
| 1072 | CTTTGCA,MIR-527 |  | 191 | 0.24 | 1.07 | 0.304 | 0.557 | 1.000 | 2665 | tags=24%, list=20%, signal=29% |
| 1073 | PEPTIDE\_GPCRS |  | 70 | 0.28 | 1.07 | 0.313 | 0.560 | 1.000 | 4781 | tags=43%, list=36%, signal=66% |
| 1074 | BYSTROM\_IL5\_UP |  | 39 | 0.31 | 1.07 | 0.351 | 0.560 | 1.000 | 2646 | tags=31%, list=20%, signal=38% |
| 1075 | HUMAN\_MITODB\_6\_2002 |  | 379 | 0.22 | 1.07 | 0.288 | 0.560 | 1.000 | 2905 | tags=25%, list=22%, signal=31% |
| 1076 | CCCAGAG,MIR-326 |  | 117 | 0.25 | 1.07 | 0.311 | 0.561 | 1.000 | 3936 | tags=37%, list=30%, signal=52% |
| 1077 | GH\_GHRHR\_KO\_24HRS\_DN |  | 141 | 0.25 | 1.07 | 0.292 | 0.561 | 1.000 | 2647 | tags=26%, list=20%, signal=32% |
| 1078 | V$ER\_Q6\_02 |  | 179 | 0.24 | 1.07 | 0.311 | 0.561 | 1.000 | 2884 | tags=27%, list=22%, signal=34% |
| 1079 | AGEING\_KIDNEY\_DN |  | 110 | 0.26 | 1.07 | 0.328 | 0.561 | 1.000 | 2583 | tags=27%, list=19%, signal=34% |
| 1080 | ATCATGA,MIR-433 |  | 84 | 0.27 | 1.07 | 0.306 | 0.561 | 1.000 | 2984 | tags=27%, list=22%, signal=35% |
| 1081 | ATGTTAA,MIR-302C |  | 177 | 0.24 | 1.07 | 0.313 | 0.561 | 1.000 | 2019 | tags=19%, list=15%, signal=22% |
| 1082 | V$ELK1\_02 |  | 160 | 0.24 | 1.07 | 0.313 | 0.561 | 1.000 | 2256 | tags=23%, list=17%, signal=27% |
| 1083 | CHR13Q12 |  | 51 | 0.29 | 1.07 | 0.334 | 0.561 | 1.000 | 1486 | tags=20%, list=11%, signal=22% |
| 1084 | IDX\_TSA\_DN\_CLUSTER2 |  | 56 | 0.29 | 1.07 | 0.327 | 0.562 | 1.000 | 3775 | tags=41%, list=28%, signal=57% |
| 1085 | YGTCCTTGR\_UNKNOWN |  | 69 | 0.28 | 1.07 | 0.352 | 0.562 | 1.000 | 2727 | tags=28%, list=20%, signal=34% |
| 1086 | CAAGGAT,MIR-362 |  | 46 | 0.30 | 1.07 | 0.362 | 0.563 | 1.000 | 3936 | tags=39%, list=30%, signal=55% |
| 1087 | TGCCAAR\_V$NF1\_Q6 |  | 474 | 0.22 | 1.07 | 0.278 | 0.564 | 1.000 | 2746 | tags=25%, list=21%, signal=31% |
| 1088 | V$NKX62\_Q2 |  | 160 | 0.24 | 1.07 | 0.309 | 0.564 | 1.000 | 2758 | tags=27%, list=21%, signal=33% |
| 1089 | V$AREB6\_02 |  | 175 | 0.24 | 1.07 | 0.309 | 0.564 | 1.000 | 2616 | tags=25%, list=20%, signal=30% |
| 1090 | MARTINELLI\_IFNS\_DIFF |  | 21 | 0.35 | 1.07 | 0.349 | 0.566 | 1.000 | 2718 | tags=38%, list=20%, signal=48% |
| 1091 | CHRXQ25 |  | 15 | 0.39 | 1.07 | 0.375 | 0.567 | 1.000 | 5309 | tags=60%, list=40%, signal=100% |
| 1092 | CHR9Q31 |  | 32 | 0.32 | 1.07 | 0.373 | 0.567 | 1.000 | 2393 | tags=31%, list=18%, signal=38% |
| 1093 | BRENTANI\_IMMUNE\_FUNCTION |  | 50 | 0.29 | 1.07 | 0.341 | 0.567 | 1.000 | 4240 | tags=44%, list=32%, signal=64% |
| 1094 | MMS\_MOUSE\_LYMPH\_HIGH\_24HRS\_UP |  | 22 | 0.35 | 1.07 | 0.373 | 0.567 | 1.000 | 3139 | tags=41%, list=24%, signal=53% |
| 1095 | EDG1PATHWAY |  | 25 | 0.34 | 1.07 | 0.381 | 0.569 | 1.000 | 5054 | tags=52%, list=38%, signal=84% |
| 1096 | RCGCANGCGY\_V$NRF1\_Q6 |  | 582 | 0.21 | 1.06 | 0.267 | 0.574 | 1.000 | 2846 | tags=25%, list=21%, signal=30% |
| 1097 | V$USF\_C |  | 193 | 0.23 | 1.06 | 0.316 | 0.574 | 1.000 | 1290 | tags=15%, list=10%, signal=16% |
| 1098 | AGED\_MOUSE\_HIPPOCAMPUS\_ANY\_DN |  | 39 | 0.31 | 1.06 | 0.367 | 0.575 | 1.000 | 1594 | tags=26%, list=12%, signal=29% |
| 1099 | CHR22Q11 |  | 110 | 0.25 | 1.06 | 0.353 | 0.575 | 1.000 | 5628 | tags=48%, list=42%, signal=83% |
| 1100 | CALCINEURINPATHWAY |  | 18 | 0.36 | 1.06 | 0.373 | 0.575 | 1.000 | 720 | tags=17%, list=5%, signal=18% |
| 1101 | CPR\_NULL-LOW\_LIVER\_UP |  | 17 | 0.38 | 1.06 | 0.376 | 0.575 | 1.000 | 3801 | tags=53%, list=29%, signal=74% |
| 1102 | ATCMNTCCGY\_UNKNOWN |  | 35 | 0.32 | 1.06 | 0.372 | 0.576 | 1.000 | 2941 | tags=31%, list=22%, signal=40% |
| 1103 | V$AP1\_Q6\_01 |  | 187 | 0.24 | 1.06 | 0.321 | 0.577 | 1.000 | 3610 | tags=33%, list=27%, signal=45% |
| 1104 | FLECHNER\_KIDNEY\_TRANSPLANT\_WELL\_DN |  | 22 | 0.35 | 1.06 | 0.386 | 0.576 | 1.000 | 1359 | tags=23%, list=10%, signal=25% |
| 1105 | V$FOXO4\_01 |  | 161 | 0.24 | 1.06 | 0.336 | 0.578 | 1.000 | 3283 | tags=32%, list=25%, signal=42% |
| 1106 | TSADAC\_HYPOMETH\_OVCA\_UP |  | 39 | 0.30 | 1.06 | 0.379 | 0.578 | 1.000 | 1541 | tags=21%, list=12%, signal=23% |
| 1107 | TAKEDA\_NUP8\_HOXA9\_6H\_UP |  | 68 | 0.27 | 1.06 | 0.339 | 0.578 | 1.000 | 2762 | tags=24%, list=21%, signal=30% |
| 1108 | UVB\_NHEK1\_C2 |  | 21 | 0.35 | 1.06 | 0.380 | 0.578 | 1.000 | 668 | tags=14%, list=5%, signal=15% |
| 1109 | YTATTTTNR\_V$MEF2\_02 |  | 476 | 0.22 | 1.06 | 0.300 | 0.579 | 1.000 | 3410 | tags=29%, list=26%, signal=37% |
| 1110 | V$SF1\_Q6 |  | 175 | 0.24 | 1.06 | 0.344 | 0.581 | 1.000 | 4164 | tags=34%, list=31%, signal=48% |
| 1111 | CROONQUIST\_IL6\_STROMA\_UP |  | 37 | 0.30 | 1.06 | 0.369 | 0.581 | 1.000 | 2590 | tags=35%, list=19%, signal=43% |
| 1112 | MATRIX\_METALLOPROTEINASES |  | 29 | 0.33 | 1.06 | 0.380 | 0.583 | 1.000 | 2540 | tags=28%, list=19%, signal=34% |
| 1113 | PROSTAGLANDIN\_AND\_LEUKOTRIENE\_METABOLISM |  | 32 | 0.32 | 1.06 | 0.375 | 0.585 | 1.000 | 4925 | tags=50%, list=37%, signal=79% |
| 1114 | JNK\_UP |  | 28 | 0.33 | 1.06 | 0.379 | 0.586 | 1.000 | 2506 | tags=36%, list=19%, signal=44% |
| 1115 | DFOSB\_BRAIN\_8WKS\_UP |  | 36 | 0.31 | 1.06 | 0.372 | 0.586 | 1.000 | 2512 | tags=31%, list=19%, signal=38% |
| 1116 | RAC1PATHWAY |  | 22 | 0.35 | 1.06 | 0.368 | 0.586 | 1.000 | 4563 | tags=55%, list=34%, signal=83% |
| 1117 | MENSE\_HYPOXIA\_TRANSPORTER\_GENES |  | 37 | 0.31 | 1.06 | 0.383 | 0.587 | 1.000 | 3591 | tags=43%, list=27%, signal=59% |
| 1118 | UVB\_NHEK1\_UP |  | 173 | 0.24 | 1.06 | 0.332 | 0.587 | 1.000 | 3740 | tags=32%, list=28%, signal=44% |
| 1119 | RYAAAKNNNNNNTTGW\_UNKNOWN |  | 62 | 0.28 | 1.05 | 0.381 | 0.588 | 1.000 | 2579 | tags=23%, list=19%, signal=28% |
| 1120 | RNTCANNRNNYNATTW\_UNKNOWN |  | 41 | 0.30 | 1.05 | 0.358 | 0.588 | 1.000 | 1820 | tags=17%, list=14%, signal=20% |
| 1121 | GTCTTCC,MIR-7 |  | 129 | 0.24 | 1.05 | 0.355 | 0.589 | 1.000 | 3117 | tags=26%, list=23%, signal=34% |
| 1122 | TGAYRTCA\_V$ATF3\_Q6 |  | 366 | 0.22 | 1.05 | 0.317 | 0.589 | 1.000 | 3486 | tags=27%, list=26%, signal=36% |
| 1123 | GCATTTG,MIR-105 |  | 129 | 0.25 | 1.05 | 0.363 | 0.590 | 1.000 | 2569 | tags=24%, list=19%, signal=29% |
| 1124 | OLD\_FIBRO\_DN |  | 147 | 0.24 | 1.05 | 0.360 | 0.590 | 1.000 | 2653 | tags=25%, list=20%, signal=31% |
| 1125 | V$PAX8\_B |  | 70 | 0.27 | 1.05 | 0.381 | 0.590 | 1.000 | 3306 | tags=31%, list=25%, signal=42% |
| 1126 | TAKEDA\_NUP8\_HOXA9\_3D\_UP |  | 150 | 0.24 | 1.05 | 0.360 | 0.592 | 1.000 | 4851 | tags=43%, list=36%, signal=66% |
| 1127 | V$P53\_02 |  | 183 | 0.24 | 1.05 | 0.345 | 0.592 | 1.000 | 3805 | tags=33%, list=29%, signal=45% |
| 1128 | CHR6P22 |  | 80 | 0.27 | 1.05 | 0.336 | 0.593 | 1.000 | 3796 | tags=31%, list=28%, signal=43% |
| 1129 | POD1\_KO\_MOST\_DN |  | 21 | 0.36 | 1.05 | 0.403 | 0.593 | 1.000 | 2935 | tags=33%, list=22%, signal=43% |
| 1130 | CHR2Q33 |  | 53 | 0.29 | 1.05 | 0.363 | 0.592 | 1.000 | 3541 | tags=34%, list=27%, signal=46% |
| 1131 | MOREAUX\_TACI\_HI\_IN\_BMPC |  | 94 | 0.26 | 1.05 | 0.369 | 0.592 | 1.000 | 2961 | tags=28%, list=22%, signal=35% |
| 1132 | UVC\_HIGH\_D5\_DN |  | 36 | 0.31 | 1.05 | 0.390 | 0.591 | 1.000 | 2493 | tags=28%, list=19%, signal=34% |
| 1133 | V$RP58\_01 |  | 154 | 0.24 | 1.05 | 0.364 | 0.592 | 1.000 | 5065 | tags=46%, list=38%, signal=74% |
| 1134 | CHR4Q13 |  | 34 | 0.31 | 1.05 | 0.376 | 0.593 | 1.000 | 5735 | tags=47%, list=43%, signal=82% |
| 1135 | V$XBP1\_01 |  | 101 | 0.26 | 1.05 | 0.377 | 0.593 | 1.000 | 3410 | tags=30%, list=26%, signal=40% |
| 1136 | CHR7Q36 |  | 34 | 0.31 | 1.05 | 0.377 | 0.595 | 1.000 | 4022 | tags=32%, list=30%, signal=46% |
| 1137 | V$ZIC2\_01 |  | 171 | 0.23 | 1.05 | 0.352 | 0.595 | 1.000 | 2777 | tags=26%, list=21%, signal=32% |
| 1138 | N\_GLYCAN\_BIOSYNTHESIS |  | 22 | 0.34 | 1.05 | 0.394 | 0.596 | 1.000 | 2291 | tags=32%, list=17%, signal=38% |
| 1139 | AGEING\_BRAIN\_UP |  | 207 | 0.23 | 1.05 | 0.346 | 0.595 | 1.000 | 1861 | tags=18%, list=14%, signal=21% |
| 1140 | GAGACTG,MIR-452 |  | 74 | 0.27 | 1.05 | 0.382 | 0.596 | 1.000 | 1523 | tags=18%, list=11%, signal=20% |
| 1141 | MOOTHA\_VOXPHOS |  | 77 | 0.27 | 1.05 | 0.375 | 0.596 | 1.000 | 1394 | tags=18%, list=10%, signal=20% |
| 1142 | PARP\_KO\_UP |  | 30 | 0.32 | 1.05 | 0.398 | 0.596 | 1.000 | 1346 | tags=23%, list=10%, signal=26% |
| 1143 | ZHAN\_MM\_CD138\_CD1\_VS\_REST |  | 33 | 0.31 | 1.05 | 0.405 | 0.596 | 1.000 | 4182 | tags=45%, list=31%, signal=66% |
| 1144 | ADIP\_DIFF\_CLUSTER2 |  | 38 | 0.30 | 1.05 | 0.411 | 0.596 | 1.000 | 1474 | tags=24%, list=11%, signal=27% |
| 1145 | V$SRF\_Q4 |  | 179 | 0.23 | 1.05 | 0.359 | 0.596 | 1.000 | 3212 | tags=31%, list=24%, signal=40% |
| 1146 | GPCRDB\_CLASS\_A\_RHODOPSIN\_LIKE |  | 160 | 0.24 | 1.05 | 0.364 | 0.598 | 1.000 | 5384 | tags=48%, list=40%, signal=79% |
| 1147 | MAMMARY\_DEV\_UP |  | 50 | 0.29 | 1.05 | 0.406 | 0.599 | 1.000 | 3573 | tags=36%, list=27%, signal=49% |
| 1148 | V$MAF\_Q6 |  | 185 | 0.23 | 1.04 | 0.367 | 0.603 | 1.000 | 3926 | tags=36%, list=29%, signal=50% |
| 1149 | V$E12\_Q6 |  | 168 | 0.24 | 1.04 | 0.364 | 0.602 | 1.000 | 2992 | tags=27%, list=22%, signal=34% |
| 1150 | CTGTTAC,MIR-194 |  | 74 | 0.26 | 1.04 | 0.387 | 0.602 | 1.000 | 3998 | tags=38%, list=30%, signal=54% |
| 1151 | IL6\_SCAR\_FIBRO\_UP |  | 24 | 0.34 | 1.04 | 0.409 | 0.602 | 1.000 | 1206 | tags=25%, list=9%, signal=27% |
| 1152 | YWATTWNNRGCT\_UNKNOWN |  | 47 | 0.29 | 1.04 | 0.397 | 0.602 | 1.000 | 4467 | tags=40%, list=34%, signal=61% |
| 1153 | V$AP1\_Q4\_01 |  | 186 | 0.23 | 1.04 | 0.370 | 0.605 | 1.000 | 3875 | tags=33%, list=29%, signal=46% |
| 1154 | CELL\_ADHESION |  | 167 | 0.24 | 1.04 | 0.367 | 0.605 | 1.000 | 2106 | tags=19%, list=16%, signal=22% |
| 1155 | V$EVI1\_02 |  | 84 | 0.26 | 1.04 | 0.390 | 0.607 | 1.000 | 3374 | tags=31%, list=25%, signal=41% |
| 1156 | V$OSF2\_Q6 |  | 180 | 0.23 | 1.04 | 0.383 | 0.608 | 1.000 | 4096 | tags=33%, list=31%, signal=47% |
| 1157 | WWTAAGGC\_UNKNOWN |  | 99 | 0.25 | 1.04 | 0.383 | 0.608 | 1.000 | 2813 | tags=29%, list=21%, signal=37% |
| 1158 | ERM\_KO\_SERTOLI\_DN |  | 18 | 0.36 | 1.04 | 0.419 | 0.611 | 1.000 | 2665 | tags=39%, list=20%, signal=49% |
| 1159 | TGCGCANK\_UNKNOWN |  | 359 | 0.22 | 1.04 | 0.377 | 0.611 | 1.000 | 3434 | tags=29%, list=26%, signal=38% |
| 1160 | V$ATF3\_Q6 |  | 177 | 0.23 | 1.04 | 0.370 | 0.611 | 1.000 | 3040 | tags=26%, list=23%, signal=33% |
| 1161 | CHR6P12 |  | 29 | 0.32 | 1.04 | 0.412 | 0.612 | 1.000 | 1908 | tags=24%, list=14%, signal=28% |
| 1162 | GH\_GHRHR\_KO\_6HRS\_DN |  | 30 | 0.32 | 1.04 | 0.389 | 0.612 | 1.000 | 3886 | tags=47%, list=29%, signal=66% |
| 1163 | V$EVI1\_06 |  | 18 | 0.36 | 1.04 | 0.387 | 0.611 | 1.000 | 2224 | tags=33%, list=17%, signal=40% |
| 1164 | V$AR\_Q2 |  | 85 | 0.26 | 1.04 | 0.394 | 0.612 | 1.000 | 3374 | tags=28%, list=25%, signal=38% |
| 1165 | CHR16Q22 |  | 93 | 0.26 | 1.04 | 0.409 | 0.614 | 1.000 | 2717 | tags=27%, list=20%, signal=34% |
| 1166 | KMCATNNWGGA\_UNKNOWN |  | 61 | 0.27 | 1.04 | 0.387 | 0.614 | 1.000 | 3263 | tags=28%, list=24%, signal=37% |
| 1167 | KLEIN\_PEL\_UP |  | 50 | 0.28 | 1.04 | 0.389 | 0.615 | 1.000 | 5398 | tags=58%, list=41%, signal=97% |
| 1168 | TPA\_RESIST\_LATE\_DN |  | 63 | 0.27 | 1.04 | 0.415 | 0.616 | 1.000 | 3323 | tags=37%, list=25%, signal=48% |
| 1169 | V$MEIS1AHOXA9\_01 |  | 79 | 0.26 | 1.03 | 0.420 | 0.620 | 1.000 | 2453 | tags=22%, list=18%, signal=26% |
| 1170 | TAANNYSGCG\_UNKNOWN |  | 64 | 0.27 | 1.03 | 0.396 | 0.621 | 1.000 | 1339 | tags=17%, list=10%, signal=19% |
| 1171 | V$FOXM1\_01 |  | 169 | 0.23 | 1.03 | 0.407 | 0.624 | 1.000 | 1289 | tags=14%, list=10%, signal=16% |
| 1172 | V$AP1\_Q6 |  | 189 | 0.23 | 1.03 | 0.386 | 0.624 | 1.000 | 4198 | tags=39%, list=32%, signal=56% |
| 1173 | HTERT\_UP |  | 58 | 0.27 | 1.03 | 0.407 | 0.624 | 1.000 | 1567 | tags=19%, list=12%, signal=21% |
| 1174 | V$ZF5\_01 |  | 169 | 0.23 | 1.03 | 0.392 | 0.624 | 1.000 | 3969 | tags=36%, list=30%, signal=51% |
| 1175 | CHR3Q22 |  | 28 | 0.32 | 1.03 | 0.418 | 0.625 | 1.000 | 5371 | tags=54%, list=40%, signal=90% |
| 1176 | HADDAD\_HSC\_CD10\_UP |  | 296 | 0.22 | 1.03 | 0.378 | 0.626 | 1.000 | 3170 | tags=28%, list=24%, signal=36% |
| 1177 | HOFMANN\_MDS\_CD34\_LOW\_RISK |  | 50 | 0.29 | 1.03 | 0.404 | 0.626 | 1.000 | 3110 | tags=38%, list=23%, signal=49% |
| 1178 | V$CREB\_Q2 |  | 182 | 0.23 | 1.03 | 0.402 | 0.628 | 1.000 | 3588 | tags=32%, list=27%, signal=44% |
| 1179 | V$STAT5A\_03 |  | 184 | 0.23 | 1.03 | 0.395 | 0.629 | 1.000 | 4417 | tags=38%, list=33%, signal=56% |
| 1180 | V$HNF6\_Q6 |  | 168 | 0.23 | 1.03 | 0.395 | 0.633 | 1.000 | 3384 | tags=30%, list=25%, signal=39% |
| 1181 | YATGNWAAT\_V$OCT\_C |  | 248 | 0.22 | 1.03 | 0.400 | 0.635 | 1.000 | 3321 | tags=28%, list=25%, signal=36% |
| 1182 | V$GATA1\_03 |  | 179 | 0.23 | 1.03 | 0.396 | 0.635 | 1.000 | 3591 | tags=30%, list=27%, signal=40% |
| 1183 | V$HNF4ALPHA\_Q6 |  | 182 | 0.23 | 1.03 | 0.389 | 0.635 | 1.000 | 2295 | tags=20%, list=17%, signal=24% |
| 1184 | MPRPATHWAY |  | 22 | 0.34 | 1.03 | 0.430 | 0.635 | 1.000 | 3460 | tags=32%, list=26%, signal=43% |
| 1185 | UVC\_LOW\_C2\_DN |  | 20 | 0.35 | 1.02 | 0.432 | 0.636 | 1.000 | 2800 | tags=40%, list=21%, signal=51% |
| 1186 | RORIE\_ES\_PNET\_DN |  | 27 | 0.32 | 1.02 | 0.431 | 0.636 | 1.000 | 2410 | tags=26%, list=18%, signal=32% |
| 1187 | NADLER\_OBESITY\_UP |  | 50 | 0.28 | 1.02 | 0.406 | 0.637 | 1.000 | 3564 | tags=38%, list=27%, signal=52% |
| 1188 | V$TGIF\_01 |  | 164 | 0.23 | 1.02 | 0.415 | 0.637 | 1.000 | 1758 | tags=19%, list=13%, signal=22% |
| 1189 | SANSOM\_APC\_5\_DN |  | 300 | 0.22 | 1.02 | 0.407 | 0.638 | 1.000 | 4199 | tags=37%, list=32%, signal=53% |
| 1190 | V$OCT1\_Q5\_01 |  | 180 | 0.23 | 1.02 | 0.420 | 0.638 | 1.000 | 4274 | tags=33%, list=32%, signal=48% |
| 1191 | HTERT\_DN |  | 61 | 0.27 | 1.02 | 0.426 | 0.639 | 1.000 | 1556 | tags=18%, list=12%, signal=20% |
| 1192 | HDACI\_COLON\_BUT48HRS\_UP |  | 84 | 0.25 | 1.02 | 0.406 | 0.640 | 1.000 | 3109 | tags=32%, list=23%, signal=42% |
| 1193 | HOFMANN\_MANTEL\_LYMPHOMA\_VS\_LYMPH\_NODES\_DN |  | 39 | 0.29 | 1.02 | 0.433 | 0.641 | 1.000 | 2134 | tags=18%, list=16%, signal=21% |
| 1194 | V$CREBP1CJUN\_01 |  | 181 | 0.23 | 1.02 | 0.406 | 0.643 | 1.000 | 3486 | tags=29%, list=26%, signal=38% |
| 1195 | HDACI\_COLON\_CLUSTER5 |  | 21 | 0.34 | 1.02 | 0.424 | 0.643 | 1.000 | 1517 | tags=29%, list=11%, signal=32% |
| 1196 | ATGCTGG,MIR-338 |  | 82 | 0.25 | 1.02 | 0.432 | 0.644 | 1.000 | 1880 | tags=20%, list=14%, signal=23% |
| 1197 | 4NQO\_ESR\_WS\_UNREG |  | 33 | 0.30 | 1.02 | 0.431 | 0.645 | 1.000 | 2795 | tags=30%, list=21%, signal=38% |
| 1198 | V$ZID\_01 |  | 168 | 0.23 | 1.02 | 0.420 | 0.644 | 1.000 | 4310 | tags=42%, list=32%, signal=61% |
| 1199 | TARTE\_MATURE\_PC |  | 382 | 0.21 | 1.02 | 0.405 | 0.644 | 1.000 | 4278 | tags=37%, list=32%, signal=53% |
| 1200 | TGCAAAC,MIR-452 |  | 78 | 0.25 | 1.02 | 0.422 | 0.645 | 1.000 | 1834 | tags=22%, list=14%, signal=25% |
| 1201 | CGCTGCT,MIR-503 |  | 16 | 0.37 | 1.02 | 0.440 | 0.645 | 1.000 | 3596 | tags=44%, list=27%, signal=60% |
| 1202 | ZHAN\_MMPC\_LATEVS |  | 45 | 0.28 | 1.02 | 0.401 | 0.646 | 1.000 | 2594 | tags=31%, list=19%, signal=39% |
| 1203 | ATGAAGG,MIR-205 |  | 123 | 0.24 | 1.02 | 0.405 | 0.652 | 1.000 | 2413 | tags=22%, list=18%, signal=27% |
| 1204 | V$ER\_Q6\_01 |  | 183 | 0.23 | 1.02 | 0.429 | 0.652 | 1.000 | 3148 | tags=28%, list=24%, signal=37% |
| 1205 | V$COREBINDINGFACTOR\_Q6 |  | 177 | 0.23 | 1.02 | 0.436 | 0.652 | 1.000 | 3747 | tags=33%, list=28%, signal=45% |
| 1206 | CAGGTA\_V$AREB6\_01 |  | 518 | 0.21 | 1.01 | 0.428 | 0.653 | 1.000 | 3621 | tags=30%, list=27%, signal=39% |
| 1207 | CHR1Q42 |  | 59 | 0.27 | 1.01 | 0.433 | 0.655 | 1.000 | 1808 | tags=20%, list=14%, signal=23% |
| 1208 | SYATTGTG\_UNKNOWN |  | 154 | 0.23 | 1.01 | 0.427 | 0.655 | 1.000 | 4132 | tags=36%, list=31%, signal=52% |
| 1209 | V$ATF1\_Q6 |  | 167 | 0.23 | 1.01 | 0.434 | 0.655 | 1.000 | 1866 | tags=17%, list=14%, signal=20% |
| 1210 | STTTCRNTTT\_V$IRF\_Q6 |  | 138 | 0.23 | 1.01 | 0.431 | 0.656 | 1.000 | 3148 | tags=27%, list=24%, signal=35% |
| 1211 | YNGTTNNNATT\_UNKNOWN |  | 240 | 0.22 | 1.01 | 0.430 | 0.656 | 1.000 | 3817 | tags=33%, list=29%, signal=45% |
| 1212 | GERY\_CEBP\_TARGETS |  | 106 | 0.24 | 1.01 | 0.419 | 0.656 | 1.000 | 604 | tags=10%, list=5%, signal=11% |
| 1213 | UVC\_HIGH\_D2\_DN |  | 37 | 0.30 | 1.01 | 0.430 | 0.658 | 1.000 | 2569 | tags=30%, list=19%, signal=37% |
| 1214 | AAAGGGA,MIR-204,MIR-211 |  | 169 | 0.23 | 1.01 | 0.439 | 0.658 | 1.000 | 1191 | tags=12%, list=9%, signal=13% |
| 1215 | SIG\_PIP3\_SIGNALING\_IN\_CARDIAC\_MYOCTES |  | 64 | 0.26 | 1.01 | 0.428 | 0.662 | 1.000 | 2688 | tags=28%, list=20%, signal=35% |
| 1216 | HDACI\_COLON\_SUL48HRS\_UP |  | 79 | 0.25 | 1.01 | 0.449 | 0.662 | 1.000 | 2391 | tags=27%, list=18%, signal=32% |
| 1217 | GPCRPATHWAY |  | 33 | 0.30 | 1.01 | 0.449 | 0.663 | 1.000 | 1775 | tags=18%, list=13%, signal=21% |
| 1218 | V$HNF1\_01 |  | 170 | 0.23 | 1.01 | 0.440 | 0.665 | 1.000 | 2746 | tags=21%, list=21%, signal=26% |
| 1219 | GAY\_YY1\_UP |  | 173 | 0.23 | 1.01 | 0.445 | 0.665 | 1.000 | 2217 | tags=20%, list=17%, signal=23% |
| 1220 | CHEMICALPATHWAY |  | 21 | 0.33 | 1.01 | 0.434 | 0.670 | 1.000 | 2915 | tags=29%, list=22%, signal=37% |
| 1221 | V$EGR2\_01 |  | 141 | 0.23 | 1.01 | 0.455 | 0.671 | 1.000 | 3800 | tags=32%, list=29%, signal=44% |
| 1222 | TATCTGG,MIR-488 |  | 46 | 0.28 | 1.00 | 0.443 | 0.673 | 1.000 | 3250 | tags=33%, list=24%, signal=43% |
| 1223 | V$SRY\_02 |  | 176 | 0.23 | 1.00 | 0.463 | 0.673 | 1.000 | 3717 | tags=30%, list=28%, signal=41% |
| 1224 | HADDAD\_HPCLYMPHO\_ENRICHED |  | 309 | 0.21 | 1.00 | 0.481 | 0.677 | 1.000 | 3170 | tags=27%, list=24%, signal=34% |
| 1225 | V$FXR\_Q3 |  | 78 | 0.25 | 1.00 | 0.464 | 0.677 | 1.000 | 3911 | tags=33%, list=29%, signal=47% |
| 1226 | IDX\_TSA\_UP\_CLUSTER5 |  | 88 | 0.25 | 1.00 | 0.458 | 0.676 | 1.000 | 2735 | tags=25%, list=21%, signal=31% |
| 1227 | V$PEA3\_Q6 |  | 173 | 0.23 | 1.00 | 0.478 | 0.676 | 1.000 | 4307 | tags=43%, list=32%, signal=63% |
| 1228 | GGAMTNNNNNTCCY\_UNKNOWN |  | 79 | 0.25 | 1.00 | 0.449 | 0.676 | 1.000 | 1746 | tags=19%, list=13%, signal=22% |
| 1229 | OXSTRESS\_BREASTCA\_UP |  | 25 | 0.32 | 1.00 | 0.434 | 0.676 | 1.000 | 922 | tags=20%, list=7%, signal=21% |
| 1230 | ECMPATHWAY |  | 21 | 0.33 | 1.00 | 0.453 | 0.677 | 1.000 | 3460 | tags=38%, list=26%, signal=51% |
| 1231 | ZHAN\_TONSIL\_BONEMARROW |  | 41 | 0.29 | 1.00 | 0.452 | 0.679 | 1.000 | 2594 | tags=34%, list=19%, signal=42% |
| 1232 | TATAAA\_V$TATA\_01 |  | 897 | 0.20 | 1.00 | 0.479 | 0.679 | 1.000 | 3227 | tags=24%, list=24%, signal=30% |
| 1233 | V$MYOD\_01 |  | 169 | 0.23 | 1.00 | 0.447 | 0.680 | 1.000 | 2105 | tags=20%, list=16%, signal=23% |
| 1234 | AGEING\_KIDNEY\_SPECIFIC\_UP |  | 147 | 0.23 | 1.00 | 0.460 | 0.681 | 1.000 | 2671 | tags=27%, list=20%, signal=33% |
| 1235 | HASLINGER\_B\_CLL\_13Q14 |  | 19 | 0.34 | 1.00 | 0.453 | 0.684 | 1.000 | 1506 | tags=21%, list=11%, signal=24% |
| 1236 | V$CREB\_01 |  | 180 | 0.22 | 1.00 | 0.483 | 0.684 | 1.000 | 3486 | tags=28%, list=26%, signal=38% |
| 1237 | 5FU\_RESIST\_GASTRIC\_DN |  | 16 | 0.36 | 1.00 | 0.477 | 0.684 | 1.000 | 2781 | tags=44%, list=21%, signal=55% |
| 1238 | CCCNNGGGAR\_V$OLF1\_01 |  | 221 | 0.22 | 1.00 | 0.464 | 0.684 | 1.000 | 3931 | tags=36%, list=30%, signal=50% |
| 1239 | ALZHEIMERS\_INCIPIENT\_UP |  | 338 | 0.21 | 1.00 | 0.456 | 0.684 | 1.000 | 2692 | tags=23%, list=20%, signal=28% |
| 1240 | V$MEIS1\_01 |  | 160 | 0.23 | 1.00 | 0.460 | 0.684 | 1.000 | 3926 | tags=36%, list=29%, signal=50% |
| 1241 | V$NKX25\_01 |  | 87 | 0.25 | 1.00 | 0.465 | 0.684 | 1.000 | 3306 | tags=29%, list=25%, signal=38% |
| 1242 | ACTWSNACTNY\_UNKNOWN |  | 54 | 0.27 | 1.00 | 0.467 | 0.684 | 1.000 | 331 | tags=11%, list=2%, signal=11% |
| 1243 | ET743\_SARCOMA\_UP |  | 65 | 0.26 | 1.00 | 0.478 | 0.684 | 1.000 | 2678 | tags=26%, list=20%, signal=33% |
| 1244 | WERNERONLY\_FIBRO\_DN |  | 53 | 0.27 | 1.00 | 0.456 | 0.684 | 1.000 | 3319 | tags=36%, list=25%, signal=48% |
| 1245 | HDACI\_COLON\_SUL\_UP |  | 113 | 0.24 | 1.00 | 0.447 | 0.684 | 1.000 | 2753 | tags=28%, list=21%, signal=35% |
| 1246 | HSC\_HSC\_FETAL |  | 190 | 0.22 | 1.00 | 0.471 | 0.684 | 1.000 | 2107 | tags=19%, list=16%, signal=23% |
| 1247 | HSC\_HSC\_SHARED |  | 179 | 0.22 | 1.00 | 0.465 | 0.684 | 1.000 | 2107 | tags=20%, list=16%, signal=23% |
| 1248 | CHR10Q21 |  | 29 | 0.30 | 1.00 | 0.482 | 0.684 | 1.000 | 2671 | tags=24%, list=20%, signal=30% |
| 1249 | TGFBETA\_LATE\_UP |  | 33 | 0.30 | 1.00 | 0.458 | 0.684 | 1.000 | 3098 | tags=33%, list=23%, signal=43% |
| 1250 | V$ARNT\_01 |  | 187 | 0.22 | 1.00 | 0.488 | 0.684 | 1.000 | 2727 | tags=24%, list=20%, signal=29% |
| 1251 | V$CEBPDELTA\_Q6 |  | 172 | 0.22 | 0.99 | 0.480 | 0.684 | 1.000 | 3533 | tags=30%, list=27%, signal=40% |
| 1252 | GGGACCA,MIR-133A,MIR-133B |  | 145 | 0.23 | 0.99 | 0.477 | 0.684 | 1.000 | 2967 | tags=29%, list=22%, signal=37% |
| 1253 | CAGCACT,MIR-512-3P |  | 106 | 0.24 | 0.99 | 0.468 | 0.686 | 1.000 | 1656 | tags=16%, list=12%, signal=18% |
| 1254 | AACATTC,MIR-409-3P |  | 113 | 0.24 | 0.99 | 0.487 | 0.686 | 1.000 | 2999 | tags=28%, list=23%, signal=36% |
| 1255 | HYPOXIA\_NORMAL\_UP |  | 196 | 0.22 | 0.99 | 0.476 | 0.687 | 1.000 | 2268 | tags=20%, list=17%, signal=24% |
| 1256 | CTTTAAR\_UNKNOWN |  | 662 | 0.20 | 0.99 | 0.499 | 0.688 | 1.000 | 2965 | tags=23%, list=22%, signal=28% |
| 1257 | OXSTRESS\_RPE\_HNETBH\_DN |  | 47 | 0.27 | 0.99 | 0.475 | 0.688 | 1.000 | 3747 | tags=38%, list=28%, signal=53% |
| 1258 | FLECHNER\_KIDNEY\_TRANSPLANT\_WELL\_UP |  | 554 | 0.20 | 0.99 | 0.498 | 0.689 | 1.000 | 2235 | tags=18%, list=17%, signal=21% |
| 1259 | V$PBX1\_02 |  | 95 | 0.24 | 0.99 | 0.480 | 0.690 | 1.000 | 4408 | tags=40%, list=33%, signal=59% |
| 1260 | TTCYNRGAA\_V$STAT5B\_01 |  | 225 | 0.22 | 0.99 | 0.474 | 0.689 | 1.000 | 3558 | tags=26%, list=27%, signal=35% |
| 1261 | V$STAT5A\_02 |  | 98 | 0.25 | 0.99 | 0.471 | 0.689 | 1.000 | 4035 | tags=36%, list=30%, signal=51% |
| 1262 | V$HNF3ALPHA\_Q6 |  | 155 | 0.23 | 0.99 | 0.465 | 0.690 | 1.000 | 4068 | tags=38%, list=31%, signal=54% |
| 1263 | V$MEF2\_02 |  | 164 | 0.22 | 0.99 | 0.493 | 0.690 | 1.000 | 4235 | tags=34%, list=32%, signal=49% |
| 1264 | CHR15Q13 |  | 15 | 0.36 | 0.99 | 0.485 | 0.690 | 1.000 | 3759 | tags=33%, list=28%, signal=46% |
| 1265 | V$CHOP\_01 |  | 177 | 0.22 | 0.99 | 0.486 | 0.691 | 1.000 | 3937 | tags=31%, list=30%, signal=43% |
| 1266 | HDACI\_COLON\_SUL2HRS\_DN |  | 15 | 0.36 | 0.99 | 0.468 | 0.692 | 1.000 | 709 | tags=13%, list=5%, signal=14% |
| 1267 | CITED1\_KO\_HET\_DN |  | 30 | 0.30 | 0.99 | 0.479 | 0.692 | 1.000 | 1396 | tags=20%, list=10%, signal=22% |
| 1268 | CAGGGTC,MIR-504 |  | 59 | 0.26 | 0.99 | 0.493 | 0.692 | 1.000 | 3573 | tags=34%, list=27%, signal=46% |
| 1269 | RIBAVIRIN\_RSV\_DN |  | 43 | 0.29 | 0.99 | 0.462 | 0.692 | 1.000 | 2329 | tags=26%, list=17%, signal=31% |
| 1270 | HUMAN\_TISSUE\_PLACENTA |  | 19 | 0.34 | 0.99 | 0.482 | 0.694 | 1.000 | 5446 | tags=53%, list=41%, signal=89% |
| 1271 | SA\_PTEN\_PATHWAY |  | 17 | 0.34 | 0.99 | 0.484 | 0.695 | 1.000 | 3460 | tags=41%, list=26%, signal=56% |
| 1272 | CTTTGTA,MIR-524 |  | 334 | 0.21 | 0.99 | 0.522 | 0.695 | 1.000 | 2879 | tags=22%, list=22%, signal=28% |
| 1273 | CHR7Q22 |  | 72 | 0.25 | 0.99 | 0.490 | 0.696 | 1.000 | 3848 | tags=35%, list=29%, signal=49% |
| 1274 | CHR17P13 |  | 145 | 0.23 | 0.99 | 0.505 | 0.696 | 1.000 | 5084 | tags=41%, list=38%, signal=66% |
| 1275 | TCELL\_ANERGIC\_UP |  | 76 | 0.25 | 0.99 | 0.474 | 0.696 | 1.000 | 2663 | tags=26%, list=20%, signal=33% |
| 1276 | V$AP2REP\_01 |  | 115 | 0.23 | 0.99 | 0.483 | 0.697 | 1.000 | 4271 | tags=38%, list=32%, signal=56% |
| 1277 | GCGNNANTTCC\_UNKNOWN |  | 84 | 0.25 | 0.98 | 0.485 | 0.697 | 1.000 | 2706 | tags=32%, list=20%, signal=40% |
| 1278 | CHR6Q25 |  | 32 | 0.30 | 0.98 | 0.471 | 0.697 | 1.000 | 2606 | tags=34%, list=20%, signal=43% |
| 1279 | HSP27PATHWAY |  | 15 | 0.37 | 0.98 | 0.491 | 0.697 | 1.000 | 922 | tags=20%, list=7%, signal=21% |
| 1280 | CTCAGGG,MIR-125B,MIR-125A |  | 235 | 0.21 | 0.98 | 0.511 | 0.697 | 1.000 | 2391 | tags=22%, list=18%, signal=26% |
| 1281 | ACCTGTTG\_UNKNOWN |  | 109 | 0.24 | 0.98 | 0.482 | 0.696 | 1.000 | 3065 | tags=28%, list=23%, signal=35% |
| 1282 | TCA |  | 15 | 0.36 | 0.98 | 0.505 | 0.697 | 1.000 | 3331 | tags=40%, list=25%, signal=53% |
| 1283 | V$SRF\_Q5\_01 |  | 170 | 0.22 | 0.98 | 0.506 | 0.701 | 1.000 | 2943 | tags=28%, list=22%, signal=35% |
| 1284 | TGACGTCA\_V$ATF3\_Q6 |  | 160 | 0.22 | 0.98 | 0.507 | 0.703 | 1.000 | 3561 | tags=29%, list=27%, signal=39% |
| 1285 | SIG\_INSULIN\_RECEPTOR\_PATHWAY\_IN\_CARDIAC\_MYOCYTES |  | 50 | 0.27 | 0.98 | 0.479 | 0.703 | 1.000 | 2517 | tags=26%, list=19%, signal=32% |
| 1286 | ERKPATHWAY |  | 30 | 0.30 | 0.98 | 0.475 | 0.703 | 1.000 | 3811 | tags=37%, list=29%, signal=51% |
| 1287 | V$CREB\_02 |  | 178 | 0.22 | 0.98 | 0.516 | 0.703 | 1.000 | 4336 | tags=38%, list=33%, signal=55% |
| 1288 | GTGCAAT,MIR-25,MIR-32,MIR-92,MIR-363,MIR-367 |  | 235 | 0.21 | 0.98 | 0.528 | 0.704 | 1.000 | 2690 | tags=24%, list=20%, signal=29% |
| 1289 | GTAAACC,MIR-299-5P |  | 38 | 0.29 | 0.98 | 0.476 | 0.704 | 1.000 | 2226 | tags=26%, list=17%, signal=32% |
| 1290 | CHR6Q27 |  | 23 | 0.33 | 0.98 | 0.481 | 0.703 | 1.000 | 2994 | tags=30%, list=22%, signal=39% |
| 1291 | BYSTRYKH\_HSC\_TRANS\_GLOCUS |  | 698 | 0.19 | 0.98 | 0.546 | 0.704 | 1.000 | 2759 | tags=22%, list=21%, signal=27% |
| 1292 | V$SPZ1\_01 |  | 172 | 0.22 | 0.98 | 0.517 | 0.705 | 1.000 | 1969 | tags=18%, list=15%, signal=21% |
| 1293 | CHR10Q24 |  | 78 | 0.25 | 0.98 | 0.496 | 0.708 | 1.000 | 1949 | tags=19%, list=15%, signal=22% |
| 1294 | YRCCAKNNGNCGC\_UNKNOWN |  | 50 | 0.27 | 0.98 | 0.503 | 0.709 | 1.000 | 3864 | tags=42%, list=29%, signal=59% |
| 1295 | TCCCRNNRTGC\_UNKNOWN |  | 130 | 0.23 | 0.98 | 0.503 | 0.709 | 1.000 | 3135 | tags=28%, list=24%, signal=37% |
| 1296 | GCACCTT,MIR-18A,MIR-18B |  | 86 | 0.24 | 0.98 | 0.507 | 0.709 | 1.000 | 1841 | tags=16%, list=14%, signal=19% |
| 1297 | TOLLPATHWAY |  | 30 | 0.30 | 0.98 | 0.516 | 0.710 | 1.000 | 2969 | tags=33%, list=22%, signal=43% |
| 1298 | V$HOXA4\_Q2 |  | 185 | 0.22 | 0.98 | 0.518 | 0.710 | 1.000 | 4224 | tags=36%, list=32%, signal=52% |
| 1299 | CAGCTG\_V$AP4\_Q5 |  | 1013 | 0.19 | 0.98 | 0.575 | 0.710 | 1.000 | 3441 | tags=27%, list=26%, signal=33% |
| 1300 | LIAN\_MYELOID\_DIFF\_GRANULE |  | 24 | 0.32 | 0.98 | 0.500 | 0.711 | 1.000 | 3453 | tags=42%, list=26%, signal=56% |
| 1301 | AGED\_RHESUS\_DN |  | 107 | 0.23 | 0.98 | 0.500 | 0.711 | 1.000 | 1642 | tags=16%, list=12%, signal=18% |
| 1302 | LEE\_CIP\_DN |  | 65 | 0.26 | 0.97 | 0.484 | 0.713 | 1.000 | 2099 | tags=23%, list=16%, signal=27% |
| 1303 | CHR15Q14 |  | 24 | 0.31 | 0.97 | 0.486 | 0.713 | 1.000 | 4084 | tags=38%, list=31%, signal=54% |
| 1304 | PAR1PATHWAY |  | 19 | 0.33 | 0.97 | 0.502 | 0.713 | 1.000 | 3608 | tags=37%, list=27%, signal=50% |
| 1305 | UVC\_LOW\_ALL\_DN |  | 58 | 0.26 | 0.97 | 0.496 | 0.714 | 1.000 | 3428 | tags=36%, list=26%, signal=49% |
| 1306 | HSC\_INTERMEDIATEPROGENITORS\_ADULT |  | 114 | 0.23 | 0.97 | 0.515 | 0.714 | 1.000 | 1828 | tags=20%, list=14%, signal=23% |
| 1307 | TTTGTAG,MIR-520D |  | 253 | 0.21 | 0.97 | 0.538 | 0.714 | 1.000 | 3886 | tags=32%, list=29%, signal=45% |
| 1308 | V$IRF2\_01 |  | 89 | 0.24 | 0.97 | 0.500 | 0.715 | 1.000 | 3605 | tags=34%, list=27%, signal=46% |
| 1309 | CHR13Q34 |  | 29 | 0.30 | 0.97 | 0.499 | 0.714 | 1.000 | 2396 | tags=24%, list=18%, signal=29% |
| 1310 | HIVNEFPATHWAY |  | 55 | 0.26 | 0.97 | 0.516 | 0.716 | 1.000 | 1726 | tags=20%, list=13%, signal=23% |
| 1311 | V$CEBP\_Q3 |  | 171 | 0.22 | 0.97 | 0.532 | 0.716 | 1.000 | 3953 | tags=30%, list=30%, signal=43% |
| 1312 | CMV\_HCMV\_TIMECOURSE\_10HRS\_DN |  | 15 | 0.36 | 0.97 | 0.498 | 0.716 | 1.000 | 1778 | tags=40%, list=13%, signal=46% |
| 1313 | CTTGTAT,MIR-381 |  | 161 | 0.22 | 0.97 | 0.538 | 0.716 | 1.000 | 2693 | tags=22%, list=20%, signal=28% |
| 1314 | V$FOXO1\_02 |  | 162 | 0.22 | 0.97 | 0.527 | 0.717 | 1.000 | 3817 | tags=32%, list=29%, signal=44% |
| 1315 | GGTGAAG,MIR-412 |  | 46 | 0.27 | 0.97 | 0.494 | 0.717 | 1.000 | 2585 | tags=24%, list=19%, signal=30% |
| 1316 | CMV\_HCMV\_TIMECOURSE\_ALL\_DN |  | 420 | 0.20 | 0.97 | 0.576 | 0.717 | 1.000 | 2359 | tags=20%, list=18%, signal=24% |
| 1317 | XPB\_TTD-CS\_UP |  | 26 | 0.31 | 0.97 | 0.510 | 0.716 | 1.000 | 2860 | tags=35%, list=21%, signal=44% |
| 1318 | V$MYOD\_Q6 |  | 166 | 0.22 | 0.97 | 0.508 | 0.719 | 1.000 | 2975 | tags=26%, list=22%, signal=33% |
| 1319 | V$NMYC\_01 |  | 186 | 0.22 | 0.97 | 0.524 | 0.719 | 1.000 | 4339 | tags=38%, list=33%, signal=55% |
| 1320 | ATAGGAA,MIR-202 |  | 83 | 0.24 | 0.97 | 0.522 | 0.721 | 1.000 | 4453 | tags=39%, list=33%, signal=58% |
| 1321 | IL3PATHWAY |  | 15 | 0.35 | 0.97 | 0.506 | 0.720 | 1.000 | 1775 | tags=27%, list=13%, signal=31% |
| 1322 | GPCRS\_CLASS\_A\_RHODOPSIN\_LIKE |  | 130 | 0.23 | 0.97 | 0.530 | 0.720 | 1.000 | 4660 | tags=40%, list=35%, signal=61% |
| 1323 | IRS\_KO\_ADIP\_DN |  | 37 | 0.28 | 0.96 | 0.526 | 0.727 | 1.000 | 2426 | tags=30%, list=18%, signal=36% |
| 1324 | HOUSTIS\_ROS |  | 30 | 0.29 | 0.96 | 0.528 | 0.727 | 1.000 | 3256 | tags=33%, list=24%, signal=44% |
| 1325 | CHIARETTI\_ZAP70\_DIFF |  | 67 | 0.25 | 0.96 | 0.517 | 0.730 | 1.000 | 3229 | tags=33%, list=24%, signal=43% |
| 1326 | CHR5P15 |  | 39 | 0.28 | 0.96 | 0.515 | 0.731 | 1.000 | 1924 | tags=21%, list=14%, signal=24% |
| 1327 | GGGYGTGNY\_UNKNOWN |  | 454 | 0.20 | 0.96 | 0.592 | 0.732 | 1.000 | 3175 | tags=26%, list=24%, signal=33% |
| 1328 | V$CDC5\_01 |  | 189 | 0.21 | 0.96 | 0.549 | 0.732 | 1.000 | 3148 | tags=29%, list=24%, signal=37% |
| 1329 | CHR12Q21 |  | 28 | 0.30 | 0.96 | 0.494 | 0.733 | 1.000 | 1961 | tags=21%, list=15%, signal=25% |
| 1330 | BCNU\_GLIOMA\_NOMGMT\_48HRS\_UP |  | 18 | 0.34 | 0.96 | 0.539 | 0.734 | 1.000 | 2083 | tags=28%, list=16%, signal=33% |
| 1331 | CHR4P14 |  | 16 | 0.34 | 0.96 | 0.497 | 0.733 | 1.000 | 1056 | tags=19%, list=8%, signal=20% |
| 1332 | UVB\_NHEK3\_C3 |  | 17 | 0.35 | 0.96 | 0.503 | 0.733 | 1.000 | 3545 | tags=41%, list=27%, signal=56% |
| 1333 | V$PXR\_Q2 |  | 187 | 0.21 | 0.96 | 0.554 | 0.735 | 1.000 | 3864 | tags=33%, list=29%, signal=45% |
| 1334 | TTANWNANTGGM\_UNKNOWN |  | 40 | 0.28 | 0.96 | 0.539 | 0.735 | 1.000 | 2724 | tags=33%, list=20%, signal=41% |
| 1335 | CACTTTG,MIR-520G,MIR-520H |  | 176 | 0.22 | 0.96 | 0.570 | 0.735 | 1.000 | 2686 | tags=22%, list=20%, signal=27% |
| 1336 | YATTNATC\_UNKNOWN |  | 255 | 0.21 | 0.96 | 0.571 | 0.735 | 1.000 | 4013 | tags=32%, list=30%, signal=45% |
| 1337 | CHR1Q31 |  | 30 | 0.30 | 0.96 | 0.531 | 0.740 | 1.000 | 5602 | tags=53%, list=42%, signal=92% |
| 1338 | GLYCINE\_SERINE\_AND\_THREONINE\_METABOLISM |  | 32 | 0.29 | 0.96 | 0.509 | 0.740 | 1.000 | 591 | tags=13%, list=4%, signal=13% |
| 1339 | CALCIUM\_REGULATION\_IN\_CARDIAC\_CELLS |  | 127 | 0.22 | 0.96 | 0.565 | 0.743 | 1.000 | 5146 | tags=46%, list=39%, signal=74% |
| 1340 | KNUDSEN\_PMNS\_DN |  | 225 | 0.21 | 0.96 | 0.577 | 0.743 | 1.000 | 3896 | tags=34%, list=29%, signal=47% |
| 1341 | V$GR\_Q6\_01 |  | 183 | 0.22 | 0.96 | 0.566 | 0.742 | 1.000 | 3547 | tags=30%, list=27%, signal=40% |
| 1342 | INOSITOL\_PHOSPHATE\_METABOLISM |  | 24 | 0.30 | 0.96 | 0.495 | 0.742 | 1.000 | 2327 | tags=25%, list=17%, signal=30% |
| 1343 | AGED\_MOUSE\_CEREBELLUM\_DN |  | 45 | 0.27 | 0.96 | 0.514 | 0.742 | 1.000 | 6051 | tags=58%, list=45%, signal=106% |
| 1344 | ZHAN\_PCS\_MULTIPLE\_MYELOMA\_SPKD |  | 23 | 0.32 | 0.96 | 0.561 | 0.742 | 1.000 | 1286 | tags=22%, list=10%, signal=24% |
| 1345 | V$PU1\_Q6 |  | 162 | 0.22 | 0.96 | 0.581 | 0.742 | 1.000 | 2826 | tags=24%, list=21%, signal=30% |
| 1346 | KCCGNSWTTT\_UNKNOWN |  | 79 | 0.24 | 0.95 | 0.551 | 0.742 | 1.000 | 1805 | tags=16%, list=14%, signal=19% |
| 1347 | HSC\_INTERMEDIATEPROGENITORS\_FETAL |  | 124 | 0.22 | 0.95 | 0.565 | 0.742 | 1.000 | 1828 | tags=20%, list=14%, signal=23% |
| 1348 | AAGCACT,MIR-520F |  | 168 | 0.21 | 0.95 | 0.567 | 0.741 | 1.000 | 1875 | tags=18%, list=14%, signal=21% |
| 1349 | WYAAANNRNNNGCG\_UNKNOWN |  | 43 | 0.27 | 0.95 | 0.551 | 0.745 | 1.000 | 1301 | tags=16%, list=10%, signal=18% |
| 1350 | UV\_UNIQUE\_FIBRO\_UP |  | 20 | 0.33 | 0.95 | 0.537 | 0.746 | 1.000 | 2961 | tags=30%, list=22%, signal=39% |
| 1351 | V$ATF\_B |  | 136 | 0.22 | 0.95 | 0.556 | 0.747 | 1.000 | 1751 | tags=17%, list=13%, signal=19% |
| 1352 | GATGKMRGCG\_UNKNOWN |  | 45 | 0.27 | 0.95 | 0.537 | 0.748 | 1.000 | 1383 | tags=13%, list=10%, signal=15% |
| 1353 | CITED1\_KO\_HET\_UP |  | 24 | 0.31 | 0.95 | 0.520 | 0.750 | 1.000 | 3053 | tags=42%, list=23%, signal=54% |
| 1354 | ACACTAC,MIR-142-3P |  | 102 | 0.23 | 0.95 | 0.548 | 0.750 | 1.000 | 2724 | tags=25%, list=20%, signal=31% |
| 1355 | TTGCCAA,MIR-182 |  | 248 | 0.21 | 0.95 | 0.594 | 0.751 | 1.000 | 3743 | tags=32%, list=28%, signal=44% |
| 1356 | TCCATTKW\_UNKNOWN |  | 165 | 0.21 | 0.95 | 0.574 | 0.751 | 1.000 | 3150 | tags=27%, list=24%, signal=34% |
| 1357 | CHR8P22 |  | 30 | 0.29 | 0.95 | 0.549 | 0.751 | 1.000 | 4408 | tags=43%, list=33%, signal=65% |
| 1358 | RGAANNTTC\_V$HSF1\_01 |  | 304 | 0.20 | 0.95 | 0.606 | 0.751 | 1.000 | 3093 | tags=27%, list=23%, signal=35% |
| 1359 | V$MEF2\_01 |  | 108 | 0.23 | 0.95 | 0.568 | 0.751 | 1.000 | 3395 | tags=30%, list=25%, signal=39% |
| 1360 | HOGERKORP\_ANTI\_CD44\_UP |  | 23 | 0.31 | 0.95 | 0.527 | 0.751 | 1.000 | 1546 | tags=17%, list=12%, signal=20% |
| 1361 | V$ZF5\_B |  | 168 | 0.22 | 0.95 | 0.599 | 0.751 | 1.000 | 3998 | tags=35%, list=30%, signal=49% |
| 1362 | TNFR1PATHWAY |  | 28 | 0.29 | 0.95 | 0.537 | 0.750 | 1.000 | 3098 | tags=36%, list=23%, signal=46% |
| 1363 | PENTOSE\_PHOSPHATE\_PATHWAY |  | 23 | 0.31 | 0.95 | 0.543 | 0.751 | 1.000 | 1698 | tags=26%, list=13%, signal=30% |
| 1364 | V$CACCCBINDINGFACTOR\_Q6 |  | 188 | 0.21 | 0.95 | 0.602 | 0.753 | 1.000 | 3926 | tags=31%, list=29%, signal=44% |
| 1365 | HDACI\_COLON\_SUL16HRS\_DN |  | 64 | 0.25 | 0.95 | 0.548 | 0.753 | 1.000 | 3806 | tags=36%, list=29%, signal=50% |
| 1366 | ACTAYRNNNCCCR\_UNKNOWN |  | 267 | 0.20 | 0.95 | 0.613 | 0.753 | 1.000 | 3942 | tags=32%, list=30%, signal=45% |
| 1367 | HSC\_INTERMEDIATEPROGENITORS\_SHARED |  | 105 | 0.23 | 0.95 | 0.581 | 0.752 | 1.000 | 2787 | tags=28%, list=21%, signal=35% |
| 1368 | ZHAN\_MM\_CD1\_VS\_CD2\_DN |  | 42 | 0.27 | 0.95 | 0.556 | 0.752 | 1.000 | 2761 | tags=33%, list=21%, signal=42% |
| 1369 | CHESLER\_HIGHEST\_FOLD\_RANGE\_GENES |  | 33 | 0.28 | 0.95 | 0.535 | 0.752 | 1.000 | 679 | tags=12%, list=5%, signal=13% |
| 1370 | MTORPATHWAY |  | 23 | 0.31 | 0.95 | 0.543 | 0.754 | 1.000 | 1557 | tags=17%, list=12%, signal=20% |
| 1371 | LU\_IL4BCELL |  | 62 | 0.25 | 0.95 | 0.583 | 0.754 | 1.000 | 2276 | tags=27%, list=17%, signal=33% |
| 1372 | BRUNO\_IL3\_DN |  | 63 | 0.24 | 0.94 | 0.562 | 0.757 | 1.000 | 3019 | tags=32%, list=23%, signal=41% |
| 1373 | MITOCHONDRIA |  | 387 | 0.20 | 0.94 | 0.636 | 0.758 | 1.000 | 2476 | tags=21%, list=19%, signal=25% |
| 1374 | YTAATTAA\_V$LHX3\_01 |  | 128 | 0.22 | 0.94 | 0.595 | 0.759 | 1.000 | 2814 | tags=23%, list=21%, signal=29% |
| 1375 | V$NKX3A\_01 |  | 147 | 0.22 | 0.94 | 0.595 | 0.761 | 1.000 | 2224 | tags=18%, list=17%, signal=22% |
| 1376 | V$OCT1\_03 |  | 156 | 0.22 | 0.94 | 0.598 | 0.761 | 1.000 | 4465 | tags=38%, list=34%, signal=56% |
| 1377 | V$PPAR\_DR1\_Q2 |  | 181 | 0.21 | 0.94 | 0.608 | 0.761 | 1.000 | 4486 | tags=35%, list=34%, signal=53% |
| 1378 | ACTTTAT,MIR-142-5P |  | 211 | 0.21 | 0.94 | 0.609 | 0.762 | 1.000 | 2548 | tags=22%, list=19%, signal=27% |
| 1379 | LEE\_MYC\_E2F1\_DN |  | 61 | 0.25 | 0.94 | 0.556 | 0.764 | 1.000 | 1090 | tags=13%, list=8%, signal=14% |
| 1380 | CHR4Q12 |  | 23 | 0.31 | 0.94 | 0.552 | 0.764 | 1.000 | 3101 | tags=30%, list=23%, signal=40% |
| 1381 | GLYCEROLIPID\_METABOLISM |  | 40 | 0.27 | 0.94 | 0.577 | 0.765 | 1.000 | 4400 | tags=38%, list=33%, signal=56% |
| 1382 | GATAAGR\_V$GATA\_C |  | 216 | 0.20 | 0.94 | 0.609 | 0.765 | 1.000 | 4038 | tags=31%, list=30%, signal=44% |
| 1383 | ALANINE\_AND\_ASPARTATE\_METABOLISM |  | 19 | 0.32 | 0.94 | 0.535 | 0.765 | 1.000 | 1154 | tags=21%, list=9%, signal=23% |
| 1384 | V$AP4\_Q5 |  | 181 | 0.21 | 0.94 | 0.608 | 0.764 | 1.000 | 3096 | tags=24%, list=23%, signal=31% |
| 1385 | LEE\_CIP\_UP |  | 60 | 0.25 | 0.94 | 0.587 | 0.764 | 1.000 | 1454 | tags=20%, list=11%, signal=22% |
| 1386 | V$AP4\_Q6\_01 |  | 183 | 0.21 | 0.94 | 0.590 | 0.764 | 1.000 | 3459 | tags=31%, list=26%, signal=41% |
| 1387 | CTGYNNCTYTAA\_UNKNOWN |  | 51 | 0.26 | 0.94 | 0.563 | 0.765 | 1.000 | 3197 | tags=33%, list=24%, signal=44% |
| 1388 | HDACI\_COLON\_CUR2HRS\_UP |  | 26 | 0.30 | 0.94 | 0.553 | 0.764 | 1.000 | 1262 | tags=23%, list=9%, signal=25% |
| 1389 | BENNETT\_SLE\_UP |  | 28 | 0.29 | 0.94 | 0.555 | 0.764 | 1.000 | 4842 | tags=46%, list=36%, signal=73% |
| 1390 | CTAGGAA,MIR-384 |  | 49 | 0.26 | 0.94 | 0.572 | 0.765 | 1.000 | 2226 | tags=22%, list=17%, signal=27% |
| 1391 | YKACATTT\_UNKNOWN |  | 186 | 0.21 | 0.94 | 0.608 | 0.764 | 1.000 | 4166 | tags=33%, list=31%, signal=48% |
| 1392 | WERNER\_FIBRO\_UP |  | 48 | 0.26 | 0.93 | 0.565 | 0.769 | 1.000 | 2463 | tags=25%, list=18%, signal=31% |
| 1393 | V$FOX\_Q2 |  | 152 | 0.21 | 0.93 | 0.616 | 0.770 | 1.000 | 3953 | tags=31%, list=30%, signal=43% |
| 1394 | SHEPARD\_CRASH\_AND\_BURN\_MUT\_VS\_WT\_UP |  | 137 | 0.22 | 0.93 | 0.617 | 0.772 | 1.000 | 2990 | tags=26%, list=22%, signal=34% |
| 1395 | V$FREAC3\_01 |  | 167 | 0.21 | 0.93 | 0.610 | 0.771 | 1.000 | 3782 | tags=31%, list=28%, signal=43% |
| 1396 | FLECHNER\_KIDNEY\_TRANSPLANT\_REJECTION\_PBL\_UP |  | 64 | 0.24 | 0.93 | 0.600 | 0.771 | 1.000 | 2709 | tags=27%, list=20%, signal=33% |
| 1397 | V$WHN\_B |  | 168 | 0.21 | 0.93 | 0.606 | 0.771 | 1.000 | 1917 | tags=20%, list=14%, signal=23% |
| 1398 | ELECTRON\_TRANSPORT\_CHAIN |  | 90 | 0.23 | 0.93 | 0.596 | 0.775 | 1.000 | 1768 | tags=19%, list=13%, signal=22% |
| 1399 | WERNERONLY\_FIBRO\_UP |  | 27 | 0.29 | 0.93 | 0.557 | 0.776 | 1.000 | 4486 | tags=48%, list=34%, signal=72% |
| 1400 | UVC\_HIGH\_D7\_DN |  | 32 | 0.28 | 0.93 | 0.547 | 0.776 | 1.000 | 1105 | tags=19%, list=8%, signal=20% |
| 1401 | V$USF\_Q6\_01 |  | 172 | 0.21 | 0.93 | 0.637 | 0.776 | 1.000 | 2812 | tags=26%, list=21%, signal=32% |
| 1402 | IL2RBPATHWAY |  | 34 | 0.28 | 0.93 | 0.580 | 0.775 | 1.000 | 2009 | tags=26%, list=15%, signal=31% |
| 1403 | STEMCELL\_HEMATOPOIETIC\_UP |  | 1213 | 0.18 | 0.93 | 0.753 | 0.775 | 1.000 | 2808 | tags=22%, list=21%, signal=26% |
| 1404 | ESR\_FIBROBLAST\_DN |  | 15 | 0.33 | 0.93 | 0.552 | 0.775 | 1.000 | 2768 | tags=27%, list=21%, signal=34% |
| 1405 | CHR15Q11 |  | 26 | 0.29 | 0.93 | 0.546 | 0.774 | 1.000 | 1168 | tags=15%, list=9%, signal=17% |
| 1406 | HEARTFAILURE\_VENTRICLE\_DN |  | 66 | 0.24 | 0.93 | 0.575 | 0.776 | 1.000 | 2969 | tags=30%, list=22%, signal=39% |
| 1407 | CROONQUIST\_IL6\_RAS\_UP |  | 21 | 0.31 | 0.93 | 0.577 | 0.777 | 1.000 | 5009 | tags=67%, list=38%, signal=107% |
| 1408 | V$HNF4\_Q6 |  | 181 | 0.21 | 0.93 | 0.651 | 0.778 | 1.000 | 2277 | tags=21%, list=17%, signal=25% |
| 1409 | GN\_CAMP\_GRANULOSA\_DN |  | 61 | 0.24 | 0.93 | 0.587 | 0.779 | 1.000 | 1536 | tags=18%, list=12%, signal=20% |
| 1410 | CAGGTCC,MIR-492 |  | 40 | 0.27 | 0.93 | 0.595 | 0.779 | 1.000 | 3050 | tags=30%, list=23%, signal=39% |
| 1411 | CMV\_UV-CMV\_COMMON\_HCMV\_6HRS\_DN |  | 27 | 0.29 | 0.93 | 0.548 | 0.779 | 1.000 | 3490 | tags=41%, list=26%, signal=55% |
| 1412 | V$RORA2\_01 |  | 109 | 0.22 | 0.93 | 0.620 | 0.779 | 1.000 | 4358 | tags=38%, list=33%, signal=55% |
| 1413 | CHR2Q24 |  | 27 | 0.29 | 0.93 | 0.584 | 0.779 | 1.000 | 2530 | tags=26%, list=19%, signal=32% |
| 1414 | CCTGTGA,MIR-513 |  | 99 | 0.22 | 0.93 | 0.614 | 0.779 | 1.000 | 4936 | tags=46%, list=37%, signal=73% |
| 1415 | AAAGACA,MIR-511 |  | 154 | 0.21 | 0.92 | 0.631 | 0.782 | 1.000 | 2120 | tags=17%, list=16%, signal=20% |
| 1416 | VENTRICLES\_UP |  | 205 | 0.20 | 0.92 | 0.667 | 0.783 | 1.000 | 3894 | tags=37%, list=29%, signal=51% |
| 1417 | V$IK3\_01 |  | 146 | 0.21 | 0.92 | 0.632 | 0.784 | 1.000 | 3358 | tags=27%, list=25%, signal=36% |
| 1418 | ROSS\_CBF\_LEUKEMIA |  | 65 | 0.24 | 0.92 | 0.598 | 0.784 | 1.000 | 3083 | tags=35%, list=23%, signal=46% |
| 1419 | V$POU6F1\_01 |  | 163 | 0.21 | 0.92 | 0.639 | 0.785 | 1.000 | 3926 | tags=33%, list=29%, signal=46% |
| 1420 | CHR1Q22 |  | 48 | 0.25 | 0.92 | 0.586 | 0.785 | 1.000 | 1284 | tags=15%, list=10%, signal=16% |
| 1421 | V$LHX3\_01 |  | 152 | 0.21 | 0.92 | 0.634 | 0.785 | 1.000 | 2814 | tags=24%, list=21%, signal=30% |
| 1422 | XPB\_TTD-CS\_DN |  | 24 | 0.30 | 0.92 | 0.572 | 0.785 | 1.000 | 1746 | tags=17%, list=13%, signal=19% |
| 1423 | CHR8Q13 |  | 28 | 0.29 | 0.92 | 0.596 | 0.785 | 1.000 | 5671 | tags=54%, list=43%, signal=93% |
| 1424 | LH\_GRANULOSA\_DN |  | 76 | 0.24 | 0.92 | 0.602 | 0.785 | 1.000 | 4004 | tags=38%, list=30%, signal=54% |
| 1425 | FSH\_GRANULOSA\_DN |  | 76 | 0.24 | 0.92 | 0.601 | 0.787 | 1.000 | 4004 | tags=38%, list=30%, signal=54% |
| 1426 | V$COUP\_01 |  | 175 | 0.21 | 0.92 | 0.640 | 0.788 | 1.000 | 3561 | tags=27%, list=27%, signal=37% |
| 1427 | CHR6Q14 |  | 25 | 0.29 | 0.92 | 0.591 | 0.788 | 1.000 | 5338 | tags=60%, list=40%, signal=100% |
| 1428 | TUMOR\_SUPRESSOR |  | 22 | 0.31 | 0.92 | 0.575 | 0.788 | 1.000 | 1126 | tags=14%, list=8%, signal=15% |
| 1429 | WELCSH\_BRCA\_UP |  | 38 | 0.27 | 0.92 | 0.587 | 0.788 | 1.000 | 2368 | tags=26%, list=18%, signal=32% |
| 1430 | KERATINOCYTEPATHWAY |  | 43 | 0.26 | 0.92 | 0.582 | 0.788 | 1.000 | 1775 | tags=19%, list=13%, signal=21% |
| 1431 | CALCINEURIN\_NF\_AT\_SIGNALING |  | 91 | 0.23 | 0.92 | 0.625 | 0.788 | 1.000 | 4687 | tags=38%, list=35%, signal=59% |
| 1432 | ATTACAT,MIR-380-3P |  | 74 | 0.23 | 0.92 | 0.615 | 0.788 | 1.000 | 2965 | tags=26%, list=22%, signal=33% |
| 1433 | V$FOXO1\_01 |  | 172 | 0.21 | 0.92 | 0.645 | 0.787 | 1.000 | 3956 | tags=38%, list=30%, signal=53% |
| 1434 | CHR7Q21 |  | 64 | 0.24 | 0.92 | 0.611 | 0.787 | 1.000 | 2142 | tags=17%, list=16%, signal=20% |
| 1435 | BCRPATHWAY |  | 34 | 0.27 | 0.92 | 0.576 | 0.787 | 1.000 | 1859 | tags=21%, list=14%, signal=24% |
| 1436 | V$SREBP1\_02 |  | 60 | 0.24 | 0.92 | 0.596 | 0.789 | 1.000 | 5479 | tags=58%, list=41%, signal=99% |
| 1437 | RNCTGNYNRNCTGNY\_UNKNOWN |  | 45 | 0.26 | 0.92 | 0.621 | 0.790 | 1.000 | 4079 | tags=51%, list=31%, signal=73% |
| 1438 | YAATNANRNNNCAG\_UNKNOWN |  | 47 | 0.25 | 0.92 | 0.624 | 0.790 | 1.000 | 4538 | tags=40%, list=34%, signal=61% |
| 1439 | BRCA1KO\_MEF\_DN |  | 77 | 0.23 | 0.92 | 0.623 | 0.790 | 1.000 | 4273 | tags=39%, list=32%, signal=57% |
| 1440 | TTCYRGAA\_UNKNOWN |  | 219 | 0.20 | 0.92 | 0.655 | 0.791 | 1.000 | 4649 | tags=38%, list=35%, signal=57% |
| 1441 | V$STAT1\_02 |  | 172 | 0.21 | 0.92 | 0.669 | 0.791 | 1.000 | 1441 | tags=13%, list=11%, signal=15% |
| 1442 | GH\_EXOGENOUS\_ANY\_UP |  | 232 | 0.20 | 0.92 | 0.672 | 0.792 | 1.000 | 3765 | tags=29%, list=28%, signal=40% |
| 1443 | V$CREB\_Q4 |  | 175 | 0.20 | 0.91 | 0.670 | 0.792 | 1.000 | 3564 | tags=31%, list=27%, signal=42% |
| 1444 | RACCACAR\_V$AML\_Q6 |  | 169 | 0.21 | 0.91 | 0.665 | 0.792 | 1.000 | 4664 | tags=38%, list=35%, signal=58% |
| 1445 | V$OCT1\_04 |  | 167 | 0.21 | 0.91 | 0.676 | 0.791 | 1.000 | 3591 | tags=32%, list=27%, signal=44% |
| 1446 | ZMPSTE24\_KO\_UP |  | 30 | 0.28 | 0.91 | 0.586 | 0.792 | 1.000 | 2295 | tags=23%, list=17%, signal=28% |
| 1447 | H2O2\_CSBDIFF\_C1 |  | 32 | 0.28 | 0.91 | 0.580 | 0.791 | 1.000 | 2735 | tags=28%, list=21%, signal=35% |
| 1448 | SNACANNNYSYAGA\_UNKNOWN |  | 46 | 0.26 | 0.91 | 0.609 | 0.791 | 1.000 | 4436 | tags=41%, list=33%, signal=62% |
| 1449 | V$ETS1\_B |  | 177 | 0.21 | 0.91 | 0.677 | 0.792 | 1.000 | 3229 | tags=28%, list=24%, signal=37% |
| 1450 | HALMOS\_CEBP\_DN |  | 43 | 0.26 | 0.91 | 0.596 | 0.791 | 1.000 | 1286 | tags=16%, list=10%, signal=18% |
| 1451 | POD1\_KO\_MOST\_UP |  | 30 | 0.28 | 0.91 | 0.592 | 0.793 | 1.000 | 2777 | tags=30%, list=21%, signal=38% |
| 1452 | GCTTGAA,MIR-498 |  | 85 | 0.22 | 0.91 | 0.620 | 0.793 | 1.000 | 3579 | tags=29%, list=27%, signal=40% |
| 1453 | HSC\_HSC\_ADULT |  | 240 | 0.20 | 0.91 | 0.692 | 0.793 | 1.000 | 2107 | tags=18%, list=16%, signal=20% |
| 1454 | V$LMO2COM\_02 |  | 174 | 0.20 | 0.91 | 0.676 | 0.793 | 1.000 | 3632 | tags=27%, list=27%, signal=37% |
| 1455 | TGTATGA,MIR-485-3P |  | 108 | 0.22 | 0.91 | 0.641 | 0.792 | 1.000 | 4100 | tags=31%, list=31%, signal=45% |
| 1456 | SANSOM\_APC\_4\_DN |  | 52 | 0.25 | 0.91 | 0.615 | 0.792 | 1.000 | 2153 | tags=21%, list=16%, signal=25% |
| 1457 | CHR8P12 |  | 21 | 0.31 | 0.91 | 0.606 | 0.791 | 1.000 | 1436 | tags=24%, list=11%, signal=27% |
| 1458 | V$HFH1\_01 |  | 173 | 0.20 | 0.91 | 0.675 | 0.791 | 1.000 | 3406 | tags=28%, list=26%, signal=37% |
| 1459 | RESISTANCE\_XENOGRAFTS\_UP |  | 27 | 0.29 | 0.91 | 0.595 | 0.791 | 1.000 | 3133 | tags=33%, list=24%, signal=43% |
| 1460 | MGGAAGTG\_V$GABP\_B |  | 503 | 0.19 | 0.91 | 0.769 | 0.791 | 1.000 | 3121 | tags=25%, list=23%, signal=32% |
| 1461 | CCTNTMAGA\_UNKNOWN |  | 88 | 0.23 | 0.91 | 0.640 | 0.790 | 1.000 | 3263 | tags=32%, list=24%, signal=42% |
| 1462 | V$NERF\_Q2 |  | 173 | 0.21 | 0.91 | 0.671 | 0.790 | 1.000 | 3224 | tags=28%, list=24%, signal=36% |
| 1463 | V$MTF1\_Q4 |  | 178 | 0.21 | 0.91 | 0.660 | 0.792 | 1.000 | 3617 | tags=30%, list=27%, signal=40% |
| 1464 | YAO\_P4\_KO\_VS\_WT\_DN |  | 15 | 0.33 | 0.91 | 0.586 | 0.794 | 1.000 | 4400 | tags=47%, list=33%, signal=70% |
| 1465 | V$TCF11\_01 |  | 163 | 0.21 | 0.91 | 0.673 | 0.794 | 1.000 | 4408 | tags=39%, list=33%, signal=58% |
| 1466 | UVB\_NHEK3\_C1 |  | 56 | 0.24 | 0.91 | 0.633 | 0.796 | 1.000 | 4001 | tags=39%, list=30%, signal=56% |
| 1467 | V$T3R\_Q6 |  | 175 | 0.21 | 0.91 | 0.670 | 0.796 | 1.000 | 1407 | tags=14%, list=11%, signal=16% |
| 1468 | V$HNF1\_Q6 |  | 182 | 0.20 | 0.91 | 0.694 | 0.797 | 1.000 | 5138 | tags=41%, list=39%, signal=66% |
| 1469 | CHR17P12 |  | 18 | 0.32 | 0.91 | 0.598 | 0.798 | 1.000 | 2720 | tags=33%, list=20%, signal=42% |
| 1470 | CHR1P35 |  | 67 | 0.23 | 0.91 | 0.644 | 0.798 | 1.000 | 2778 | tags=28%, list=21%, signal=36% |
| 1471 | GTGCCAA,MIR-96 |  | 235 | 0.20 | 0.91 | 0.708 | 0.798 | 1.000 | 3927 | tags=32%, list=29%, signal=45% |
| 1472 | CHR12Q22 |  | 21 | 0.30 | 0.91 | 0.590 | 0.798 | 1.000 | 3695 | tags=29%, list=28%, signal=39% |
| 1473 | V$RREB1\_01 |  | 138 | 0.21 | 0.91 | 0.672 | 0.798 | 1.000 | 4789 | tags=41%, list=36%, signal=63% |
| 1474 | V$PBX1\_01 |  | 180 | 0.20 | 0.91 | 0.690 | 0.798 | 1.000 | 2622 | tags=22%, list=20%, signal=27% |
| 1475 | EMT\_DN |  | 53 | 0.25 | 0.91 | 0.643 | 0.799 | 1.000 | 2146 | tags=21%, list=16%, signal=25% |
| 1476 | V$HFH3\_01 |  | 139 | 0.21 | 0.90 | 0.683 | 0.801 | 1.000 | 3642 | tags=32%, list=27%, signal=44% |
| 1477 | V$AP4\_Q6 |  | 146 | 0.21 | 0.90 | 0.687 | 0.800 | 1.000 | 2415 | tags=21%, list=18%, signal=26% |
| 1478 | RRAGTTGT\_UNKNOWN |  | 164 | 0.20 | 0.90 | 0.689 | 0.801 | 1.000 | 4053 | tags=35%, list=30%, signal=49% |
| 1479 | V$TEL2\_Q6 |  | 156 | 0.21 | 0.90 | 0.678 | 0.801 | 1.000 | 2250 | tags=20%, list=17%, signal=24% |
| 1480 | V$PAX\_Q6 |  | 170 | 0.20 | 0.90 | 0.716 | 0.800 | 1.000 | 3157 | tags=26%, list=24%, signal=33% |
| 1481 | JECHLINGER\_EMT\_DN |  | 40 | 0.26 | 0.90 | 0.625 | 0.800 | 1.000 | 2146 | tags=23%, list=16%, signal=27% |
| 1482 | YAATNRNNNYNATT\_UNKNOWN |  | 74 | 0.23 | 0.90 | 0.645 | 0.800 | 1.000 | 4157 | tags=38%, list=31%, signal=55% |
| 1483 | HDACI\_COLON\_SUL48HRS\_DN |  | 66 | 0.24 | 0.90 | 0.647 | 0.800 | 1.000 | 3678 | tags=36%, list=28%, signal=50% |
| 1484 | TCTGGAC,MIR-198 |  | 60 | 0.24 | 0.90 | 0.634 | 0.800 | 1.000 | 2188 | tags=22%, list=16%, signal=26% |
| 1485 | GNATENKO\_PLATELET\_UP |  | 44 | 0.25 | 0.90 | 0.640 | 0.799 | 1.000 | 3505 | tags=39%, list=26%, signal=52% |
| 1486 | V$MYB\_Q5\_01 |  | 184 | 0.20 | 0.90 | 0.686 | 0.799 | 1.000 | 3937 | tags=32%, list=30%, signal=45% |
| 1487 | HYPOXIA\_RCC\_NOVHL\_UP |  | 57 | 0.24 | 0.90 | 0.651 | 0.799 | 1.000 | 3085 | tags=33%, list=23%, signal=43% |
| 1488 | HDACI\_COLON\_CLUSTER10 |  | 34 | 0.27 | 0.90 | 0.626 | 0.799 | 1.000 | 3740 | tags=44%, list=28%, signal=61% |
| 1489 | ATAACCT,MIR-154 |  | 49 | 0.25 | 0.90 | 0.638 | 0.799 | 1.000 | 4136 | tags=39%, list=31%, signal=56% |
| 1490 | V$IK2\_01 |  | 191 | 0.20 | 0.90 | 0.707 | 0.798 | 1.000 | 1596 | tags=15%, list=12%, signal=17% |
| 1491 | V$STAT\_01 |  | 174 | 0.20 | 0.90 | 0.700 | 0.798 | 1.000 | 2229 | tags=18%, list=17%, signal=21% |
| 1492 | V$VDR\_Q3 |  | 165 | 0.20 | 0.90 | 0.701 | 0.798 | 1.000 | 4703 | tags=41%, list=35%, signal=63% |
| 1493 | AAGCAAT,MIR-137 |  | 163 | 0.20 | 0.90 | 0.677 | 0.799 | 1.000 | 3937 | tags=32%, list=30%, signal=45% |
| 1494 | V$AR\_02 |  | 26 | 0.28 | 0.90 | 0.620 | 0.799 | 1.000 | 2201 | tags=27%, list=17%, signal=32% |
| 1495 | CHR1Q24 |  | 37 | 0.26 | 0.90 | 0.613 | 0.800 | 1.000 | 4370 | tags=49%, list=33%, signal=72% |
| 1496 | V$MZF1\_02 |  | 166 | 0.20 | 0.90 | 0.673 | 0.800 | 1.000 | 3547 | tags=30%, list=27%, signal=40% |
| 1497 | V$SP1\_Q4\_01 |  | 176 | 0.20 | 0.90 | 0.702 | 0.800 | 1.000 | 3121 | tags=23%, list=23%, signal=29% |
| 1498 | BIOPEPTIDESPATHWAY |  | 38 | 0.26 | 0.90 | 0.615 | 0.800 | 1.000 | 1775 | tags=16%, list=13%, signal=18% |
| 1499 | LVAD\_HEARTFAILURE\_DN |  | 38 | 0.26 | 0.90 | 0.609 | 0.800 | 1.000 | 4247 | tags=42%, list=32%, signal=62% |
| 1500 | V$CDPCR3HD\_01 |  | 164 | 0.20 | 0.90 | 0.712 | 0.804 | 1.000 | 3953 | tags=31%, list=30%, signal=44% |
| 1501 | CHOLESTEROL\_BIOSYNTHESIS |  | 15 | 0.33 | 0.90 | 0.601 | 0.804 | 1.000 | 3031 | tags=40%, list=23%, signal=52% |
| 1502 | UVB\_NHEK2\_UP |  | 68 | 0.23 | 0.90 | 0.641 | 0.804 | 1.000 | 2697 | tags=22%, list=20%, signal=28% |
| 1503 | AATGGAG,MIR-136 |  | 54 | 0.24 | 0.90 | 0.639 | 0.803 | 1.000 | 2961 | tags=28%, list=22%, signal=36% |
| 1504 | UVC\_HIGH\_D8\_DN |  | 29 | 0.28 | 0.90 | 0.639 | 0.806 | 1.000 | 2650 | tags=34%, list=20%, signal=43% |
| 1505 | GTCTACC,MIR-379 |  | 16 | 0.32 | 0.90 | 0.617 | 0.806 | 1.000 | 1564 | tags=19%, list=12%, signal=21% |
| 1506 | LEE\_DENA\_DN |  | 72 | 0.23 | 0.89 | 0.668 | 0.807 | 1.000 | 4418 | tags=39%, list=33%, signal=58% |
| 1507 | V$HNF3B\_01 |  | 146 | 0.20 | 0.89 | 0.725 | 0.807 | 1.000 | 4538 | tags=39%, list=34%, signal=59% |
| 1508 | V$TBP\_01 |  | 166 | 0.20 | 0.89 | 0.710 | 0.808 | 1.000 | 2944 | tags=25%, list=22%, signal=31% |
| 1509 | INSULIN\_SIGNALING |  | 97 | 0.22 | 0.89 | 0.677 | 0.808 | 1.000 | 3460 | tags=29%, list=26%, signal=39% |
| 1510 | GNATENKO\_PLATELET |  | 44 | 0.25 | 0.89 | 0.652 | 0.808 | 1.000 | 3505 | tags=39%, list=26%, signal=52% |
| 1511 | GACAATC,MIR-219 |  | 105 | 0.21 | 0.89 | 0.678 | 0.813 | 1.000 | 3175 | tags=30%, list=24%, signal=38% |
| 1512 | PPARAPATHWAY |  | 52 | 0.24 | 0.89 | 0.638 | 0.813 | 1.000 | 1202 | tags=12%, list=9%, signal=13% |
| 1513 | STAEGE\_EFTS\_UP |  | 21 | 0.29 | 0.89 | 0.614 | 0.813 | 1.000 | 1121 | tags=19%, list=8%, signal=21% |
| 1514 | ATACCTC,MIR-202 |  | 138 | 0.21 | 0.89 | 0.735 | 0.813 | 1.000 | 2766 | tags=23%, list=21%, signal=29% |
| 1515 | CHRXQ22 |  | 45 | 0.25 | 0.89 | 0.649 | 0.813 | 1.000 | 3458 | tags=38%, list=26%, signal=51% |
| 1516 | CHR8P21 |  | 64 | 0.24 | 0.89 | 0.660 | 0.815 | 1.000 | 4791 | tags=39%, list=36%, signal=61% |
| 1517 | V$NKX25\_02 |  | 175 | 0.20 | 0.89 | 0.734 | 0.817 | 1.000 | 4157 | tags=32%, list=31%, signal=46% |
| 1518 | CPR\_LOW\_LIVER\_UP |  | 16 | 0.32 | 0.89 | 0.629 | 0.818 | 1.000 | 2577 | tags=25%, list=19%, signal=31% |
| 1519 | AACWWCAANK\_UNKNOWN |  | 87 | 0.22 | 0.89 | 0.684 | 0.817 | 1.000 | 1365 | tags=15%, list=10%, signal=17% |
| 1520 | V$LYF1\_01 |  | 193 | 0.20 | 0.89 | 0.731 | 0.817 | 1.000 | 2305 | tags=20%, list=17%, signal=23% |
| 1521 | FCER1PATHWAY |  | 37 | 0.26 | 0.89 | 0.645 | 0.820 | 1.000 | 1859 | tags=19%, list=14%, signal=22% |
| 1522 | TTGCACT,MIR-130A,MIR-301,MIR-130B |  | 306 | 0.19 | 0.88 | 0.784 | 0.822 | 1.000 | 3608 | tags=28%, list=27%, signal=37% |
| 1523 | TIS7\_OVEREXP\_DN |  | 17 | 0.31 | 0.88 | 0.605 | 0.822 | 1.000 | 2730 | tags=35%, list=20%, signal=44% |
| 1524 | V$STAT\_Q6 |  | 171 | 0.20 | 0.88 | 0.739 | 0.821 | 1.000 | 2086 | tags=18%, list=16%, signal=21% |
| 1525 | V$HFH8\_01 |  | 154 | 0.20 | 0.88 | 0.723 | 0.822 | 1.000 | 2199 | tags=20%, list=17%, signal=24% |
| 1526 | MONOAMINE\_GPCRS |  | 30 | 0.27 | 0.88 | 0.636 | 0.823 | 1.000 | 5852 | tags=67%, list=44%, signal=119% |
| 1527 | TAGAACC,MIR-182 |  | 27 | 0.28 | 0.88 | 0.655 | 0.825 | 1.000 | 4453 | tags=44%, list=33%, signal=67% |
| 1528 | V$HNF1\_C |  | 165 | 0.20 | 0.88 | 0.733 | 0.824 | 1.000 | 4932 | tags=40%, list=37%, signal=63% |
| 1529 | V$OCT1\_02 |  | 152 | 0.20 | 0.88 | 0.724 | 0.824 | 1.000 | 3799 | tags=32%, list=29%, signal=45% |
| 1530 | CMV\_HCMV\_TIMECOURSE\_1HR\_DN |  | 38 | 0.25 | 0.88 | 0.673 | 0.829 | 1.000 | 5332 | tags=58%, list=40%, signal=96% |
| 1531 | ATGTAGC,MIR-221,MIR-222 |  | 96 | 0.21 | 0.88 | 0.730 | 0.830 | 1.000 | 2015 | tags=21%, list=15%, signal=24% |
| 1532 | CAGCTTT,MIR-320 |  | 192 | 0.20 | 0.88 | 0.745 | 0.832 | 1.000 | 2922 | tags=23%, list=22%, signal=30% |
| 1533 | V$STAT4\_01 |  | 175 | 0.20 | 0.88 | 0.753 | 0.836 | 1.000 | 3765 | tags=31%, list=28%, signal=43% |
| 1534 | CHR22Q12 |  | 83 | 0.22 | 0.88 | 0.715 | 0.835 | 1.000 | 4668 | tags=41%, list=35%, signal=63% |
| 1535 | V$MEF2\_Q6\_01 |  | 171 | 0.20 | 0.88 | 0.757 | 0.837 | 1.000 | 4261 | tags=35%, list=32%, signal=50% |
| 1536 | DOX\_RESIST\_GASTRIC\_DN |  | 17 | 0.30 | 0.87 | 0.652 | 0.840 | 1.000 | 2258 | tags=29%, list=17%, signal=35% |
| 1537 | CATGTAA,MIR-496 |  | 145 | 0.20 | 0.87 | 0.751 | 0.843 | 1.000 | 3804 | tags=32%, list=29%, signal=45% |
| 1538 | ACCAAAG,MIR-9 |  | 375 | 0.18 | 0.87 | 0.834 | 0.844 | 1.000 | 3119 | tags=25%, list=23%, signal=31% |
| 1539 | G\_PROTEIN\_SIGNALING |  | 86 | 0.22 | 0.87 | 0.707 | 0.847 | 1.000 | 5032 | tags=42%, list=38%, signal=67% |
| 1540 | V$AML1\_01 |  | 178 | 0.19 | 0.87 | 0.778 | 0.849 | 1.000 | 4565 | tags=37%, list=34%, signal=56% |
| 1541 | MITOCHONDRIAPATHWAY |  | 20 | 0.30 | 0.87 | 0.651 | 0.849 | 1.000 | 2471 | tags=25%, list=19%, signal=31% |
| 1542 | GAMMA\_ESR\_OLD\_UNREG |  | 24 | 0.28 | 0.87 | 0.673 | 0.849 | 1.000 | 3229 | tags=33%, list=24%, signal=44% |
| 1543 | ZHAN\_MMPC\_SIM\_BC\_AND\_MM |  | 44 | 0.25 | 0.87 | 0.698 | 0.852 | 1.000 | 4085 | tags=41%, list=31%, signal=59% |
| 1544 | RTAAACA\_V$FREAC2\_01 |  | 636 | 0.17 | 0.87 | 0.891 | 0.853 | 1.000 | 4284 | tags=34%, list=32%, signal=47% |
| 1545 | EGFPATHWAY |  | 27 | 0.27 | 0.86 | 0.655 | 0.858 | 1.000 | 1775 | tags=22%, list=13%, signal=26% |
| 1546 | GTGTTGA,MIR-505 |  | 82 | 0.22 | 0.86 | 0.737 | 0.858 | 1.000 | 5090 | tags=38%, list=38%, signal=61% |
| 1547 | CIRCADIAN\_EXERCISE |  | 40 | 0.25 | 0.86 | 0.689 | 0.858 | 1.000 | 1960 | tags=20%, list=15%, signal=23% |
| 1548 | HDACI\_COLON\_CUR24HRS\_UP |  | 36 | 0.25 | 0.86 | 0.702 | 0.860 | 1.000 | 2609 | tags=31%, list=20%, signal=38% |
| 1549 | GKCGCNNNNNNNTGAYG\_UNKNOWN |  | 37 | 0.25 | 0.86 | 0.686 | 0.860 | 1.000 | 1799 | tags=19%, list=14%, signal=22% |
| 1550 | CCCNNNNNNAAGWT\_UNKNOWN |  | 68 | 0.22 | 0.86 | 0.719 | 0.860 | 1.000 | 3212 | tags=26%, list=24%, signal=35% |
| 1551 | V$AML1\_Q6 |  | 178 | 0.19 | 0.86 | 0.794 | 0.860 | 1.000 | 4565 | tags=37%, list=34%, signal=56% |
| 1552 | CACGTTT,MIR-302A |  | 21 | 0.28 | 0.86 | 0.643 | 0.860 | 1.000 | 765 | tags=14%, list=6%, signal=15% |
| 1553 | VEGF\_HUVEC\_UP |  | 15 | 0.31 | 0.86 | 0.676 | 0.866 | 1.000 | 2121 | tags=33%, list=16%, signal=40% |
| 1554 | V$ER\_Q6 |  | 187 | 0.19 | 0.86 | 0.785 | 0.866 | 1.000 | 2860 | tags=25%, list=21%, signal=32% |
| 1555 | TGTTTGY\_V$HNF3\_Q6 |  | 522 | 0.17 | 0.85 | 0.884 | 0.870 | 1.000 | 3801 | tags=29%, list=29%, signal=39% |
| 1556 | V$NF1\_Q6 |  | 176 | 0.19 | 0.85 | 0.799 | 0.870 | 1.000 | 3236 | tags=30%, list=24%, signal=39% |
| 1557 | WELCH\_GATA1 |  | 24 | 0.28 | 0.85 | 0.701 | 0.870 | 1.000 | 3431 | tags=33%, list=26%, signal=45% |
| 1558 | V$SP1\_Q6 |  | 174 | 0.19 | 0.85 | 0.810 | 0.870 | 1.000 | 3121 | tags=22%, list=23%, signal=29% |
| 1559 | TNFR2PATHWAY |  | 18 | 0.29 | 0.85 | 0.685 | 0.872 | 1.000 | 2530 | tags=28%, list=19%, signal=34% |
| 1560 | WALKER\_MM\_SNP\_DIFF |  | 43 | 0.24 | 0.85 | 0.721 | 0.874 | 1.000 | 3433 | tags=33%, list=26%, signal=44% |
| 1561 | GTGGTGA,MIR-197 |  | 51 | 0.23 | 0.85 | 0.738 | 0.875 | 1.000 | 4882 | tags=51%, list=37%, signal=80% |
| 1562 | CALRES\_RHESUS\_DN |  | 59 | 0.22 | 0.85 | 0.742 | 0.875 | 1.000 | 4457 | tags=41%, list=33%, signal=61% |
| 1563 | V$USF\_01 |  | 187 | 0.19 | 0.85 | 0.823 | 0.876 | 1.000 | 1876 | tags=17%, list=14%, signal=20% |
| 1564 | EIF4PATHWAY |  | 24 | 0.27 | 0.85 | 0.694 | 0.879 | 1.000 | 1224 | tags=17%, list=9%, signal=18% |
| 1565 | V$TITF1\_Q3 |  | 172 | 0.19 | 0.85 | 0.813 | 0.878 | 1.000 | 3646 | tags=27%, list=27%, signal=37% |
| 1566 | GHPATHWAY |  | 27 | 0.27 | 0.85 | 0.704 | 0.878 | 1.000 | 3460 | tags=37%, list=26%, signal=50% |
| 1567 | HDACI\_COLON\_BUT30MIN\_DN |  | 32 | 0.26 | 0.85 | 0.701 | 0.879 | 1.000 | 3884 | tags=41%, list=29%, signal=57% |
| 1568 | V$FREAC4\_01 |  | 98 | 0.21 | 0.85 | 0.781 | 0.879 | 1.000 | 3561 | tags=30%, list=27%, signal=40% |
| 1569 | TCTCTCC,MIR-185 |  | 93 | 0.21 | 0.85 | 0.775 | 0.879 | 1.000 | 3911 | tags=29%, list=29%, signal=41% |
| 1570 | V$AP2\_Q3 |  | 182 | 0.19 | 0.85 | 0.813 | 0.880 | 1.000 | 4401 | tags=38%, list=33%, signal=56% |
| 1571 | UEDA\_MOUSE\_SCN |  | 74 | 0.21 | 0.85 | 0.744 | 0.879 | 1.000 | 2807 | tags=22%, list=21%, signal=27% |
| 1572 | V$POU3F2\_01 |  | 71 | 0.22 | 0.85 | 0.772 | 0.880 | 1.000 | 2957 | tags=28%, list=22%, signal=36% |
| 1573 | V$HNF4\_01\_B |  | 181 | 0.19 | 0.85 | 0.843 | 0.879 | 1.000 | 3927 | tags=32%, list=29%, signal=45% |
| 1574 | V$GCM\_Q2 |  | 162 | 0.19 | 0.84 | 0.807 | 0.881 | 1.000 | 1506 | tags=14%, list=11%, signal=16% |
| 1575 | TCCCCAC,MIR-491 |  | 49 | 0.23 | 0.84 | 0.757 | 0.881 | 1.000 | 5968 | tags=51%, list=45%, signal=92% |
| 1576 | V$PAX4\_01 |  | 193 | 0.19 | 0.84 | 0.831 | 0.883 | 1.000 | 3540 | tags=29%, list=27%, signal=39% |
| 1577 | ADIP\_VS\_PREADIP\_DN |  | 37 | 0.25 | 0.84 | 0.734 | 0.886 | 1.000 | 2546 | tags=27%, list=19%, signal=33% |
| 1578 | V$GATA4\_Q3 |  | 167 | 0.19 | 0.84 | 0.825 | 0.885 | 1.000 | 3212 | tags=24%, list=24%, signal=31% |
| 1579 | V$YY1\_01 |  | 175 | 0.19 | 0.84 | 0.836 | 0.885 | 1.000 | 2227 | tags=17%, list=17%, signal=20% |
| 1580 | V$GFI1\_01 |  | 183 | 0.19 | 0.84 | 0.827 | 0.886 | 1.000 | 2965 | tags=21%, list=22%, signal=27% |
| 1581 | GREENBAUM\_E2A\_DN |  | 17 | 0.29 | 0.84 | 0.692 | 0.886 | 1.000 | 2059 | tags=24%, list=15%, signal=28% |
| 1582 | V$AP2\_Q6 |  | 185 | 0.19 | 0.84 | 0.852 | 0.887 | 1.000 | 3796 | tags=29%, list=28%, signal=40% |
| 1583 | TPA\_RESIST\_MIDDLE\_UP |  | 48 | 0.23 | 0.84 | 0.745 | 0.887 | 1.000 | 4627 | tags=40%, list=35%, signal=60% |
| 1584 | TSA\_PANC50\_UP |  | 37 | 0.24 | 0.84 | 0.736 | 0.887 | 1.000 | 3819 | tags=38%, list=29%, signal=53% |
| 1585 | GGATTA\_V$PITX2\_Q2 |  | 399 | 0.17 | 0.84 | 0.918 | 0.889 | 1.000 | 4157 | tags=33%, list=31%, signal=46% |
| 1586 | ARGGGTTAA\_UNKNOWN |  | 91 | 0.21 | 0.84 | 0.790 | 0.888 | 1.000 | 3937 | tags=33%, list=30%, signal=46% |
| 1587 | TCCAGAT,MIR-516-5P |  | 83 | 0.21 | 0.84 | 0.779 | 0.888 | 1.000 | 2438 | tags=24%, list=18%, signal=29% |
| 1588 | AT1RPATHWAY |  | 34 | 0.25 | 0.84 | 0.722 | 0.888 | 1.000 | 1775 | tags=18%, list=13%, signal=20% |
| 1589 | CHR9Q34 |  | 142 | 0.20 | 0.84 | 0.844 | 0.888 | 1.000 | 3486 | tags=25%, list=26%, signal=34% |
| 1590 | GENOTOXINS\_4HRS\_DISCR |  | 33 | 0.26 | 0.84 | 0.706 | 0.888 | 1.000 | 4627 | tags=48%, list=35%, signal=74% |
| 1591 | V$CDP\_02 |  | 75 | 0.21 | 0.84 | 0.774 | 0.888 | 1.000 | 4335 | tags=36%, list=33%, signal=53% |
| 1592 | PDGFPATHWAY |  | 27 | 0.27 | 0.84 | 0.717 | 0.887 | 1.000 | 1775 | tags=22%, list=13%, signal=26% |
| 1593 | ATGTACA,MIR-493 |  | 242 | 0.18 | 0.84 | 0.880 | 0.887 | 1.000 | 3494 | tags=25%, list=26%, signal=34% |
| 1594 | CHR16Q12 |  | 29 | 0.26 | 0.84 | 0.708 | 0.887 | 1.000 | 3467 | tags=34%, list=26%, signal=47% |
| 1595 | BRCA2\_BRCA1\_DN |  | 40 | 0.24 | 0.84 | 0.743 | 0.886 | 1.000 | 2619 | tags=28%, list=20%, signal=34% |
| 1596 | V$LMO2COM\_01 |  | 160 | 0.19 | 0.84 | 0.840 | 0.887 | 1.000 | 4284 | tags=36%, list=32%, signal=53% |
| 1597 | GAANYNYGACNY\_UNKNOWN |  | 54 | 0.22 | 0.83 | 0.735 | 0.887 | 1.000 | 1736 | tags=17%, list=13%, signal=19% |
| 1598 | CHR2Q11 |  | 31 | 0.25 | 0.83 | 0.705 | 0.887 | 1.000 | 3308 | tags=35%, list=25%, signal=47% |
| 1599 | V$DR4\_Q2 |  | 170 | 0.19 | 0.83 | 0.821 | 0.887 | 1.000 | 2986 | tags=24%, list=22%, signal=30% |
| 1600 | V$RFX1\_01 |  | 157 | 0.19 | 0.83 | 0.841 | 0.887 | 1.000 | 3339 | tags=25%, list=25%, signal=33% |
| 1601 | CHRXQ21 |  | 29 | 0.25 | 0.83 | 0.729 | 0.892 | 1.000 | 4610 | tags=38%, list=35%, signal=58% |
| 1602 | COCAINE\_BRAIN\_5D\_UP |  | 60 | 0.22 | 0.83 | 0.753 | 0.893 | 1.000 | 1897 | tags=17%, list=14%, signal=19% |
| 1603 | V$PR\_01 |  | 91 | 0.21 | 0.83 | 0.806 | 0.895 | 1.000 | 2201 | tags=19%, list=17%, signal=22% |
| 1604 | TNCATNTCCYR\_UNKNOWN |  | 84 | 0.21 | 0.83 | 0.789 | 0.897 | 1.000 | 2488 | tags=19%, list=19%, signal=23% |
| 1605 | INNEREAR\_UP |  | 38 | 0.24 | 0.83 | 0.751 | 0.897 | 1.000 | 2361 | tags=24%, list=18%, signal=29% |
| 1606 | CMV\_HCMV\_6HRS\_UP |  | 25 | 0.26 | 0.83 | 0.734 | 0.897 | 1.000 | 930 | tags=12%, list=7%, signal=13% |
| 1607 | CREBPATHWAY |  | 26 | 0.26 | 0.83 | 0.725 | 0.897 | 1.000 | 3621 | tags=35%, list=27%, signal=47% |
| 1608 | GGGGCCC,MIR-296 |  | 50 | 0.23 | 0.83 | 0.756 | 0.899 | 1.000 | 1575 | tags=12%, list=12%, signal=14% |
| 1609 | V$ATF6\_01 |  | 88 | 0.21 | 0.83 | 0.800 | 0.898 | 1.000 | 1681 | tags=15%, list=13%, signal=17% |
| 1610 | CHR1Q23 |  | 52 | 0.22 | 0.83 | 0.779 | 0.898 | 1.000 | 655 | tags=10%, list=5%, signal=10% |
| 1611 | V$E4F1\_Q6 |  | 190 | 0.18 | 0.83 | 0.854 | 0.898 | 1.000 | 3601 | tags=30%, list=27%, signal=41% |
| 1612 | HDACI\_COLON\_TSA2HRS\_DN |  | 20 | 0.28 | 0.82 | 0.711 | 0.898 | 1.000 | 2354 | tags=30%, list=18%, signal=36% |
| 1613 | ACCAATC,MIR-509 |  | 34 | 0.24 | 0.82 | 0.725 | 0.898 | 1.000 | 3025 | tags=26%, list=23%, signal=34% |
| 1614 | V$OCT1\_06 |  | 184 | 0.18 | 0.82 | 0.855 | 0.898 | 1.000 | 3565 | tags=27%, list=27%, signal=37% |
| 1615 | V$CACBINDINGPROTEIN\_Q6 |  | 165 | 0.19 | 0.82 | 0.845 | 0.898 | 1.000 | 3701 | tags=30%, list=28%, signal=41% |
| 1616 | ST\_ERK1\_ERK2\_MAPK\_PATHWAY |  | 29 | 0.26 | 0.82 | 0.716 | 0.897 | 1.000 | 2517 | tags=31%, list=19%, signal=38% |
| 1617 | ATRIA\_UP |  | 195 | 0.18 | 0.82 | 0.873 | 0.897 | 1.000 | 2288 | tags=19%, list=17%, signal=23% |
| 1618 | AAGCACA,MIR-218 |  | 293 | 0.18 | 0.82 | 0.911 | 0.897 | 1.000 | 4030 | tags=31%, list=30%, signal=44% |
| 1619 | NUMATA\_G\_CSF\_DIFF |  | 18 | 0.28 | 0.82 | 0.701 | 0.899 | 1.000 | 1161 | tags=17%, list=9%, signal=18% |
| 1620 | GCGSCMNTTT\_UNKNOWN |  | 52 | 0.22 | 0.82 | 0.780 | 0.900 | 1.000 | 2954 | tags=27%, list=22%, signal=34% |
| 1621 | CHR6Q24 |  | 25 | 0.26 | 0.82 | 0.732 | 0.900 | 1.000 | 3765 | tags=36%, list=28%, signal=50% |
| 1622 | MAPK\_CASCADE |  | 29 | 0.25 | 0.82 | 0.741 | 0.901 | 1.000 | 2679 | tags=28%, list=20%, signal=34% |
| 1623 | CELL\_ADHESION\_RECEPTOR\_ACTIVITY |  | 31 | 0.25 | 0.82 | 0.721 | 0.900 | 1.000 | 3076 | tags=29%, list=23%, signal=38% |
| 1624 | BILE\_ACID\_BIOSYNTHESIS |  | 24 | 0.26 | 0.82 | 0.732 | 0.900 | 1.000 | 4462 | tags=38%, list=33%, signal=56% |
| 1625 | V$EVI1\_04 |  | 168 | 0.19 | 0.82 | 0.874 | 0.901 | 1.000 | 3864 | tags=28%, list=29%, signal=39% |
| 1626 | V$STAT5A\_01 |  | 178 | 0.18 | 0.82 | 0.863 | 0.902 | 1.000 | 2265 | tags=17%, list=17%, signal=21% |
| 1627 | V$EFC\_Q6 |  | 185 | 0.18 | 0.82 | 0.890 | 0.902 | 1.000 | 2788 | tags=23%, list=21%, signal=28% |
| 1628 | ROSS\_FAB\_M7 |  | 69 | 0.21 | 0.82 | 0.821 | 0.902 | 1.000 | 4888 | tags=38%, list=37%, signal=59% |
| 1629 | V$STAT3\_01 |  | 16 | 0.29 | 0.82 | 0.709 | 0.902 | 1.000 | 3036 | tags=31%, list=23%, signal=40% |
| 1630 | TRYPTOPHAN\_METABOLISM |  | 53 | 0.23 | 0.81 | 0.762 | 0.906 | 1.000 | 4605 | tags=40%, list=35%, signal=60% |
| 1631 | CACTGCC,MIR-34A,MIR-34C,MIR-449 |  | 211 | 0.18 | 0.81 | 0.871 | 0.906 | 1.000 | 3608 | tags=29%, list=27%, signal=39% |
| 1632 | IL1\_CORNEA\_DN |  | 74 | 0.21 | 0.81 | 0.790 | 0.906 | 1.000 | 3107 | tags=27%, list=23%, signal=35% |
| 1633 | AGGTGCA,MIR-500 |  | 73 | 0.21 | 0.81 | 0.799 | 0.907 | 1.000 | 2596 | tags=19%, list=19%, signal=24% |
| 1634 | TGAGATT,MIR-216 |  | 77 | 0.21 | 0.81 | 0.822 | 0.909 | 1.000 | 2456 | tags=19%, list=18%, signal=24% |
| 1635 | TIDPATHWAY |  | 18 | 0.29 | 0.81 | 0.741 | 0.908 | 1.000 | 3857 | tags=33%, list=29%, signal=47% |
| 1636 | INTRINSICPATHWAY |  | 22 | 0.27 | 0.81 | 0.765 | 0.908 | 1.000 | 4918 | tags=41%, list=37%, signal=65% |
| 1637 | AAAYRNCTG\_UNKNOWN |  | 262 | 0.17 | 0.81 | 0.918 | 0.909 | 1.000 | 1703 | tags=13%, list=13%, signal=15% |
| 1638 | V$PAX4\_02 |  | 160 | 0.18 | 0.81 | 0.896 | 0.909 | 1.000 | 3441 | tags=24%, list=26%, signal=32% |
| 1639 | RIBAVIRIN\_RSV\_UP |  | 22 | 0.27 | 0.81 | 0.765 | 0.909 | 1.000 | 2768 | tags=23%, list=21%, signal=29% |
| 1640 | WILLERT\_WNT\_NCCIT\_ALL\_UP |  | 23 | 0.26 | 0.81 | 0.744 | 0.909 | 1.000 | 2043 | tags=26%, list=15%, signal=31% |
| 1641 | CTCCAAG,MIR-432 |  | 52 | 0.22 | 0.81 | 0.777 | 0.908 | 1.000 | 4213 | tags=35%, list=32%, signal=50% |
| 1642 | CHR11P11 |  | 37 | 0.24 | 0.81 | 0.758 | 0.908 | 1.000 | 5769 | tags=54%, list=43%, signal=95% |
| 1643 | GGGTGGRR\_V$PAX4\_03 |  | 924 | 0.16 | 0.81 | 0.991 | 0.907 | 1.000 | 3573 | tags=27%, list=27%, signal=34% |
| 1644 | CMV\_HCMV\_TIMECOURSE\_20HRS\_DN |  | 40 | 0.23 | 0.81 | 0.765 | 0.908 | 1.000 | 5723 | tags=58%, list=43%, signal=101% |
| 1645 | UVC\_TTD\_8HR\_UP |  | 23 | 0.27 | 0.81 | 0.739 | 0.909 | 1.000 | 4056 | tags=39%, list=30%, signal=56% |
| 1646 | ACTGCCT,MIR-34B |  | 172 | 0.18 | 0.81 | 0.902 | 0.908 | 1.000 | 2188 | tags=19%, list=16%, signal=22% |
| 1647 | PARK\_HSC\_VS\_MPP\_UP |  | 16 | 0.29 | 0.81 | 0.737 | 0.908 | 1.000 | 2713 | tags=38%, list=20%, signal=47% |
| 1648 | CHR2Q35 |  | 35 | 0.24 | 0.81 | 0.757 | 0.908 | 1.000 | 5377 | tags=51%, list=40%, signal=86% |
| 1649 | ZHAN\_MMPC\_PC |  | 22 | 0.27 | 0.81 | 0.743 | 0.908 | 1.000 | 5341 | tags=73%, list=40%, signal=121% |
| 1650 | AGCYRWTTC\_UNKNOWN |  | 81 | 0.20 | 0.81 | 0.817 | 0.908 | 1.000 | 2386 | tags=20%, list=18%, signal=24% |
| 1651 | G13\_SIGNALING\_PATHWAY |  | 36 | 0.24 | 0.81 | 0.769 | 0.908 | 1.000 | 3275 | tags=31%, list=25%, signal=40% |
| 1652 | ET743\_SARCOMA\_72HRS\_UP |  | 62 | 0.21 | 0.81 | 0.814 | 0.908 | 1.000 | 2363 | tags=23%, list=18%, signal=27% |
| 1653 | ET743\_SARCOMA\_6HRS\_UP |  | 30 | 0.24 | 0.80 | 0.759 | 0.911 | 1.000 | 4338 | tags=40%, list=33%, signal=59% |
| 1654 | BYSTRYKH\_HSC\_BRAIN\_CIS\_GLOCUS |  | 41 | 0.23 | 0.80 | 0.772 | 0.912 | 1.000 | 2506 | tags=27%, list=19%, signal=33% |
| 1655 | V$STAT5B\_01 |  | 180 | 0.18 | 0.80 | 0.911 | 0.911 | 1.000 | 3276 | tags=23%, list=25%, signal=30% |
| 1656 | V$POU1F1\_Q6 |  | 157 | 0.18 | 0.80 | 0.896 | 0.915 | 1.000 | 4337 | tags=36%, list=33%, signal=53% |
| 1657 | CHR9Q33 |  | 34 | 0.24 | 0.80 | 0.799 | 0.914 | 1.000 | 3706 | tags=29%, list=28%, signal=41% |
| 1658 | V$AR\_01 |  | 98 | 0.20 | 0.80 | 0.863 | 0.917 | 1.000 | 2191 | tags=16%, list=16%, signal=19% |
| 1659 | RADAEVA\_IFNA\_UP |  | 50 | 0.22 | 0.80 | 0.822 | 0.919 | 1.000 | 3137 | tags=26%, list=24%, signal=34% |
| 1660 | PITUITARY\_FETAL\_UP |  | 15 | 0.30 | 0.80 | 0.777 | 0.919 | 1.000 | 2025 | tags=27%, list=15%, signal=31% |
| 1661 | GPCRDB\_OTHER |  | 49 | 0.22 | 0.79 | 0.825 | 0.922 | 1.000 | 2581 | tags=24%, list=19%, signal=30% |
| 1662 | JISON\_SICKLE\_CELL |  | 31 | 0.24 | 0.79 | 0.784 | 0.921 | 1.000 | 3742 | tags=39%, list=28%, signal=54% |
| 1663 | V$AFP1\_Q6 |  | 171 | 0.18 | 0.79 | 0.910 | 0.921 | 1.000 | 2277 | tags=19%, list=17%, signal=22% |
| 1664 | V$GATA\_C |  | 194 | 0.18 | 0.79 | 0.927 | 0.925 | 1.000 | 2510 | tags=18%, list=19%, signal=22% |
| 1665 | V$STAT6\_02 |  | 171 | 0.18 | 0.79 | 0.925 | 0.924 | 1.000 | 4250 | tags=37%, list=32%, signal=54% |
| 1666 | TPA\_RESIST\_EARLY\_UP |  | 30 | 0.24 | 0.79 | 0.794 | 0.925 | 1.000 | 1963 | tags=23%, list=15%, signal=27% |
| 1667 | ELECTRON\_TRANSPORT |  | 69 | 0.21 | 0.79 | 0.842 | 0.925 | 1.000 | 2849 | tags=26%, list=21%, signal=33% |
| 1668 | V$MMEF2\_Q6 |  | 194 | 0.18 | 0.79 | 0.919 | 0.925 | 1.000 | 3909 | tags=32%, list=29%, signal=45% |
| 1669 | V$SP1\_01 |  | 172 | 0.18 | 0.79 | 0.923 | 0.925 | 1.000 | 1121 | tags=10%, list=8%, signal=11% |
| 1670 | CTCNANGTGNY\_UNKNOWN |  | 58 | 0.21 | 0.79 | 0.824 | 0.925 | 1.000 | 4273 | tags=43%, list=32%, signal=63% |
| 1671 | YGCGYRCGC\_UNKNOWN |  | 205 | 0.17 | 0.79 | 0.922 | 0.926 | 1.000 | 4253 | tags=30%, list=32%, signal=44% |
| 1672 | TGTGTGA,MIR-377 |  | 151 | 0.18 | 0.79 | 0.915 | 0.926 | 1.000 | 4406 | tags=34%, list=33%, signal=51% |
| 1673 | V$AHR\_01 |  | 48 | 0.22 | 0.79 | 0.816 | 0.928 | 1.000 | 891 | tags=10%, list=7%, signal=11% |
| 1674 | V$PPARA\_01 |  | 27 | 0.25 | 0.79 | 0.788 | 0.928 | 1.000 | 5265 | tags=56%, list=40%, signal=92% |
| 1675 | ADIPOGENESIS\_HMSC\_CLASS3\_UP |  | 60 | 0.21 | 0.79 | 0.844 | 0.928 | 1.000 | 2606 | tags=22%, list=20%, signal=27% |
| 1676 | NI2\_MOUSE\_DN |  | 41 | 0.22 | 0.78 | 0.823 | 0.929 | 1.000 | 1880 | tags=17%, list=14%, signal=20% |
| 1677 | GTGTCAA,MIR-514 |  | 52 | 0.21 | 0.78 | 0.838 | 0.930 | 1.000 | 2584 | tags=19%, list=19%, signal=24% |
| 1678 | TAVOR\_CEBP\_DN |  | 31 | 0.24 | 0.78 | 0.803 | 0.933 | 1.000 | 1025 | tags=13%, list=8%, signal=14% |
| 1679 | GTGGGTGK\_UNKNOWN |  | 209 | 0.17 | 0.78 | 0.948 | 0.933 | 1.000 | 2122 | tags=14%, list=16%, signal=17% |
| 1680 | LEE\_MYC\_DN |  | 59 | 0.21 | 0.78 | 0.853 | 0.933 | 1.000 | 3220 | tags=22%, list=24%, signal=29% |
| 1681 | FSH\_OVARY\_MCV152\_DN |  | 46 | 0.22 | 0.78 | 0.829 | 0.933 | 1.000 | 4762 | tags=43%, list=36%, signal=67% |
| 1682 | BYSTRYKH\_HSC\_CIS\_GLOCUS |  | 91 | 0.19 | 0.78 | 0.894 | 0.933 | 1.000 | 4106 | tags=38%, list=31%, signal=55% |
| 1683 | CHR2Q31 |  | 48 | 0.21 | 0.78 | 0.839 | 0.933 | 1.000 | 3992 | tags=35%, list=30%, signal=50% |
| 1684 | V$STAT1\_03 |  | 172 | 0.18 | 0.78 | 0.931 | 0.933 | 1.000 | 1932 | tags=15%, list=15%, signal=17% |
| 1685 | PENG\_LEUCINE\_UP |  | 102 | 0.19 | 0.78 | 0.881 | 0.933 | 1.000 | 3262 | tags=28%, list=24%, signal=37% |
| 1686 | V$BRN2\_01 |  | 162 | 0.18 | 0.78 | 0.931 | 0.935 | 1.000 | 3358 | tags=25%, list=25%, signal=33% |
| 1687 | NOUZOVA\_CPG\_METHLTD |  | 49 | 0.22 | 0.78 | 0.850 | 0.935 | 1.000 | 2992 | tags=27%, list=22%, signal=34% |
| 1688 | CHR8P23 |  | 27 | 0.24 | 0.78 | 0.811 | 0.934 | 1.000 | 2050 | tags=19%, list=15%, signal=22% |
| 1689 | BYSTRYKH\_HSC\_BRAIN\_TRANS\_GLOCUS |  | 143 | 0.18 | 0.78 | 0.930 | 0.934 | 1.000 | 3026 | tags=23%, list=23%, signal=30% |
| 1690 | ST\_GRANULE\_CELL\_SURVIVAL\_PATHWAY |  | 27 | 0.24 | 0.78 | 0.811 | 0.934 | 1.000 | 2515 | tags=22%, list=19%, signal=27% |
| 1691 | CHRXP11 |  | 104 | 0.19 | 0.77 | 0.895 | 0.935 | 1.000 | 4737 | tags=36%, list=36%, signal=55% |
| 1692 | RACCYCDPATHWAY |  | 22 | 0.25 | 0.77 | 0.793 | 0.935 | 1.000 | 3460 | tags=36%, list=26%, signal=49% |
| 1693 | V$PR\_02 |  | 95 | 0.19 | 0.77 | 0.896 | 0.935 | 1.000 | 2579 | tags=20%, list=19%, signal=25% |
| 1694 | PASSERINI\_TRANSCRIPTION |  | 71 | 0.20 | 0.77 | 0.872 | 0.935 | 1.000 | 2554 | tags=21%, list=19%, signal=26% |
| 1695 | TGATTTRY\_V$GFI1\_01 |  | 212 | 0.17 | 0.77 | 0.956 | 0.936 | 1.000 | 4408 | tags=33%, list=33%, signal=48% |
| 1696 | P53GENES\_ALL |  | 17 | 0.27 | 0.77 | 0.806 | 0.936 | 1.000 | 1966 | tags=24%, list=15%, signal=28% |
| 1697 | HADDAD\_HSC\_CD7\_DN |  | 84 | 0.19 | 0.77 | 0.900 | 0.937 | 1.000 | 2058 | tags=18%, list=15%, signal=21% |
| 1698 | GCTCTTG,MIR-335 |  | 63 | 0.20 | 0.77 | 0.865 | 0.936 | 1.000 | 3216 | tags=27%, list=24%, signal=35% |
| 1699 | V$PIT1\_Q6 |  | 154 | 0.17 | 0.77 | 0.955 | 0.937 | 1.000 | 4557 | tags=34%, list=34%, signal=51% |
| 1700 | GGARNTKYCCA\_UNKNOWN |  | 53 | 0.21 | 0.77 | 0.881 | 0.937 | 1.000 | 4099 | tags=32%, list=31%, signal=46% |
| 1701 | O6BG\_RESIST\_MEDULLOBLASTOMA\_UP |  | 23 | 0.25 | 0.77 | 0.820 | 0.937 | 1.000 | 2350 | tags=26%, list=18%, signal=32% |
| 1702 | V$HNF4\_01 |  | 188 | 0.17 | 0.77 | 0.957 | 0.936 | 1.000 | 4258 | tags=33%, list=32%, signal=48% |
| 1703 | IFNALPHA\_NL\_HCC\_UP |  | 18 | 0.27 | 0.77 | 0.799 | 0.937 | 1.000 | 3118 | tags=33%, list=23%, signal=43% |
| 1704 | EPOPATHWAY |  | 19 | 0.26 | 0.77 | 0.796 | 0.937 | 1.000 | 2391 | tags=21%, list=18%, signal=26% |
| 1705 | ELECTRON\_TRANSPORTER\_ACTIVITY |  | 106 | 0.18 | 0.77 | 0.922 | 0.938 | 1.000 | 2904 | tags=25%, list=22%, signal=31% |
| 1706 | NKCELLSPATHWAY |  | 18 | 0.27 | 0.77 | 0.805 | 0.937 | 1.000 | 4563 | tags=44%, list=34%, signal=68% |
| 1707 | OLDWERNER\_FIBRO\_UP |  | 21 | 0.26 | 0.76 | 0.820 | 0.939 | 1.000 | 2463 | tags=24%, list=18%, signal=29% |
| 1708 | CAGTCAC,MIR-134 |  | 39 | 0.22 | 0.76 | 0.832 | 0.939 | 1.000 | 3459 | tags=33%, list=26%, signal=45% |
| 1709 | FATTY\_ACID\_DEGRADATION |  | 24 | 0.25 | 0.76 | 0.814 | 0.939 | 1.000 | 2735 | tags=33%, list=21%, signal=42% |
| 1710 | HADDAD\_CD45CD7\_PLUS\_VS\_MINUS\_DN |  | 84 | 0.19 | 0.76 | 0.893 | 0.939 | 1.000 | 2058 | tags=18%, list=15%, signal=21% |
| 1711 | HUMAN\_TISSUE\_LIVER |  | 36 | 0.22 | 0.76 | 0.850 | 0.939 | 1.000 | 4984 | tags=36%, list=37%, signal=58% |
| 1712 | TTTNNANAGCYR\_UNKNOWN |  | 106 | 0.18 | 0.76 | 0.911 | 0.941 | 1.000 | 2510 | tags=18%, list=19%, signal=22% |
| 1713 | GLYCEROPHOSPHOLIPID\_METABOLISM |  | 48 | 0.21 | 0.76 | 0.856 | 0.941 | 1.000 | 3806 | tags=29%, list=29%, signal=41% |
| 1714 | V$FXR\_IR1\_Q6 |  | 80 | 0.19 | 0.76 | 0.899 | 0.943 | 1.000 | 4078 | tags=34%, list=31%, signal=48% |
| 1715 | ERK5PATHWAY |  | 17 | 0.27 | 0.76 | 0.783 | 0.944 | 1.000 | 5366 | tags=53%, list=40%, signal=89% |
| 1716 | FALT\_BCLL\_IG\_MUTATED\_VS\_WT\_UP |  | 47 | 0.21 | 0.75 | 0.881 | 0.946 | 1.000 | 2761 | tags=26%, list=21%, signal=32% |
| 1717 | CTGAGCC,MIR-24 |  | 170 | 0.17 | 0.75 | 0.966 | 0.947 | 1.000 | 2550 | tags=19%, list=19%, signal=23% |
| 1718 | STATIN\_PATHWAY\_PHARMGKB |  | 17 | 0.26 | 0.75 | 0.810 | 0.950 | 1.000 | 5398 | tags=41%, list=41%, signal=69% |
| 1719 | MAPKPATHWAY |  | 84 | 0.19 | 0.75 | 0.915 | 0.952 | 1.000 | 3460 | tags=27%, list=26%, signal=37% |
| 1720 | TAGCTTT,MIR-9 |  | 179 | 0.17 | 0.75 | 0.967 | 0.953 | 1.000 | 2639 | tags=20%, list=20%, signal=24% |
| 1721 | HDACI\_COLON\_SUL16HRS\_UP |  | 37 | 0.22 | 0.75 | 0.862 | 0.953 | 1.000 | 2597 | tags=27%, list=19%, signal=33% |
| 1722 | PHENYLALANINE\_METABOLISM |  | 21 | 0.25 | 0.75 | 0.836 | 0.953 | 1.000 | 4199 | tags=43%, list=32%, signal=62% |
| 1723 | V$STAT5A\_04 |  | 132 | 0.17 | 0.74 | 0.958 | 0.952 | 1.000 | 4538 | tags=36%, list=34%, signal=53% |
| 1724 | CREB\_BRAIN\_8WKS\_DN |  | 44 | 0.21 | 0.74 | 0.860 | 0.952 | 1.000 | 1101 | tags=14%, list=8%, signal=15% |
| 1725 | V$NKX22\_01 |  | 124 | 0.18 | 0.74 | 0.949 | 0.954 | 1.000 | 1441 | tags=11%, list=11%, signal=13% |
| 1726 | CHR1Q21 |  | 162 | 0.17 | 0.74 | 0.971 | 0.955 | 1.000 | 4623 | tags=40%, list=35%, signal=61% |
| 1727 | CHR7Q11 |  | 54 | 0.20 | 0.74 | 0.888 | 0.956 | 1.000 | 3216 | tags=22%, list=24%, signal=29% |
| 1728 | CITRATE\_CYCLE\_TCA\_CYCLE |  | 20 | 0.26 | 0.74 | 0.836 | 0.956 | 1.000 | 5183 | tags=50%, list=39%, signal=82% |
| 1729 | IGF1MTORPATHWAY |  | 20 | 0.25 | 0.74 | 0.845 | 0.957 | 1.000 | 2127 | tags=15%, list=16%, signal=18% |
| 1730 | CCATCCA,MIR-432 |  | 44 | 0.21 | 0.74 | 0.878 | 0.958 | 1.000 | 2971 | tags=23%, list=22%, signal=29% |
| 1731 | HOX\_GENES |  | 53 | 0.20 | 0.73 | 0.886 | 0.960 | 1.000 | 4305 | tags=38%, list=32%, signal=56% |
| 1732 | V$YY1\_Q6 |  | 162 | 0.17 | 0.73 | 0.970 | 0.960 | 1.000 | 4165 | tags=31%, list=31%, signal=45% |
| 1733 | V$NFMUE1\_Q6 |  | 174 | 0.16 | 0.73 | 0.980 | 0.960 | 1.000 | 2621 | tags=18%, list=20%, signal=22% |
| 1734 | PROPANOATE\_METABOLISM |  | 30 | 0.22 | 0.73 | 0.872 | 0.962 | 1.000 | 2904 | tags=30%, list=22%, signal=38% |
| 1735 | NGFPATHWAY |  | 19 | 0.25 | 0.73 | 0.832 | 0.965 | 1.000 | 3811 | tags=37%, list=29%, signal=52% |
| 1736 | TTGGAGA,MIR-515-5P,MIR-519E |  | 110 | 0.17 | 0.72 | 0.970 | 0.968 | 1.000 | 3407 | tags=22%, list=26%, signal=29% |
| 1737 | UV-4NQO\_FIBRO\_DN |  | 28 | 0.22 | 0.72 | 0.881 | 0.968 | 1.000 | 214 | tags=7%, list=2%, signal=7% |
| 1738 | WCTCNATGGY\_UNKNOWN |  | 49 | 0.20 | 0.72 | 0.907 | 0.967 | 1.000 | 3508 | tags=29%, list=26%, signal=39% |
| 1739 | PTDINSPATHWAY |  | 20 | 0.24 | 0.72 | 0.857 | 0.967 | 1.000 | 5366 | tags=50%, list=40%, signal=84% |
| 1740 | GAGCTGG,MIR-337 |  | 116 | 0.17 | 0.72 | 0.955 | 0.968 | 1.000 | 4717 | tags=38%, list=35%, signal=58% |
| 1741 | TOB1PATHWAY |  | 17 | 0.26 | 0.72 | 0.842 | 0.967 | 1.000 | 4156 | tags=41%, list=31%, signal=60% |
| 1742 | GUO\_HEX\_DN |  | 54 | 0.19 | 0.72 | 0.904 | 0.967 | 1.000 | 2391 | tags=19%, list=18%, signal=22% |
| 1743 | GSK3PATHWAY |  | 25 | 0.23 | 0.72 | 0.886 | 0.967 | 1.000 | 2074 | tags=16%, list=16%, signal=19% |
| 1744 | UVC\_HIGH\_D9\_DN |  | 24 | 0.24 | 0.71 | 0.854 | 0.970 | 1.000 | 2189 | tags=25%, list=16%, signal=30% |
| 1745 | V$GATA2\_01 |  | 78 | 0.18 | 0.71 | 0.945 | 0.970 | 1.000 | 4017 | tags=28%, list=30%, signal=40% |
| 1746 | GALE\_FLT3ANDAPL\_DN |  | 18 | 0.25 | 0.71 | 0.860 | 0.969 | 1.000 | 3336 | tags=33%, list=25%, signal=44% |
| 1747 | GAMMA\_HEXACHLOROCYCLOHEXANE\_DEGRADATION |  | 28 | 0.22 | 0.71 | 0.887 | 0.969 | 1.000 | 5806 | tags=54%, list=44%, signal=95% |
| 1748 | GH\_EXOGENOUS\_LATE\_UP |  | 57 | 0.19 | 0.71 | 0.938 | 0.971 | 1.000 | 3841 | tags=28%, list=29%, signal=39% |
| 1749 | GGAANCGGAANY\_UNKNOWN |  | 73 | 0.18 | 0.71 | 0.940 | 0.972 | 1.000 | 4396 | tags=36%, list=33%, signal=53% |
| 1750 | V$HNF4\_DR1\_Q3 |  | 183 | 0.16 | 0.70 | 0.984 | 0.974 | 1.000 | 4324 | tags=28%, list=32%, signal=41% |
| 1751 | RGTTAMWNATT\_V$HNF1\_01 |  | 53 | 0.19 | 0.70 | 0.925 | 0.974 | 1.000 | 3504 | tags=26%, list=26%, signal=36% |
| 1752 | AGED\_MOUSE\_CEREBELLUM\_UP |  | 53 | 0.19 | 0.70 | 0.932 | 0.975 | 1.000 | 4426 | tags=40%, list=33%, signal=59% |
| 1753 | TCTGATA,MIR-361 |  | 70 | 0.18 | 0.70 | 0.947 | 0.975 | 1.000 | 4038 | tags=34%, list=30%, signal=49% |
| 1754 | ALKPATHWAY |  | 32 | 0.21 | 0.70 | 0.909 | 0.975 | 1.000 | 6197 | tags=59%, list=47%, signal=111% |
| 1755 | PASSERINI\_ADHESION |  | 36 | 0.20 | 0.70 | 0.924 | 0.977 | 1.000 | 3328 | tags=28%, list=25%, signal=37% |
| 1756 | CMV\_HCMV\_TIMECOURSE\_4HRS\_DN |  | 35 | 0.21 | 0.69 | 0.921 | 0.977 | 1.000 | 1085 | tags=11%, list=8%, signal=12% |
| 1757 | NO1PATHWAY |  | 27 | 0.22 | 0.69 | 0.887 | 0.977 | 1.000 | 3135 | tags=22%, list=24%, signal=29% |
| 1758 | HASLINGER\_B\_CLL\_12 |  | 18 | 0.24 | 0.69 | 0.875 | 0.978 | 1.000 | 3863 | tags=33%, list=29%, signal=47% |
| 1759 | P38MAPKPATHWAY |  | 39 | 0.20 | 0.69 | 0.910 | 0.978 | 1.000 | 2418 | tags=21%, list=18%, signal=25% |
| 1760 | CPR\_NULL\_LIVER\_DN |  | 16 | 0.25 | 0.69 | 0.898 | 0.979 | 1.000 | 2941 | tags=25%, list=22%, signal=32% |
| 1761 | CHR17Q22 |  | 22 | 0.22 | 0.69 | 0.907 | 0.980 | 1.000 | 1226 | tags=14%, list=9%, signal=15% |
| 1762 | CREB\_BRAIN\_8WKS\_UP |  | 69 | 0.18 | 0.69 | 0.958 | 0.980 | 1.000 | 2484 | tags=20%, list=19%, signal=25% |
| 1763 | HOFMANN\_MDS\_CD34\_LOW\_AND\_HIGH\_RISK |  | 46 | 0.19 | 0.69 | 0.939 | 0.979 | 1.000 | 2730 | tags=24%, list=20%, signal=30% |
| 1764 | CHR8Q21 |  | 35 | 0.20 | 0.69 | 0.911 | 0.979 | 1.000 | 1881 | tags=14%, list=14%, signal=17% |
| 1765 | MUNSHI\_MM\_VS\_PCS\_UP |  | 76 | 0.17 | 0.68 | 0.968 | 0.979 | 1.000 | 1625 | tags=13%, list=12%, signal=15% |
| 1766 | CCCACAT,MIR-299-3P |  | 41 | 0.20 | 0.68 | 0.921 | 0.980 | 1.000 | 3494 | tags=24%, list=26%, signal=33% |
| 1767 | CHR1Q44 |  | 17 | 0.24 | 0.68 | 0.888 | 0.980 | 1.000 | 2366 | tags=29%, list=18%, signal=36% |
| 1768 | WANG\_HOXA9\_VS\_MEIS1\_UP |  | 24 | 0.22 | 0.68 | 0.916 | 0.980 | 1.000 | 2881 | tags=25%, list=22%, signal=32% |
| 1769 | STRESS\_ARSENIC\_SPECIFIC\_DN |  | 27 | 0.22 | 0.68 | 0.916 | 0.979 | 1.000 | 1513 | tags=15%, list=11%, signal=17% |
| 1770 | CCR5PATHWAY |  | 17 | 0.24 | 0.68 | 0.900 | 0.980 | 1.000 | 3062 | tags=29%, list=23%, signal=38% |
| 1771 | CHR4Q25 |  | 25 | 0.22 | 0.68 | 0.909 | 0.981 | 1.000 | 3573 | tags=28%, list=27%, signal=38% |
| 1772 | CAGNYGKNAAA\_UNKNOWN |  | 54 | 0.18 | 0.67 | 0.949 | 0.981 | 1.000 | 4680 | tags=39%, list=35%, signal=60% |
| 1773 | RYTGCNNRGNAAC\_V$MIF1\_01 |  | 49 | 0.19 | 0.67 | 0.960 | 0.981 | 1.000 | 3122 | tags=24%, list=23%, signal=32% |
| 1774 | CHR3Q12 |  | 18 | 0.23 | 0.67 | 0.889 | 0.981 | 1.000 | 895 | tags=11%, list=7%, signal=12% |
| 1775 | TPA\_RESIST\_LATE\_UP |  | 36 | 0.20 | 0.67 | 0.929 | 0.981 | 1.000 | 4627 | tags=44%, list=35%, signal=68% |
| 1776 | KANG\_TERT\_DN |  | 95 | 0.16 | 0.66 | 0.986 | 0.985 | 1.000 | 3778 | tags=28%, list=28%, signal=39% |
| 1777 | V$PAX5\_01 |  | 108 | 0.16 | 0.66 | 0.996 | 0.985 | 1.000 | 4659 | tags=36%, list=35%, signal=55% |
| 1778 | HBX\_NL\_DN |  | 16 | 0.24 | 0.66 | 0.903 | 0.985 | 1.000 | 4283 | tags=44%, list=32%, signal=64% |
| 1779 | V$GRE\_C |  | 91 | 0.16 | 0.66 | 0.979 | 0.985 | 1.000 | 4436 | tags=34%, list=33%, signal=51% |
| 1780 | CHR3P25 |  | 44 | 0.19 | 0.65 | 0.959 | 0.986 | 1.000 | 4225 | tags=36%, list=32%, signal=53% |
| 1781 | WONG\_IFNA\_HCC\_RESISTANT\_VS\_SENSITIVE\_UP |  | 15 | 0.24 | 0.65 | 0.911 | 0.986 | 1.000 | 5399 | tags=53%, list=41%, signal=90% |
| 1782 | ST\_INTERLEUKIN\_4\_PATHWAY |  | 26 | 0.20 | 0.65 | 0.929 | 0.987 | 1.000 | 2741 | tags=27%, list=21%, signal=34% |
| 1783 | CHR2Q23 |  | 16 | 0.23 | 0.65 | 0.919 | 0.988 | 1.000 | 2268 | tags=25%, list=17%, signal=30% |
| 1784 | V$PPARG\_01 |  | 33 | 0.19 | 0.64 | 0.951 | 0.989 | 1.000 | 5645 | tags=52%, list=42%, signal=89% |
| 1785 | NUCLEAR\_RECEPTORS |  | 40 | 0.18 | 0.63 | 0.967 | 0.991 | 1.000 | 2576 | tags=20%, list=19%, signal=25% |
| 1786 | LINDSTEDT\_DEND\_DN |  | 64 | 0.17 | 0.63 | 0.982 | 0.992 | 1.000 | 3294 | tags=23%, list=25%, signal=31% |
| 1787 | NADLER\_OBESITY\_DN |  | 33 | 0.19 | 0.63 | 0.957 | 0.991 | 1.000 | 4012 | tags=33%, list=30%, signal=48% |
| 1788 | IFNALPHA\_NL\_UP |  | 27 | 0.20 | 0.63 | 0.958 | 0.992 | 1.000 | 3118 | tags=26%, list=23%, signal=34% |
| 1789 | TPOPATHWAY |  | 23 | 0.20 | 0.62 | 0.943 | 0.992 | 1.000 | 5577 | tags=57%, list=42%, signal=97% |
| 1790 | CHR2Q21 |  | 33 | 0.19 | 0.62 | 0.953 | 0.993 | 1.000 | 3062 | tags=21%, list=23%, signal=27% |
| 1791 | CHRXQ13 |  | 35 | 0.18 | 0.62 | 0.970 | 0.992 | 1.000 | 2990 | tags=17%, list=22%, signal=22% |
| 1792 | CHESLER\_BRAIN\_NEURAL\_HIGH\_GENES |  | 21 | 0.20 | 0.62 | 0.947 | 0.993 | 1.000 | 4954 | tags=52%, list=37%, signal=83% |
| 1793 | AGUIRRE\_PANCREAS\_CHR7 |  | 48 | 0.17 | 0.61 | 0.978 | 0.993 | 1.000 | 2437 | tags=19%, list=18%, signal=23% |
| 1794 | GLEEVECPATHWAY |  | 22 | 0.20 | 0.60 | 0.962 | 0.995 | 1.000 | 1775 | tags=18%, list=13%, signal=21% |
| 1795 | HEARTFAILURE\_ATRIA\_UP |  | 25 | 0.19 | 0.60 | 0.959 | 0.995 | 1.000 | 4654 | tags=32%, list=35%, signal=49% |
| 1796 | V$PAX4\_03 |  | 188 | 0.13 | 0.60 | 0.999 | 0.995 | 1.000 | 4259 | tags=30%, list=32%, signal=43% |
| 1797 | V$PPARA\_02 |  | 92 | 0.14 | 0.58 | 0.999 | 0.998 | 1.000 | 5229 | tags=39%, list=39%, signal=64% |
| 1798 | CHR10P11 |  | 15 | 0.21 | 0.58 | 0.971 | 0.998 | 1.000 | 5680 | tags=53%, list=43%, signal=93% |
| 1799 | MCALPAINPATHWAY |  | 22 | 0.19 | 0.58 | 0.971 | 0.997 | 1.000 | 3460 | tags=23%, list=26%, signal=31% |
| 1800 | WANG\_HOXA9\_VS\_MEIS1\_DN |  | 21 | 0.19 | 0.57 | 0.972 | 0.998 | 1.000 | 4392 | tags=38%, list=33%, signal=57% |
| 1801 | IGF\_VS\_PDGF\_UP |  | 57 | 0.15 | 0.57 | 0.994 | 0.998 | 1.000 | 5770 | tags=46%, list=43%, signal=80% |
| 1802 | TGF\_BETA\_SIGNALING\_PATHWAY |  | 48 | 0.15 | 0.56 | 0.991 | 0.998 | 1.000 | 3579 | tags=23%, list=27%, signal=31% |
| 1803 | SCHURINGA\_STAT5A\_UP |  | 22 | 0.18 | 0.56 | 0.981 | 0.998 | 1.000 | 4345 | tags=36%, list=33%, signal=54% |
| 1804 | CHR5Q32 |  | 17 | 0.20 | 0.56 | 0.977 | 0.998 | 1.000 | 4757 | tags=47%, list=36%, signal=73% |
| 1805 | CRGAARNNNNCGA\_UNKNOWN |  | 33 | 0.17 | 0.55 | 0.981 | 0.998 | 1.000 | 1727 | tags=12%, list=13%, signal=14% |
| 1806 | HCMVPATHWAY |  | 15 | 0.19 | 0.54 | 0.987 | 0.999 | 1.000 | 3460 | tags=27%, list=26%, signal=36% |
| 1807 | CHR8Q24 |  | 91 | 0.13 | 0.54 | 0.999 | 0.998 | 1.000 | 2463 | tags=15%, list=18%, signal=19% |
| 1808 | HDACPATHWAY |  | 29 | 0.16 | 0.53 | 0.994 | 0.999 | 1.000 | 5631 | tags=41%, list=42%, signal=72% |
| 1809 | TFF2\_KO\_UP |  | 22 | 0.17 | 0.52 | 0.988 | 0.998 | 1.000 | 5915 | tags=55%, list=44%, signal=98% |
| 1810 | ZHAN\_MM\_CD138\_CD2\_VS\_REST |  | 31 | 0.16 | 0.51 | 0.998 | 0.999 | 1.000 | 5140 | tags=39%, list=39%, signal=63% |
| 1811 | METPATHWAY |  | 33 | 0.15 | 0.50 | 0.997 | 0.999 | 1.000 | 3460 | tags=24%, list=26%, signal=33% |
| 1812 | BADPATHWAY |  | 21 | 0.16 | 0.49 | 0.994 | 0.999 | 1.000 | 3747 | tags=24%, list=28%, signal=33% |
| 1813 | KIM\_TH\_CELLS\_DN |  | 15 | 0.16 | 0.43 | 1.000 | 1.000 | 1.000 | 11221 | tags=100%, list=84%, signal=634% |
Table: Gene sets enriched in phenotype **na **[plain text format]****
